# Experimental and Theoretical Investigation of the Mechanism of the Reduction of O_2_ from Air to O_2_^2–^ by V^IV^O^2+^–*N*,*N*,*N*-Amidate Compounds and Their Potential Use in Fuel Cells

**DOI:** 10.1021/acs.inorgchem.3c03272

**Published:** 2024-02-06

**Authors:** Michael Papanikolaou, Sofia Hadjithoma, Odysseas Keramidas, Chryssoula Drouza, Angelos Amoiridis, Alexandros Themistokleous, Sofia C. Hayes, Haralampos N. Miras, Panagiotis Lianos, Athanassios C. Tsipis, Themistoklis A. Kabanos, Anastasios D. Keramidas

**Affiliations:** † Department of Chemistry, University of Cyprus, Nicosia 2109, Cyprus; ‡ Department of Agricultural Sciences, Biotechnology and Food Science, Cyprus University of Technology, Limassol 3036, Cyprus; § School of Chemistry, The University of Glasgow, Glasgow G12 8QQ, U.K.; ∥ Department of Chemical Engineering, University of Patras, 26500 Patras, Greece; ⊥ Section of Inorganic and Analytical Chemistry, Department of Chemistry, University of Ioannina, 45110 Ioannina, Greece

## Abstract

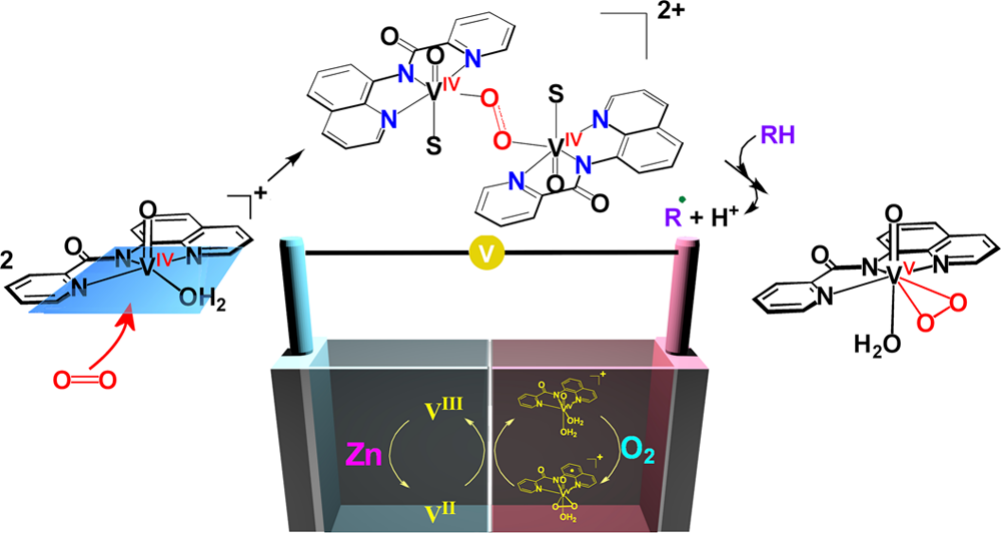

The two-electron reductive activation of O_2_ to O_2_^2–^ is of particular interest to the scientific community mainly due to the use of peroxides as green oxidants and in powerful fuel cells. Despite of the great importance of vanadium(IV) species to activate the two-electron reductive activation of O_2_, the mechanism is still unclear. Reaction of V^IV^O^2+^ species with the tridentate-planar *N,N,N-*carboxamide (ΗL) ligands in solution (CH_3_OH:H_2_O) under atmospheric O_2_, at room temperature, resulted in the quick formation of [V^V^(=O)(η^2^-O_2_)(κ^3^-L)(H_2_O)] and *cis*-[V^V^(=O)_2_(κ^3^-L)] compounds. Oxidation of the V^IV^O^2+^ complexes with the sterically hindered tridentate-planar *N,N,N-*carboxamide ligands by atmospheric O_2_ gave only *cis*-[V^V^(=O)_2_(κ^3^-L)] compounds. The mechanism of formation of [V^V^(=O)(η^2^-O_2_)(κ^3^-L)(H_2_O)] (I) and *cis*-[V^V^(=O)_2_(κ^3^-L)] (II) complexes vs time, from the interaction of [V^IV^(=O)(κ^3^-L)(Η_2_Ο)_2_]^+^ with atmospheric O_2_, was investigated with ^51^V, ^1^H NMR, UV–vis, cw-X-band EPR, and ^18^O_2_ labeling IR and resonance Raman spectroscopies revealing the formation of a stable intermediate (**Id**). EPR, MS, and theoretical calculations of the mechanism of the formation of I and II revealed a pathway, through a binuclear [V^IV^(=O)(κ^3^-L)(H_2_O)(η^1^,η^1^-O_2_)V^IV^(=O)(κ^3^-L)(H_2_O)]^2+^ intermediate. The results from cw-EPR, ^1^H NMR spectroscopies, cyclic voltammetry, and the reactivity of the complexes [V^IV^(=O)(κ^3^-L)(Η_2_Ο)_2_]^+^ toward O_2_ reduction fit better to an intermediate with a binuclear nature. Dynamic experiments in combination with computational calculations were undertaken to fully elucidate the mechanism of the O_2_ reduction to O_2_^2–^ by [V^IV^(=O)(κ^3^-L)(Η_2_Ο)_2_]^+^. The galvanic cell {Zn|V^III^,V^II^||**Id**, [V^IV^O(κ^3^-L)(H_2_O)_2_]^+^|O_2_|C(s)} was manufactured, demonstrating the important applicability of this new chemistry to Zn|H_2_O_2_ fuel cells technology generating H_2_O_2_ in situ from the atmospheric O_2_.

## Abstract 2

The two-electron reductive activation of O_2_ to O_2_^2−^ is of particular interest to the scientific community mainly due to the use of peroxides as green oxidants and in powerful fuel cells. The manufactured galvanic cell {Zn|V^III^,V^II^||**Id**, [V^IV^O(κ^3^-L)(H_2_O)_2_]^+^|O_2_|C(s)} demonstrates the important applicability of this new chemistry to Zn|H_2_O_2_ fuel cells technology generating H_2_O_2_ in situ from the atmospheric O_2_.

## Introduction

Reductive O_2_ activation via oxidative addition of the molecular dioxygen to a transition metal center is a central reaction in biological processes.^1−4^ Of particular interest is the selective two-electron of O_2_ reduction to H_2_O_2_ because H_2_O_2_ is a green renewable source of energy, producing environmentally friendly exhaust gases H_2_O and O_2_ after its decomposition, and represents a valuable commodity chemical, a versatile, and clean oxidizing agent.^5−21^ The vanadium(V)–peroxido species take part in various catalytic oxidations including industrial processes, organic synthesis, electrochemical cells, enzymatic reactions, pharmaceutical applications, and emerging energy technologies.^22−37^

Metal-air batteries have gained significant renewable interest as a solution to energy storage, due to their high theoretical energy densities which are much higher than the densities of lithium batteries.^38−46^ In particular, zinc-air batteries are considered as promising replacement for lithium batteries because they are safe, environmentally friendly, with a high energy density (theoretical value 1086 Wh kg^–1^), and low cost.^39^ However, zinc-air secondary batteries lack extensive commercialization due to drawbacks such as zinc anode degradation and O_2_ activation.^38,41,44,47^ Recently, An and co-workers have demonstrated a cheap zinc–hydrogen peroxide fuel cell, of high performance, designed to propel vehicles.^37^ They combined zinc–hydrogen peroxide with a vanadium redox flow cell, consisting of the redox couples V(II)/V(III) at the anode, and V(IV)/V(V) at the cathode regenerated by zinc and hydrogen peroxide, respectively. These processes in fuel cells and metal–hydrogen peroxide batteries convert chemical to electrical energy, and the replacement of the expensive H_2_O_2_ with the “greener” and inexhaustible O_2_ is highly desirable.

However, O_2_ is an inert oxidant and the current air electrodes show low catalytic activities for the oxygen reduction reaction (ORR).^48^ The development of effective ORR catalysts which may speed up the electron transfer from the electrodes to electrolyte-dissolved species is of high importance to clean energy technologies, such as fuel cells.^44,47^ One of the major challenges of ORR catalysts is to overcome the developing overpotential mainly due to the thermodynamically unfavorable one-electron reduction of O_2_.^48^ In addition, for the synthesis of H_2_O_2_ from O_2_, the ORR catalyst should selectively promote the 2e^–^ vs the 4e^–^ reduction of O_2_ (eqs 1 and 2).
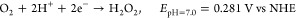
1
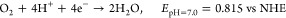
2

The selective 2e^–^ reductive activation of O_2_ from the metal compounds requires the metal catalysts not to exhibit any catalase activity (disproportionation of H_2_O_2_ to H_2_O and O_2_), and to thermodynamically stabilize O_2_^2–^. One choice is the use of vanadium(IV) species which can selectively stabilize O_2_^2–^ by forming vanadium(V) peroxido complexes.

Even though, vanadium(V) is oxophilic, when it is bound to peroxido or hydroxylamido ligands softens, preferring nitrogen ligation over oxygen.^49−52^ Another impressive property of the vanadium(V)-peroxido compounds is the formation of hydrolytically very stable compounds with the deprotonated peptide nitrogen atom, in marked contrast to the dioxidovanadium(V) species which interact only weekly with peptide bonds.^50,53^ Apparently, ligands with nitrogen and -N- peptide donor atoms bound to vanadium will stabilize thermodynamically the O_2_^2–^, leading to 2e^–^ selective reduction of O_2_.

**Scheme 1:**
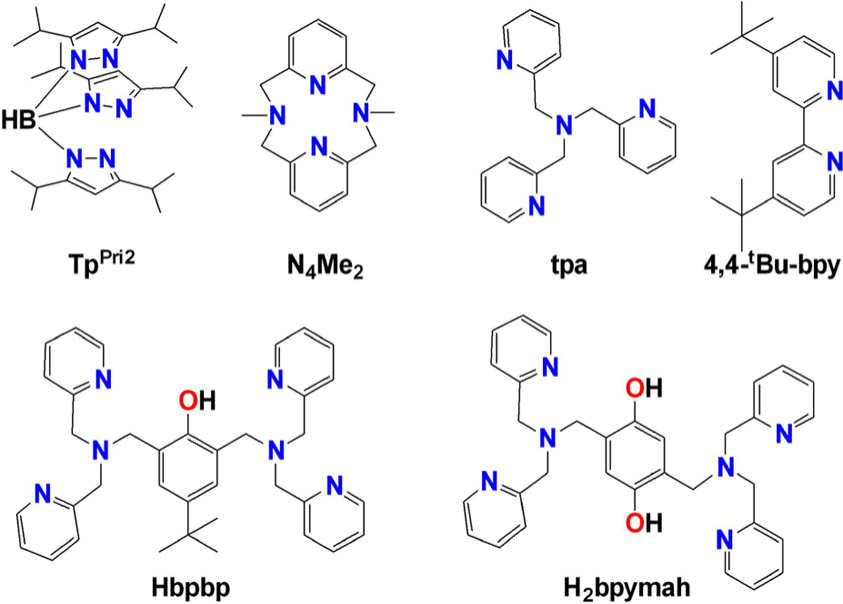
Nitrogeneous Ligands Reported in the Literature and Used for the Syntheses of [V^IV^O]^2+^ Compounds which Reduce Dioxygen to O_2_^2–^

For the development of efficient and selective ORR, it is of vital importance to fully elucidate the mechanism of O_2_ reduction of O_2_^2–^ by V^IV^O^2+^ species. Kelm and Kruger have suggested a superoxido centered radical intermediate, based on the EPR spectrum of the electrochemically generated [V^V^(=O)(η^2^-O_2_)(N_4_Me_2_)]^·^^2+^ radical {N_4_Me_2_ = 3,7-dimethyl-3,7-diaza-1,5(2,6)-dipyridinacyclooctaphane} (Scheme 1) from [V^V^(=O)(η^2^-O_2_)(N_4_Me_2_)]^+^.^54^ However, the existence of such an intermediate has been questioned since the [V^V^(=O)(η^2^-O_2_)(N_4_Me_2_)]^·2+^ radical was generated electrochemically and not from the direct reaction of [V^IV^(=O)(N_4_Me_2_)]^2+^ with O_2_.^55^

Reviewing the literature for the V^IV^ reductive activators of O_2_, it is evident that the V^IV^ complexes containing nitrogeneous ligands (Scheme 1) activate the 2e^–^ reduction of O_2_ to O_2_^2–^. However, the V^IV^ complexes with neutral nitrogeneous ligands activate O_2_ only in THF,^56,55,54^ and this fact was attributed in some cases to peroxido contaminants of the solvent.^56^ On the other hand, V^IV^ complexes with negatively charged nitrogeneous ligands are much more effective toward reductive O_2_ activation, in other organic solvents besides THF.^58,57,59^

**Scheme 2:**
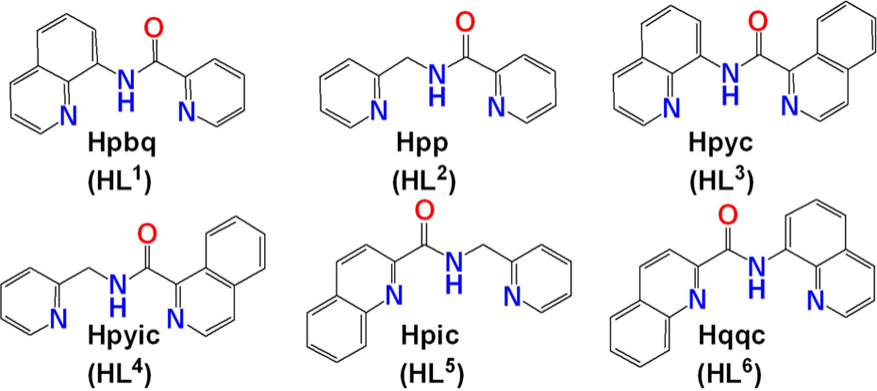
Molecular Drawings of the Carboxamide Ligands (HL^1^^–6^) Used in This Study

Inspired by the features of the ligands reported in the literature, we embarked on an effort to synthesize ligands with three nitrogen donor atoms (one of which is an amide nitrogen), planar with delocalized π bonding system and −1 charge upon deprotonation of the amide nitrogen atom. More specifically, the following ligands were synthesized: {*N*-(quinolin-8-yl)picolinamide, Hpbq(HL^1^); *N*-(pyridin-2-ylmethyl)picolinamide, Hpp(HL^2^); *N*-(quinolin-8-yl)isoquinoline-1-carboxamide, Hpyc(HL^3^); *N*-(pyridin-2-ylmethyl)isoquinoline-1-carboxamide, Hpyic(HL^4^); *N*-(pyridin-2-ylmethyl)quinoline-2-carboxamide, Hpic(HL^5^); and *N*-(quinolin-8-yl)quinoline-2-carboxamide, Hqqc(HL^6^)} (Scheme 2).^54−61^

**Scheme 3:**
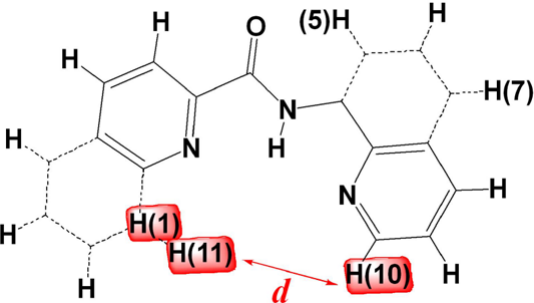
Steric Hindrance Introduced to the System by Changing the Position of the Benzene Rings

Herein, we report on the first systematic study of the mechanism of O_2_ reduction by V^IV^O^2+^ species with the ligands HL^1–6^. The ligands HL^1–6^ upon deprotonation of the amide nitrogen atom obtain a variety of extensive delocalized π*-*systems, and this fact is of vital importance in the study for the stability of possible intermediate V^V^–O_2_^•^ radical species. By judiciously changing the position of the benzene rings, we introduced steric hindrance to the plane defined by the planar ligands (Scheme 3), thus getting further stereochemical information related to the first steps of O_2_ binding to the metal ion.

The reaction of the V^IV^O^2+^ species with the carboxamide ligands HL^1–4^ in the presence of air gave [V^V^(=O)(η^2^-O_2_)(κ^3^-L^1–4^)(H_2_O)] and *cis*-[V^V^O_2_(κ^3^-L^1–4^)] complexes. The reaction of V^IV^O^2+^ with the carboxamide sterically hindered ligands HL^5,6^ gave only *cis*-[V^V^O_2_(κ^3^-L^5,6^)]. The intermediate species toward the formation of [V^V^(=O)(η^2^-O_2_)(κ^3^-L^1–4^)(H_2_O)] and/or *cis*-[V^V^(=O)_2_(κ^3^-L^1–4^)] has been fully characterized, by ^1^H nuclear magnetic resonance (^1^H NMR), electron paramagnetic resonance (EPR), Fourier transform infrared (FTIR), Raman, and UV–vis spectroscopies and electrochemistry. The mechanism of the reaction of [V^IV^(=O)(κ^3^-L^1–6^)(H_2_O)_2_]^+^ with O_2_ was also investigated by theoretical calculations which revealed two possible pathways, either through a mononuclear intermediate radical [V^V^(=O)(η^2^-O_2_)(L)]^·+^ or through a binuclear [V^IV^(=O)(κ^3^-L)(H_2_O)(η^1^,η^1^-O_2_)V^IV^(=O)(κ^3^-L)(H_2_O)]^2+^. The experimental results from cw-EPR, ^1^H NMR spectroscopies, cyclic voltammetry, and the reactivity of the complexes toward O_2_ reduction fit better to an intermediate with a binuclear nature. The sterically hindered HL^5,6^ ligands prevent the approach of O_2_ to V^IV^, inhibiting the activity of the complex [V^IV^(=O)(κ^3^-L^5,6^)(H_2_O)_2_]^+^ toward O_2_ reduction. The intermediates are stronger oxidants than vanadium peroxido species [V^V^(=O)(η^2^-O_2_)(κ^3^-L^1–4^)(H_2_O)], suggesting that the intermediates might be better oxidative catalysts for the activation of hydrocarbons than peroxido-vananadium(V) compounds. Finally, a galvanic cell has been constructed demonstrating that these materials can be used in the vanadium-Zn|H_2_O_2_ fuel cells leading to in situ synthesis of H_2_O_2_ from the atmospheric O_2_.

## Experimental Section

No uncommon hazards are noted.

### Synthesis of the Vanadium Complexes

#### *N*-(Quinolin-8-yl)picolinamido-(*N*_*q*_*,N*_*am*_*,N*_*py*_)}(aqua)(peroxido)oxidovanadium(V), [V^V^(=O)(η^2^-O_2_)(κ^3^-pbq-*N*_*q*_*,N*_*am*_*,N*_*py*_)(H_2_O)]·H_2_O (**1**·H_2_O)

To a stirred solution of V^IV^OSO_4_·3.5H_2_O (0.0036 g, 0.016 mmol) in H_2_O (250 μL) was added a methanol solution (750 μL) of Hpbq (0.0039 g, 0.016 mmol), 10 min later the color of the solution changed from yellow-green to red. The solution was stirred for 3 h and was left undisturbed at room temperature (22 °C) for 2 days. During this period of time, red crystals of **1** were precipitated out and filtered under vacuum. Yield: 0.0028 g (46%, based on Hpbq). Anal. calcd for **1·**H_2_O, [C_15_H_14_N_3_O_6_V] (*M*_r_ = 383.23)]: C, 47.01; H, 3.68; N, 10.96. Found: C, 46.91; H, 3.69; N, 10.91.

Crystals of [V^V^(=O)(η^2^-^18^O_2_)(κ^3^-pbq-*N*_*q*_*,N*_*am*_*,N*_*py*_)(H_2_O)]·H_2_O were obtained by reacting V^IV^OSO_4_·3.5H_2_O with Hpbq in methanol:H_2_O (3:1) solutions under an ^18^O_2_ atmosphere.
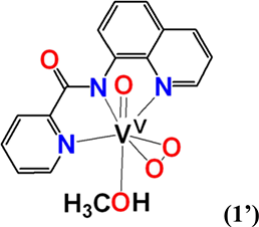


#### [V^V^(=O)(η^2^-O_2_)(κ^3^-pbq-*N*_*q*_*,N*_*am*_*,N*_*py*_)(CH_3_OH)] (**1′**)

Complex **1′** was synthesized using the same method reported for **1**·H_2_O by reacting either V^IV^OSO_4_·3.5H_2_O or V^IV^OCl_2_ with Hpbq in pure methanol. Yield: (42%, based on Hpbq). Anal. calcd for **1′**, [C_16_H_14_N_3_O_5_V] (*M*_r_ = 379.24)]: C, 50.67; H, 3.72; N, 11.08. Found: C, 50.81; H, 3.71; N, 10.96.
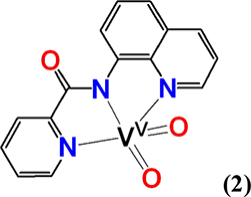


#### *cis*-[V^V^(=O)_2_(κ^3^-pbq-*N*_*q*_*,N*_*am*_*,N*_*py*_)] (**2**)

Compound **1·**H_2_O (0.0038 g, 0.0099 mmol) was dissolved in CH_3_OH (500 μL) under magnetic stirring. Then, H_2_O (500 μL) was added to it, and the mixture was heated to boil. The yellow precipitate was filtered under vacuum and dried. Yellow crystals of **2** were obtained from slow evaporation of a concentrated methanol solution of **2**. Yield: 0.0018 g (55% based on **1·**H_2_O). Anal. calcd for **2**, [C_15_H_10_N_3_O_3_V] (*M*_r_ = 331.02)]: C, 54.40; H, 3.04; N, 12.69. Found: C, 54.32; H, 3.03; N, 12.76.
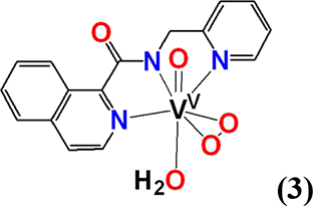


#### [V^V^(=O)(η^2^-O_2_)(κ^3^-pyic-*N*_*py*_*,N*_*am*_*,N*_*q*_)(H_2_O)] (**3**)

Compound **3** was synthesized using the same method reported for **1**·H_2_O by reacting either V^IV^OSO_4_·3.5H_2_O or V^IV^OCl_2_ in H_2_O with Hpyic in methanol. Yield (41%, based on Hpyic). Anal. calcd for **3**, [C_16_H_14_N_3_O_5_V] (*M*_r_ = 379.24)]: C, 50.67; H, 3.72; N, 11.08. Found: C, 50.74; H, 3.80; N, 10.89.
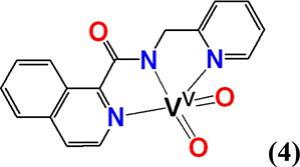


#### *cis*-[V^V^(=O)_2_(κ^3^-pyic-*N*_*py*_*,N*_*am*_*,N*_*q*_)] (**4**)

To a stirred solution of V^IV^OSO_4_·3.5H_2_O (0.0036 g, 0.016 mmol) in H_2_O (300 μL), a solution of Hpyic (0.0042 g, 0.016 mmol) in methanol (700 μL) was added. The resulting solution was refluxed for 10 min and its color changed from yellow-green to yellow. Then, it was kept at room temperature for 2 days, and yellow crystals of **4** were precipitated out and filtered under vacuum. Yield 0.0029 g (49%, based on Hpyic). Anal. calcd for **4**, [C_16_H_12_N_3_O_3_V] (*M*_r_ = 363.25(*M*_r_ = 345.23)]: C, 55.67; H, 3.50; N, 12.17. Found: C, 55.43; H, 3.62; N, 12.38.
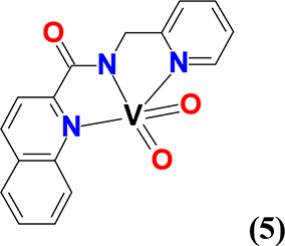


#### *cis*-[V^V^(=O)_2_[(κ^3^-pic-*N*_*q*_*,N*_*am*_*,N*_*py*_)] (**5**)

Compound **5** was synthesized using the same method reported for **4**. Yield: (48%, based on Hpic). Anal. calcd or **5**, [C_16_H_12_N_3_O_3_V] (*M*_r_ = 345.23)]: C, 55.67; H, 3.50; N, 12.17. Found: C, 55.03; H, 3.33; N, 12.24.
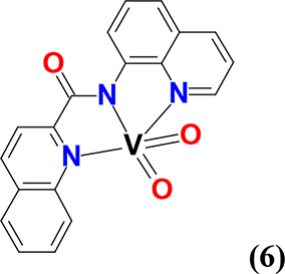


#### *cis*-[V^V^(=O)_2_(κ^3^-qqc-*N*_*q*_*,N*_*am*_*,N*_*q*_)] (**6**)

Compound **6** was synthesized using the same method reported for **4**. Yield: (48%, based on Hqqc). Anal. calcd for **6**, [C_19_H_12_N_3_O_3_V] (*M*_r_ = 381.26)]: C, 59.86; H, 3.17; N, 11.02. Found: C, 59.63; H, 3.21; N, 11.17.
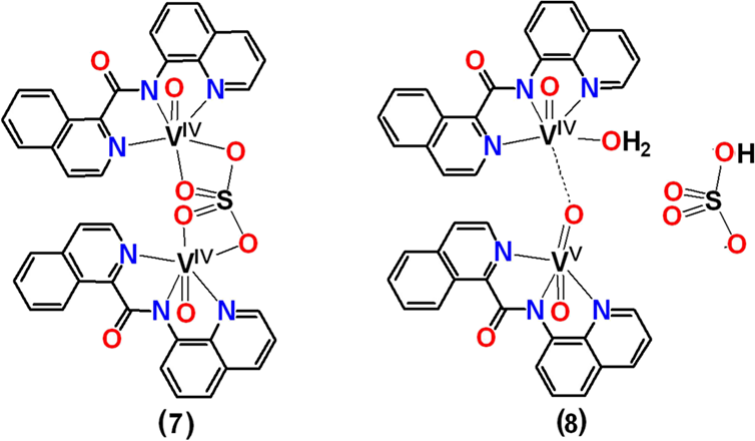


#### {[V^IV^(=O)]_2_(κ^3^-qqc-*N*_*q*_*,N*_*am*_*,N*_*q*_)_2_](η^2^:η^2^_:_μ-SO_4_)]} (**7**) (Method A) and [V^IV^(=O)(κ^3^-*N*_*q*_*,N*_*am*_*,N*_*q*_)-(qqc)](μ-O)[V^V^O(κ^3^-qqc-*N*_*q*_*,N*_*am*_*,N*_*q*_)]HSO_4_·H_2_O (**8**)

To a stirred solution of V^IV^OSO_4_·3.5H_2_O (0.0036 g, 0.016 mmol) in H_2_O (300 μL), a methanol solution (700 μL) of Hqqc (0.0042 g, 0.016 mmol) was added. High-purity argon was bubbled through the solution for 5 min. Then, the solution was refluxed for 10 min. Upon refluxing it, its yellow-green color changed to yellow-brown. The solution was kept at room temperature (20 °C) for 2 days undisturbed, and brown crystals were precipitated out and filtered under vacuum. The brown crystals of different morphologies, blocks, and needles for **7** and **8**, respectively, were separated under the microscope.

#### {[V^IV^(=O)]_2_(κ^3^-qqc-*N*_*q*_*,N*_*am*_*,N*_*q*_)_2_](η^2^:η^2^_:_μ-SO_4_)]} (**7**). Method B

Complex **7** was synthesized by heating up to boil, under argon, a H_2_O:MeOH (30:70 v/v) solution of V^IV^OSO_4_·3.5H_2_O (0.0036 g, 0.016 mmol) and Hqqc (0.0042 g, 0.016 mmol). On cooling the solution, a brown precipitate of **7** was formed and filtered under vacuum. Yield 0.0041 g (62%, based on Hqqc). Anal. calcd for **7**, [C_38_H_24_N_6_O_8_SV_2_] (*M*_r_ = 829.59)]: C, 55.22; H, 2.93; N, 10.17. Found: C, 55.37; H, 3.02; N, 10.09.
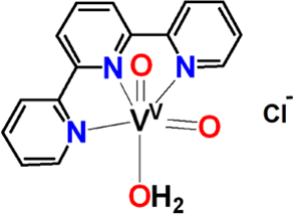


#### 2,2’:6′,2″-Terpyridine(*N*_*py*_*,N*_*py*_*,N*_*py*_)*cis*-dioxidovanadium(V) Chloride, *cis*-[V^V^(=O)_2_(κ^3^-terpy-*N*_*py*_*,N*_*py*_*,N*_*py*_)]Cl·CH_3_OH (**9**·CH_3_OH)

Compound **9·**CH_3_OH was synthesized using the same method reported for **4**. Yield: (45%, based on terpy). Anal. calcd for **9·**CH_3_OH, [C_16_H_15_ClN_3_O_3_V] (*M*_r_ = 383.71)]: C, 50.08; H, 3.94; N, 10.95. Found: C, 49.88; H, 3.79; N, 10.82.

#### Computational Details

All calculations were performed using the Gaussian09, D.01 program suite.^62^ The geometries and thermal corrections for all stationary points along the reaction coordinate are computed with the Perdew, Burke, and Ernzerhof^63−69^ of hybrid density functional denoted as PBE0 (also called PBE1PBE) as implemented in the Gaussian09 program suite. For the geometry optimizations, we have used the Def2-TZVP basis set^70^ for the vanadium central atom and the 6-31+G(d) basis set for all main group elements (E). Hereafter, the method used in DFT calculations is abbreviated as PBE0/Def2-TZVP(V)∪6-31+G(d)(E). Frequency calculations were also performed at the same level of theory to identify whether the stationary point is a local minimum or a transition state. The transition states were confirmed by IRC calculations and had only one imaginary frequency. The natural bond orbital (NBO) population analysis was performed using Weinhold’s methodology as implemented in the NBO 6.0 software.^71−73^ All calculations were performed for aqueous solutions employing the Polarizable Continuum Model (PCM) using the integral equation formalism variant (IEFPCM) being the default self-consistent reaction field (SCRF) method.^74^ The calculated free energy of the proton is −0.174563 a.u.

The redox potential of [V(=O)(η^2^-O_2_)(κ^3^-pbq)(H_2_O)]^+^ complex has been calculated based on the Born–Haber cycle depicted below:
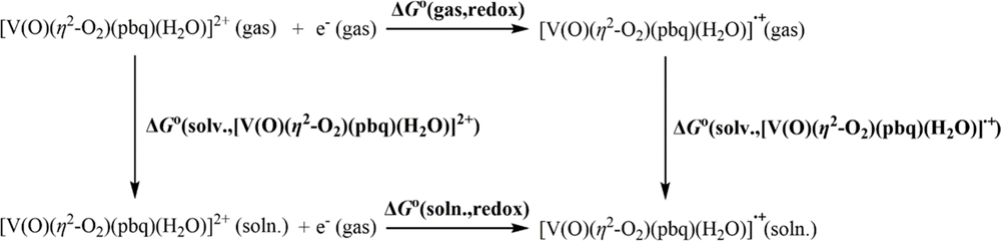
The redox potential is then calculated according to the following equations:
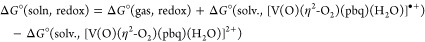
and
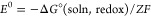
where *F* is the Faraday constant (23.061 kcal per volt gram equivalent) and *Z* is unity since we consider only one-electron redox processes.

## Results and Discussion

### Synthesis of the Vanadium Complexes

**Scheme 4:**
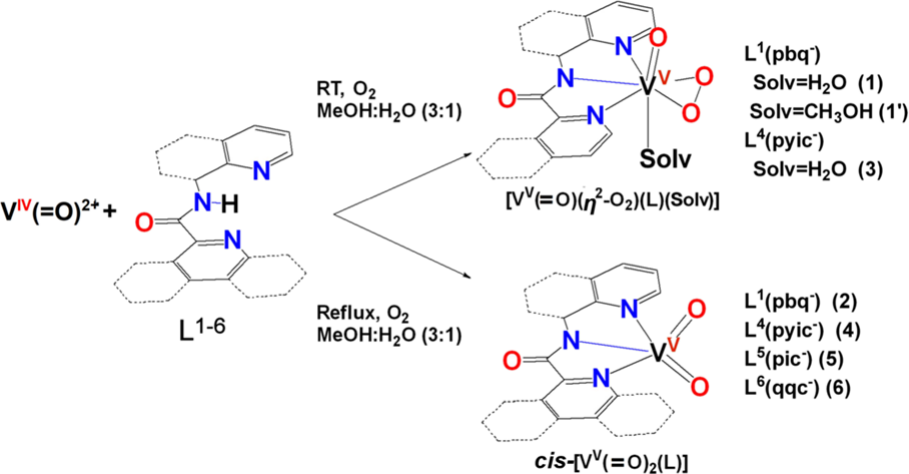
Synthesis of Compounds **1**·H_2_O-**6**

**Scheme 5:**
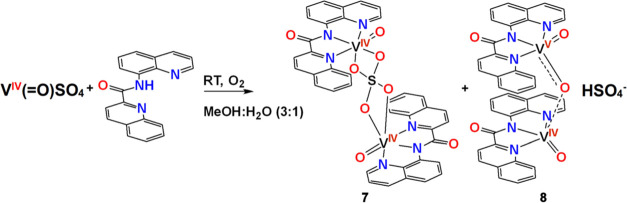
Synthesis of the Dinuclear Vanadium Compounds **7** and **8**

Reaction of equimolar quantities of V^IV^O^2+^ with either Hpbq or Hpyic in CH_3_OH:H_2_O (3:1 v/v) in the presence of air, at room temperature (22 °C), resulted in the isolation of the compounds of the general formula [V^V^(=O)(η^2^-O_2_)(κ^3^-L^1,4^)(H_2_O)] [L^1^ = pbq^–^ (**1**), L^4^ = pyic^–^ (**3**), Scheme 4, eq 3]. Efforts to isolate the vanadium(V) complexes with the ligands Hpp (HL^2^) and Hpyc (HL^3^) were unsuccessful either due to coprecipitation of [V^V^(=O)(η^2^-O_2_)(κ^3^-L^2^)(H_2_O)] with *cis*-[V^V^(=O)_2_(κ^3^-L^2^)] or due to low solubility of Hpyc, resulting in precipitations of [V^V^(=O)(η^2^-O_2_)(κ^3^-L^3^)(H_2_O)] containing small quantities of the free ligand as it was evidenced by ^1^H NMR. The reaction of the ligands Hpbq, Hpyic, Hpic, and Hqqc with V^IV^O^2+^ at refluxing CH_3_OH:H_2_O (3:1 v/v) yielded the compounds of the general formula *cis*-[V^V^(=O)_2_(κ^3^-L^1,4–6^)] [Scheme 4, eq 4]. Reaction of V^IV^(=O)SO_4_·3.5H_2_O with Hqqc under air resulted in the isolation of poor-quality crystals of the dinuclear compounds, such as V^IV^_2_ (**7**) and the mixed-valence V^IV/V^_2_ (**8**) (Figure S1) (Scheme 5). V^IV^_2_ (**7**) was also prepared by reacting V^IV^(=O)SO_4_·3.5H_2_O with Hqqc in MeOH under the Ar atmosphere.

Efforts to synthesize the compounds [V^V^(=O)(η^2^-O_2_)(κ^3^-L^5,6^)(H_2_O)] (L^5^ = pic^–^, L^6^ = qqc^–^) even by reacting *cis*-[V^V^(=O)_2_(pic/qqc)] with large excess of H_2_O_2_ were unsuccessful. This failure might be attributed to steric effects (Scheme 3).


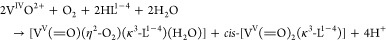
3
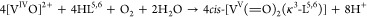
4

### Crystal Structures

**Figure 1:**
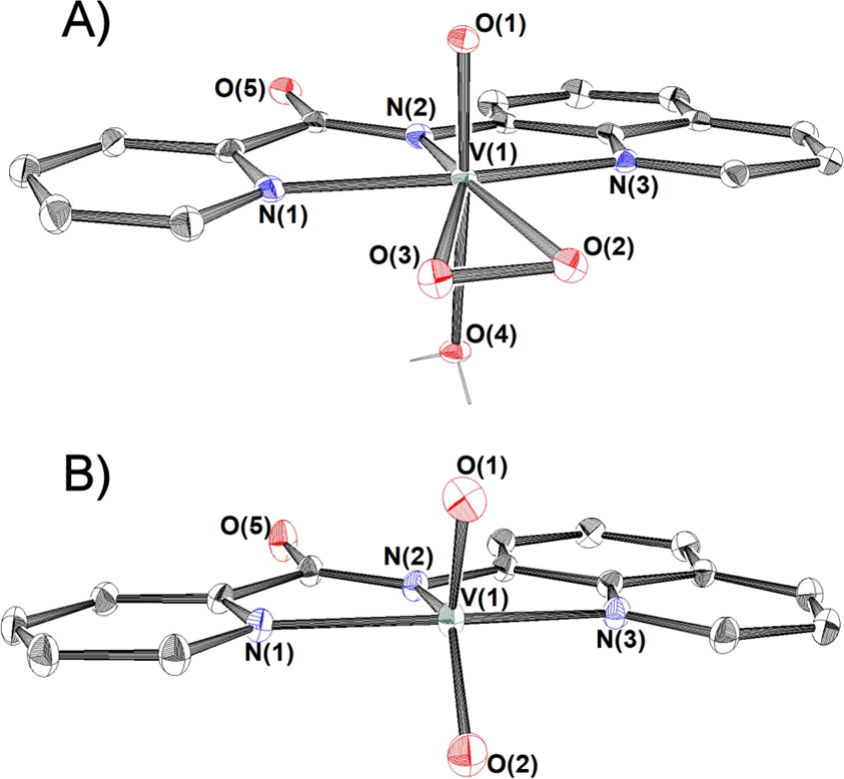
ORTEP plots of **1**·H_2_O (A, the oxido/peroxido derivative) and **2** (B, the dioxido derivative), with 50% thermal ellipsoids. The hydrogen atoms on carbon atoms, and the water of crystallization, have been omitted for clarity. Selected bond lengths (Å) and angles (°): Compound **1**·H_2_O: V(1)–O(1) 1.599(2), V(1)–O(2) 1.888(2), V(1)–O(3) 1.888(2), V(1)–O(4) 2.243(2), V(1)–N(1) 2.145(2), V(1)–N(2) 2.101(2), V(1)–N(3) 2.125(2), O(2)–O(3) 1.424(3), O(2)–V(1)–O(3) 44.3(9); compound **2:** V(1)–O(1) 1.614(2), V(1)–O(2) 1.619(2), V(1)–N(1) 2.100(2), V(1)–N(2) 2.068(2), V(1)–N(3) 2.085(2), O(1)–V(1)–O(2) 111.1(1).

The crystallographic data of complexes **1**, **1′**, **2**, **4**, **5, 6,** and **9** have been collected in Tables S1–S10. ORTEP drawing of **1·**H_2_O (Figure 1A) and **1′** (Figure S2) revealed that the vanadium(V) atom is situated in a seven-coordinate pentagonal-bipyramidal environment with the tridentate pincer ligand pbq^–^, and the peroxido group, in an η^2^-O_2_^2–^ ligation [V(1)–O(2) 1.888(2), V(1)–O(3) 1.888 Å; O(2)–V(1)–O(3) 42.30(9)°], in the equatorial plane and the terminal oxido group and an aqua ligand in the axial positions. The O–O bond length in **1**H_2_O [O(2)–O(3) 1.424(2) Å] is almost in the middle of the range of the O–O distances reported for compounds of the general formula [V^V^(=O)(η^2^-O_2_)(L)] (1.379–1.451 Å, Figure S3). The strong trans effect of the peroxido groups causes a significant lengthening of the V–N_amide_ [V–N_amide_ 2.101(2) Å] bond. The mean *d*(V^V^–N_amide_) reported in the literature is approximately 2.0 Å.^75,76^

The geometric parameters of the calculated optimized structures are close to the experimental results. The deviations of the calculated bond distances from the experimental are small (<2%). The largest deviations are observed for the O–O peroxido bond (1.397 Å), 0.027 Å shorter than the experimental. The calculated V=O, V-(*n*^2^-O_2_), and V–N_amide_ bond lengths are 0.028, 0.030, and 0.023 Å shorter than the experimental values, respectively. The deviations could be attributed to the crystal packing effects in the crystal, which have not been considered in the theoretical calculations.

**Figure 2:**
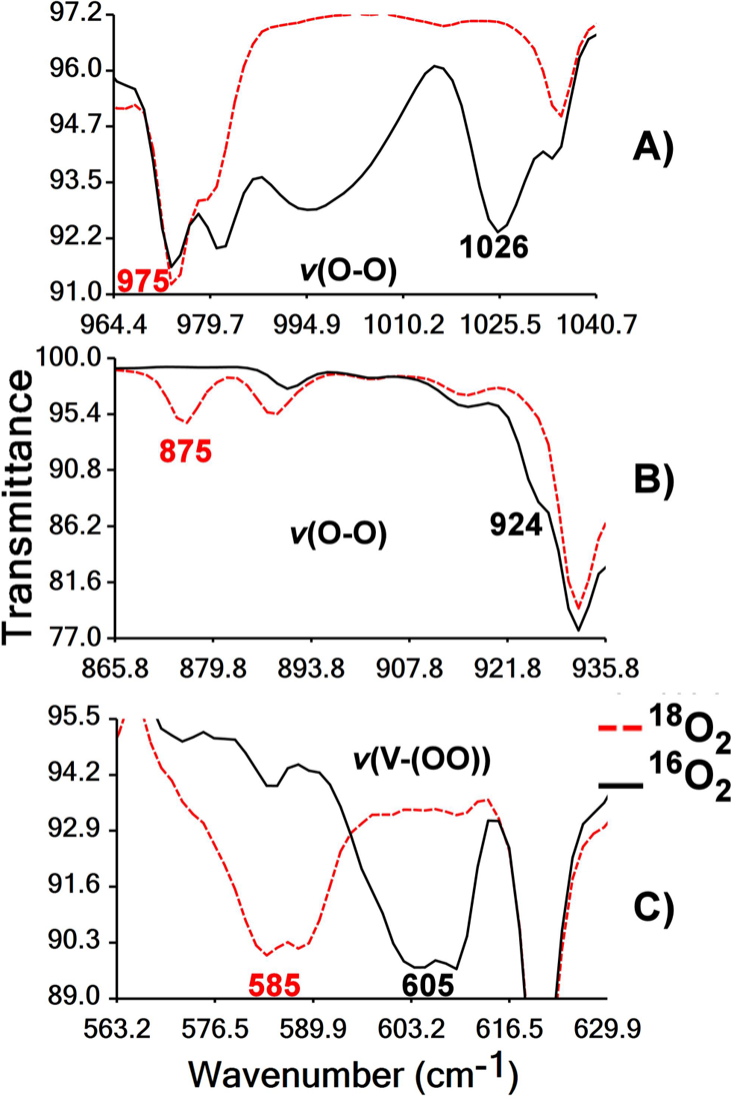
Three different regions of FTIR spectra (ATR) of [V^V^(=O)(η^2^-^16^O_2_)(κ^3^-pbq)(H_2_O)]·H_2_O (**1·**H_2_O), and of its ^18^O_2_-labeled [V^V^(=O)(η^2^-^18^O_2_)(κ^3^-pbq)(H_2_O)]·H_2_O, showing the shift of the O–O and V-(−OO−) stretching bands due to O_2_ labeling. (A) *v*(O–O) stretching, (B) combined *v*(O–O) stretching and pyridine ring breathing, and C) V-(−OO−) stretching bands.

The X-ray structure of **2** (Figure 2B) revealed a mononuclear vanadium(V) compound in a distorted square pyramidal geometry [τ = 0.37, whereas τ = (*b –a*)/60, *b* = N(1)–V(1)–N(3), and *a* = N(2)–V(1)–O(1) angles; τ = 1 for a perfectly trigonal bipyramidal (*D*_3h_) geometry; and τ = 0 for a perfectly square pyramidal (*C*_4v_) structure].^77−79^ The metal ion lies exactly on the equatorial plane defined by the two oxido groups O(1) and O(2) and the amide nitrogen atom N(2). The axial positions are occupied by the pyridine and quinolone nitrogen atoms N(1) and N(3), respectively. The two strong V^V^=O bonds of 1.621(2) Å are typical of the *cis*-[V^V^(=O)_2_]^+^ compounds and are comparable tο those reported in the literature for such compounds.^80^

Complexes **4**, **5,** and **6** have similar to **2** distorted square pyramidal geometries and τ values 0.48, 0.41, and 0.47, respectively. The *d*(V^V^–N_amide_) of *cis*-[V^V^(=O)_2_(L^3,4^)] vanadium complexes with 2-aminomethylpyridine amide ligands pyic^–^, and pyc^–^ ligands in **4** [2.021(2) Å] and **5** [2.023(2) Å], respectively, are significantly shorter than the *cis*-[V^V^(=O)_2_(L^1,6^)] vanadium complexes with 8-aminoquinoline amide ligands pbq^–^, and qqc^–^ ligands in **2** [2.071(2) Å] and **6** [2.064(2) Å], respectively. This difference has been attributed to the larger flexibility of the ligands pyic^–^ and pyc^–^ compared with the rigid pbq^–^, qqc^–^ ones. Ligands pbq^–^ and qqc^–^ can acquire low-energy conformations in their complexes allowing the closer approach of the deprotonated amide nitrogen atom to vanadium nucleus. The deviation of the theoretically calculated bond distances of the optimized structures from the experimental ones is less than 0.005 Å. In addition, the DFT calculations for complexes **2**, **4**, **5,** and **6** revealed that their τ values range from 0.68 to 0.70, which are larger than the experimental ones (0.37–0.47).

Complexes **2** (Figure 2B), **4**, **5**, and **6** (Figures S4–S6) constitute the first examples of the *cis*-[V^V^(=O)_2_]^+^ species containing a V^V^–N_amide_ bond characterized by X-ray crystallography.

The structures of binuclear complexes **7** and **8** were optimized by DFT calculations (Figure S7). In general, the bond lengths and angles of the optimized structures are close to those found in the incomplete crystal structures. The *d*(V–N_amide_) is shorter for V^IV^ ions [V–N_amide_ 2.017 and 2.008 Å in **7** and **8,** respectively] than for V^V^ atom[V–N_amide_ 2.057 Å]. The coordination environment of the V^IV^ is octahedral in both complexes with the metal ion being 0.431 and 0.260 Å above the equatorial plane, defined by the three nitrogen donor atoms of qqc^–^ and the oxygen donor atom of η^2^:η^2^:μ-SO_4_^2–^ in **7** and of a water molecule in **8**, respectively. The larger distortion of **7** than **8** from the octahedral geometry may attributed to the four membered chelating ring of the bridging η^2^:η^2^:μ-SO_4_^2**-**^_._ The two vanadium metal atoms in **8** are bridged through an oxygen atom V^V^=O–V^IV^ with bond distances *d*(V^V^=O) = 1.647 Å and *d*(V^IV^–O) = 2.207 Å. The valences of the metal ions are localized as supported and by the DFT calculations.^81^

### IR and Resonance Raman Spectroscopies

The IR spectra spectra of **7** and **1** are shown in Figures S8 and S9. Full assignments of the peaks of **1**·H_2_O in IR (solid state) and RR (MeOH:H_2_O 90:10 v/v solution) spectra based on the theoretical calculations are collected in Table S11.

Complex **7** gave the characteristic ν(V=O) stretching vibration at 982 cm^–1^. The bridging chelate SO_4_^2–^ gives three strong peaks at 1151, 1035, and 950 cm^–1^ assigned to the splitting of the fundamental vibration ν_3_ due to the reduction of the symmetry of the SO_4_^2–^ from a *T*_d_ in the free anion to *C*_2v_ in the complex.^82^ DFT calculated frequencies multiplied with a scaled factor 0.945 match those found from the spectrum.^83^

The scaled by a factor of 0.945^83^ calculated wavenumbers of the vibrations of **1** are in close agreement with the experimental values, taking into account that the theoretical calculations refer to water solvent, while the experimental values refer to the solid state. The largest difference is observed for the V=O stretching vibration, 965 vs 1037 cm^–1^ for the experimental and calculated values, respectively. This difference might be attributed to the shorter V=O bond length of the theoretically optimized structure (1.566 Å) than the experimental one [1.599 (2) Å].

Complexes [V^V^(=O)(η^2^-^16^O_2_)(κ^3^-pbq)(H_2_O)]·H_2_O (**1·**H_2_O) and its ^18^O_2_-labeled [V^V^(=O)(η^2^-^18^O_2_)(κ^3^-pbq)(H_2_O)]·H_2_O analogue were characterized by FTIR spectroscopy. Oxidoperoxidovanadium(V) complexes are known to have strong, distinct V^V^=O and O–O IR stretches, and assignments of these stretches for [V^V^(=O)(η^2^-O_2_)]^+^ were confirmed by ^18^O_2_ labeling experiments. The FTIR spectra (KBr) of [V^V^(=O)(η^2^-^16^O_2_)(κ^3^-pbq)(H_2_O)]·H_2_O (**1·**H_2_O) and of ^18^O_2_ labeled [V^V^(=O)(η^2^-^18^O_2_)(κ^3^-pbq)(H_2_O)]·H_2_O are shown in Figure S9A. The strong peaks in the regions 1400–1600 and 930–980 cm^–1^ have been assigned mainly to the stretching vibrations of the amidic ligand and the V^V^=O bond, respectively. The peaks associated either with the ligand or the group V^V^=O of [V^V^(=O)(η^2^-^16^O_2_)(κ^3^-pbq)(H_2_O)]·H_2_O (**1·**H_2_O) and of ^18^O_2_-labeled [V^V^(=O)(η^2^-^18^O_2_)(κ^3^-pbq)(H_2_O)]·H_2_O remain the same (Figure S9A).

Isotopic labeling studies support the presence of a peroxido ligand coordinated to the V^V^ in **1·**H_2_O. More specifically, the ^16^O complex (**1·**H_2_O) shows bands at 1026 (Figure 2A) and 605 cm^–1^ (Figure 2C) which were assigned to a ν(^16^O–^16^O) and ν(^16^O–V^V^) stretches, respectively. In the ^18^O-isotopomer these bands are shifted to 975 (Figure 2A) and 585 cm^–1^ (Figure 2C), respectively. The observed vibrational difference between the two isotopomers is in excellent agreement with the harmonic O–O oscillator [ν(^16^O_2_)/ν(^18^O_2_)] 1.06; calcd 1.05).^84^ In addition, the 924 cm^–1^ band of ^16^O complex that appears as a shoulder to the strong V^V^=O bands, between 930 and 960 cm^–1^, shifts to 875 cm^–1^ (Figure 2B) for the ^18^O-isotopomer, and is assigned to a combined O–O stretch and pyridine ring breathing.

The calculated frequencies scaled by a factor of 0.945^83^ gave wave numbers at 1024 [ν(^16^O–^16^O)], 936 [combination of ν(V=^16^O) and ν(^16^O–^16^O) vibrations], and 595 and 580 cm^–1^ [ν(^16^O–V^V^)], which are compared very well with the experimental values.

Resonance Raman (RR) spectra of the CH_2_Cl_2_ solution of [V^V^(=O)(η^2^-^16^O_2_)(κ^3^-pbq)(H_2_O)]·H_2_O and V^V^(=O)(η^2^-^18^O_2_)(κ^3^-pbq)(H_2_O)]·H_2_O with an excitation at 368.9 nm are shown in Figure S9B. The spectra are dominated mainly from the peaks of the ligand, whereas the peaks originated from the peroxido groups and V=O are weak (for a detailed discussion see ESI, Figure S9B).

### NMR Spectroscopy

The ^1^H and ^51^V NMR chemical shifts in solution (CD_3_OD) of the vanadium(V) complexes of the general formulas [V^V^(=O)(η^2^-O_2_)(κ^3^-L^1–4^)(Solv)] and *cis*-[V^V^(=O)_2_(κ^3^-L^1–6^)] are given in Tables S12 and S13.

The ^51^V NMR chemical shifts range from −604 to −645 for the former and from −495 to −507 ppm for the latter complexes, respectively. For example, complexes [V^V^(=O)(η^2^-O_2_)(κ^3^-pbq-*N*_*q*_*,N*_*am*_*,N*_*py*_)(CH_3_OH)] (**1′**) and *cis*-[V^V^(=O)_2_(κ^3^-pbq-*N*_*q*_*,N*_*am*_*,N*_*py*_)] (**2**) gave peaks at −640 and −506 ppm, respectively. These values are close to the expected for monoperoxido and five-coordinate dioxidovanadium(V) complexes with N donor atoms.

The ^1^H NMR chemical shifts of the ligands HL^1–4^ in both [V^V^(=O)(η^2^-O_2_)(κ^3^-L^1–4^)(Solv)] and *cis*-[V^V^(=O)_2_(κ^3^-L^1–4^)] complexes are significantly shifted to lower field in comparison with the chemical shifts of the free ligands (HL^1–4^), providing evidence that the vanadium(V) compounds retain their integrity in solution. The [V^V^(=O)(η^2^-O_2_)(κ^3^-L^1–4^)(Solv)] complexes exhibit larger low-field ^1^H NMR shifts than the *cis*-[V^V^(=O)_2_(κ^3^-L^1–4^)] compounds. A large shift was obtained for the pyridine/quinolone protons in ortho- and para- positions suggesting ligation of the pyridine/quinolone nitrogen atoms to the metal ion. For example, for **1′**, the shifts of H(1) and H(10) (Scheme 3) upon ligation of pbq^–^ to V^V^ ion were 1.01 and 1.16 ppm, respectively. H(5) (Scheme 3) shows a significant shift to lower field for both **1’** (Δδ = 0.39 ppm) and **2** (Δδ = 0.21 ppm), compared to the free ligand, suggesting coordination from the deprotonated amide nitrogen of pbq^–^.

### UV–Vis Spectroscopy

The UV–vis spectra of the [V^V^(=O)(η^2^-O_2_)(κ^3^-L^1–4^)(Solv)] and *cis*-[V^V^(=O)_2_(κ^3^-L^1–6^)] complexes are shown in Figures S10–S15. The MeOH solutions of the free ligands absorb at wavelengths below 320 nm, while compound **3,** [V^V^(=O)(η^2^-O_2_)(κ^3^-pyic-*N*_*py*_*,N*_*am*_*,N*_*q*_)(H_2_O)], shows a ∼30 nm redshift in comparison with the free ligands. The spectra of [V^V^(=O)(η^2^-O_2_)(κ^3^-L^1–4^)(Solv)] compounds gave broad bands arising from the O–O^2–^ to vanadium(V) atom charge transfer in the region of 330–450 nm (ε ∼ 2000 M^–1^ cm^–1^). The energy of the peaks depends on the aromatic system of the ligand. The vanadium compounds with ligands containing large π-aromatic systems absorb in lower energy than the compounds with the smaller π-delocalized systems. For example, the replacement of the 2-aminomethylpyridine of the ligand Hpyic, with 8-aminoquinoline (Hpyc) causes a redshift of 80 nm.

### EPR Spectroscopy

The X-band cw-EPR spectrum of a frozen (120 K) glacial MeOH solution of the dinuclear V^IV^_2_ complex **7** gave peaks with parameters *g*_⊥_= 1.976, *g*_∥_ = 1.942, *A*_⊥_ = 59.20 × 10^–4^ cm^–1^, *A*_∥_ = 165.1 × 10^–4^ cm^–1^, and isotropic Lorentzian line shape broadening (lwpp) 0.95 mT_._ The sharp discriminated peaks of **7** suggest that there is not any interaction between the two paramagnetic metal centers, thus **7** in solution breaks down to monomers [V^IV^(=O)(κ^3^-qqc)(MeOH)_2_]^+^. The calculated *A*_∥_ value of 165.1 × 10^–4^ cm^–1^ for [V^IV^(=O)(κ^3^-qqc)(MeOH)_2_]^+^, using the additivity rule, is similar to the experimental value of *A*_∥_. The value of 39 × 10^–4^ cm^–1^ has been used for the deprotonated amide nitrogen.^85−87^ The DFT calculated value of *A*_∥_ for complexes [V^IV^(=O)(κ^3^-pbq)(MeOH)_2_]^+^ and **8** were 166.0 and 165.3, respectively, which are very close to the experimental values.^88−90^ The calculated *A*_*x*_ and *A*_*y*_ values being 62.5 and 61.2 cm^–1^, respectively, also agree with the experimental ones and support the tetragonal symmetry for [V^IV^(=O)(κ^3^-qqc)(MeOH)_2_]^+^.

### Cyclic Voltammetry

The CV data and cyclic voltammograms of Hpbq, [V^V^(=O)(η^2^-O_2_)(κ^3^-pbq)(H_2_O)]·H_2_O (**1**·H_2_O), and *cis*-[V^V^(=O)_2_(κ^3^-pbq)] (**2**) in solution (CH_3_CN and CH_2_Cl_2_) are shown in Table S14 and Figure S16, respectively. The CVs of Hpbq and **2** show only the waves from the oxidation and reduction of the ligand at ∼1.78 and ∼−0.90 V vs NHE, respectively, while complex **1**·H_2_O shows an additional peak at 1.63 V vs NHE assigned to the 2e^–^ oxidation of η^2^-O_2_^2–^ to O_2_ (eq 5).
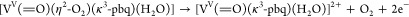
5

Theoretical calculations confirm the above oxidation reactions. *E*^0^ has been calculated by theory to be 1.756 V vs NHE^91^ in light with the respective experimentally derived *E*^0^ being 1.63 V. The CVs of **1**·H_2_O in solution (CH_3_CN) at various scan rates (Figure S17) show, at high scan rates, the appearance of two new cathodic peaks at ∼1.04 and −0.05 V, associated with the peak at 1.63 V, and were assigned to the one-electron reductions of [V^V^O(η^2^-O_2_)(κ^3^-pbq)(H_2_O)]^2+^ to [V^V^(=O)(η^2^-O_2_)(κ^3^-pbq)(H_2_O)]^·^^+^ and of [V^V^(=O)(η^2^-O_2_)(κ^3^-pbq)(H_2_O)]^·+^ to [V^V^(=O)(η^2^-O_2_)(κ^3^-pbq)(H_2_O)], respectively.

#### Characterization of the Intermediate (**Id**)

##### Experiments Typically Were Performed in H_2_O:CH_3_OH (25:75, v/v) Solutions Unless Otherwise Stated

The intermediate was generated following the procedure: Complex [V^IV^(=O)(κ^3^-pbq)(H_2_O)_2_]^+^ was synthesized in situ by heating under argon for 10 min a solution of V^IV^OSO_4_·3.5H_2_O/Hpbq in equimolar ratio. Then, O_2_ was bubbled to the solution for 5 min followed by the deoxygenation of it with argon for 5 min. Upon addition of O_2_ to the solution, its color changed from yellow-brown to red. The [V^IV^(=O)(κ^3^-pbq)(H_2_O)_2_]^+^ gave the most stable intermediate (**Id**) of all the amidate ligands and was characterized by cw-EPR, ^1^H NMR spectroscopies, and electrochemistry.

### EPR Spectroscopy

**Figure 3:**
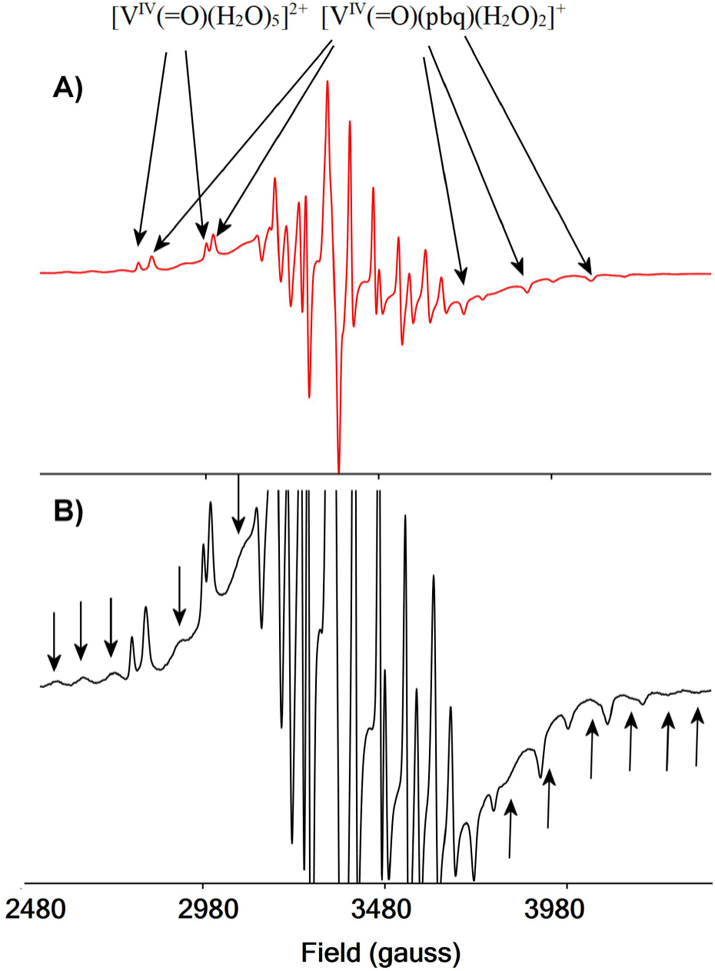
(A) X-band cw-EPR spectrum of V^IV^(=O)^2+^-Hpbq-O_2_ system in a frozen (120 K) solution. (B) Expansion of (A) to show better the peaks of intermediate (**Id**) with arrows.

The X-band EPR spectrum of V^IV^(=O)^2+^-Hpbq in solution, prior to the bubbling of O_2_ to it, revealed the presence of two species, namely: [V^IV^(=O)(κ^3^-pbq)(H_2_O)_2_]^+^ (∼70% of the total vanadium) and [V^IV^(=O)(H_2_O)_5_]^2+^ (30%) (Figure 3). Upon addition of dioxygen to the above solution an additional signal with broad peaks appeared, which resembles the cw-EPR spectra of two interacting vanadium hyperfine coupled spins.^92,93^ The EPR parameters of [V^IV^(=O)(κ^3^-pbq)(H_2_O)_2_]^+^_(_*g*_⊥_= 1.975, *g*_∥_ = 1.943, *A*_⊥_ = 59.60 × 10^–4^ cm^–1^, *A*_∥_ = 165.7 × 10^–4^ cm^–1^, and lwpp = 1.01 mT) are similar to the parameters found for [V^IV^(=O)(κ^3^-qqc)(H_2_O)_2_]^+^, as expected due to the same coordination environment around V^IV^ ion for both complexes. The parameters for [V^IV^(=O)(H_2_O)_5_]^2+^ were found *g*_⊥_= 1.972, *g*_∥_ = 1.930, *A*_⊥_ = 69.03 × 10^–4^ cm^–1^, *A*_∥_ = 180.9 × 10^–4^ cm^–1^, and lwpp = 0.72 mT. The broad signal appears only after the addition of O_2_ in the solution, and thus, it is assigned to the intermediate species (**Id**). The possibility of this signal to come from species such as [{V^IV^(=O)(κ^3^-pbq)}_2_(η^2^:η^2^_:_μ-SO_4_)] is ruled out because the cw-EPR spectra of the solutions under N_2_ do not give the broad signal. In addition, vanadium complexes with the nonbridging Cl^–^ counteranion instead of SO_4_^2–^ show the same broad signal under O_2_. Theoretical calculations revealed two possible intermediates the mononuclear radical [V^V^(=O)(η^2^-O_2_)(κ^3^-pbq)]^·+^ and the binuclear [V^IV^(=O)(κ^3^-pbq)(H_2_O)(η^1^,η^1^-O_2_)V^IV^(=O)(κ^3^-pbq)(H_2_O)]^2+^. Based on the peaks at the two edges and the half-field forbidden Δ*M*_s_ = 2 peaks of the spectrum and considering two metal-centered spins, the broad signal was simulated (Figure S18). The signal is also similar to the spectra reported in the literature for molecules containing two distant interacting V^IV^ spins.^92,93^

Surprisingly, the EPR spectra of the reaction mixtures of [V^IV^(=O)(κ^3^-L^2–4^)(H_2_O)_2_]^+^ with O_2_ show only signals from [V^IV^(=O)(H_2_O)_5_]^2+^ and [V^IV^(=O)(κ^3^-L^2–4^)(H_2_O)_2_]^+^. Apparently, the intermediates of these reactions are EPR silent, in agreement with the compounds previously reported in the literature.^57,58,61^ Based on the theoretical calculations this was attributed to the antiferromagnetic coupling between the spins of the two V^IV^ atoms in the binuclear [V^IV^(=O)(κ^3^- L^2–4^)(H_2_O)(η^1^,η^1^-O_2_)V^IV^(=O)(κ^3^-L^2–4^)(H_2_O)]^2+^ intermediate.

Trap EPR experiments with DMPO (5,5-Dimethyl-1-pyrroline *N*-oxide) and PNB (*N*-*tert*-Butyl-α-phenylnitrone) do not reveal any new signals agreeing with the lack of radicals in solution and the formation of [V^IV^(=O)(κ^3^-L^1–4^)(H_2_O)(η^1^,η^1^-O_2_)V^IV^(=O)(κ^3^-L^1–4^)(H_2_O)]^2+^ intermediate.

### Electrospray Ionization-Mass Spectrometry

In an effort to investigate the interaction of the vanadium complex [V^IV^(=O)(κ^3^- pbq)(H_2_O)_2_]^+^ with molecular dioxygen we monitored the reaction mixture as a function of the time using electrospray ionization-mass spectrometry (ESI-MS)^94−99^ to identify potentially species generated upon the formation of [V^IV^(=O)(κ^3^-pbq)(H_2_O)_2_]^+^ in solution. Potential identification of intermediate species could provide additional information in regard to the mechanistic aspects and operation mode of [V^IV^(=O)(κ^3^-pbq)(H_2_O)_2_]^+^ in catalytic reactions.

The ESI-MS studies were performed directly on the reaction mixture in positive ionization mode. It was observed that the identified species in the reaction mixture formed instantly upon mixing an aqueous solution of V^IV^(=O)SO_4_·3.5H_2_O (3.5 mg in 0.25 mL H_2_O) and a methanolic solution of the ligand Hpbq (3.9 mg in 0.75 mL CH_3_OH). Monitoring of the reaction mixture revealed that the intensity of observed species in solution increased as a function of the time, indicative of their increased relative concentration in solution. No other transformation or generation of new species were observed during this time. Figure S19 depicts the mass spectrum of the reaction mixture after 2 h. The observation of the singly charged distribution envelopes centered at 332.05 and 347.01 *m*/*z* can be assigned to the [V^V^(=O)(pbq)(OH)]^+^ and [V^V^(=O)(pbq)(O_2_)]^•+^ (where pbq = C_15_H_10_N_3_O), respectively, and correspond to complex **1** resulting from the removal a water molecule and interestingly, interaction with molecular oxygen in the form of peroxo species, respectively. Moreover, at higher *m*/*z* values, the observed isotopic envelopes centered at 646.04, 698.05, and 716.06 *m*/*z*, with formulas [V^IV^(=O)(pbq)(O)V^V^(=O)(pbq)]^+^, [V^IV^(=O)(pbq)(H_2_O)(O_2_)V^V^(=O)(pbq)(H_2_O)]^+^, and [V^IV^(=O)(pbq)(H_2_O)_2_(O_2_)V^(=O)V^O(pbq)(H_2_O)]^+^, respectively, that correspond to oxo-bridged (646.04 *m*/*z*) and peroxo-bridged (698.05 and 716.06 *m*/*z*) dimeric species. Finally, it is quite common for the in situ alteration of the metal’s oxidation state during the course of the ion transfer and has been reported frequently in the literature.^95,99,100^

### ^1^H NMR Spectroscopy

It is worth noting that the different behavior observed in EPR between the V^IV^O^2+^-L^1^ and V^IV^O^2+^-L^2–4^ complexes after oxygenation is also observed in ^1^H NMR spectroscopy. The ^1^H NMR spectra of V^IV^O^2+^ solutions with the ligands L^2–4^ after oxygenation show a decrease of the intensity of the peaks, due to the ligation to V^IV^O_2_^2+^, and the appearance of a broad peak with a line width of ∼2.5 ppm. The cations [V^IV^(=O)(κ^3^-L^2–4^)(H_2_O)_2_]^+^ do not give any signal in the ^1^H NMR spectra; thus, the broad signal is assigned to the intermediate. In addition, the fact that the ^1^H NMR spectra of [V^IV^(=O)(κ^3^-L^5,6^)(H_2_O)_2_]^+^ after oxygenation do not give the broad signal support the above assignment. The emergence of a broad ^1^H NMR signal agrees with a binuclear V^IV^ intermediate with the spins to be antiferromagnetically coupled.^101^

**Figure 4:**
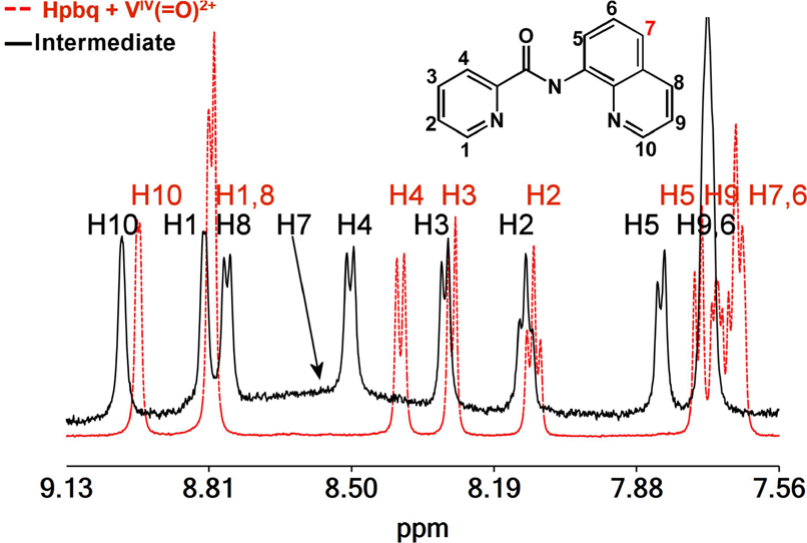
^1^H NMR spectra of [V^IV^(=O)(κ^3^-pbq)(H_2_O)_2_]^+^ in solution prior to and after the oxygenation of it.

The ^1^H NMR spectrum of [V^IV^(=O)(κ^3^-pbq)(H_2_O)_2_]^+^ complex, prior to the oxygenation, is dominated from the peaks of free Hpbq (Figure 4), while upon oxygenation, in contrast to V^IV^O^2+^-L^2–4^ complexes, the peaks are broadened and shifted and assigned to the paramagnetic intermediate radical **Id** (Figure 4). The signal of H7 (Figure 4) of the bound pbq^–^ appeared as a very broad peak at ∼8.5 ppm with a line width of ∼1.5 ppm (Figure 4). The large broadening of proton H7 (Figure 4) is in conformity with the theoretical calculations that reveal the formation of a stable intermediate (**Id**) with partial of the electron spin density delocalized on the pbq^–^ ligand (*vide infra*). The ^1^H NMR spectra of the intermediate calculated by the GIAO/PBE0/Def2-TZVP(V)∪6-31+G(d)(E)/PCM computational protocol in aqueous solution showed the same pattern with the experimentally obtained ^1^H NMR spectra, e.g., the peaks of the protons span from 7.90 to 9.05 ppm, with the peak of the H7 shifted ∼1.1 ppm to lower field in line with the experimental ^1^H NMR findings.

### Electrochemistry

**Figure 5:**
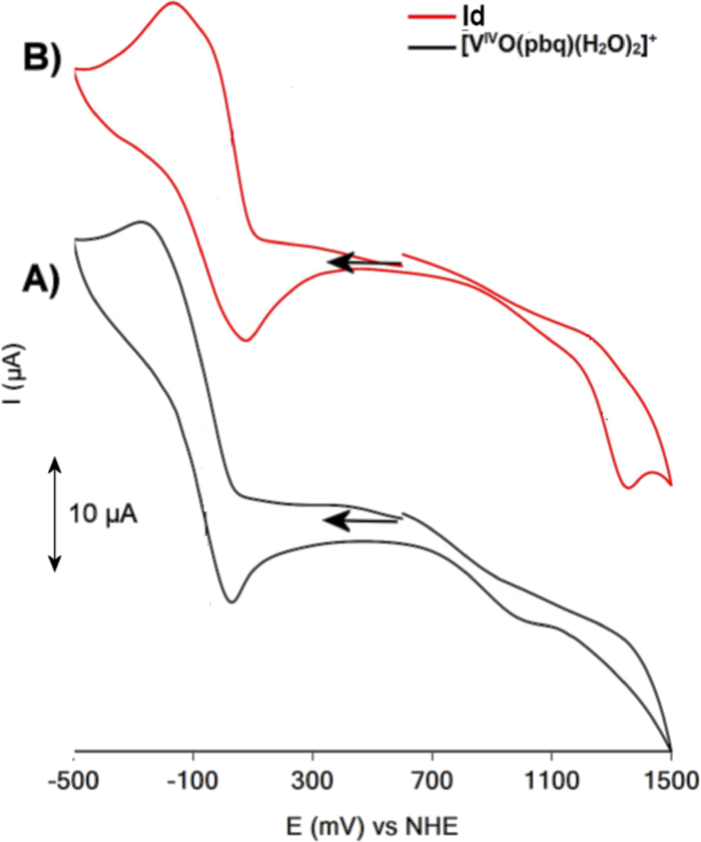
Cyclic voltammograms of [V^IV^(=O)(κ^3^-pbq)(H_2_O)]^+^ (A) and intermediate (**Id**) (B) in solution (MeOH:H_2_O 9:1 v/v), in which excess of O_2_ has been removed by bubbling with Ar. Scan rate was 100 mV/s, and supporting electrolyte is 0.1 M But_4_NBF_4_. Glassy carbon as the working electrode, a platinum wire as the auxiliary electrode, and Ag/AgCl (0.20 V vs NHE) as a reference.

The CV of [V^IV^(=O)(κ^3^-pbq)(H_2_O)_2_]^+^ in solution [H_2_O:CH_3_OH (10:90, v/v)] is shown in Figure 5A and gave an irreversible redox couple at −0.25 V vs NHE (Δ*E* = 250 mV) assigned to the one-electron reduction of [V^IV^(=O)(κ^3^-pbq)(H_2_O)_2_]^+^ centered on the ligand. The CV of the intermediate (**Id**) revealed an anodic peak at 1.20 V (eq 6) and two irreversible redox couples centered at −0.035 (eq 7) and −0.15 V (eq 8) vs NHE (Δ*E* = 250 mV), which were assigned to the oxidation and reductions of intermediate respectively, based on the intermediate suggested from cw-EPR and ^1^H NMR spectra.
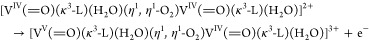
6
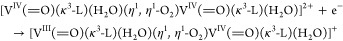
7
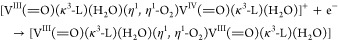
8

The wide voltage range (∼1.3 V) between the oxidation and reduction of the intermediate agrees with the large thermodynamic stability of the radical. The two reduction waves of the intermediate at a similar potential with the mononuclear [V^IV^(=O)(κ^3^-pbq)(H_2_O)_2_]^+^ species are consistent with a binuclear [V^IV^(=O)(κ^3^-L)(H_2_O)(η^1^,η^1^-O_2_)V^IV^(=O)(κ^3^-L)(H_2_O)]^2+^ intermediate. Theoretical calculations confirm the above reactions.

#### Reactivity of [V^IV^(=O)(κ^3^-L^1–6^)(H_2_O)_2_]^+^ toward O_2_ Reductive Activation

##### EPR Spectroscopy

The interaction of dioxygen with [V^IV^(=O)(H_2_O)_5_]^2+^/HL^1-6^ in frozen (120 K) solution was monitored by the X-band cw-EPR spectroscopy vs time (Figure S20). At 0 s, the spectrum is dominated by the peaks of [V^IV^(=O)(H_2_O)_5_]^2+^ and of [V^IV^(=O)(κ^3^-pbq)(H_2_O)_2_]^+^ species. The complex [V^IV^(=O)(κ^3^-pbq)(H_2_O)_2_]^+^ reacts quickly with O_2_ to give the intermediate **Id**. 60 min after the initiation of the reaction, the [V^IV^(=O)(H_2_O)_5_]^2+^ species has been consumed completely, whereas EPR spectra show peaks from [V^IV^(=O)(κ^3^-pbq)(H_2_O)_2_]^+^ and the intermediate. During the formation of [V^V^(=O)(η^2^-O_2_)(κ^3^-pbq)(H_2_O)], the ratio of the concentrations of [V^IV^(=O)(κ^3^-pbq)(H_2_O)_2_^+^]\[intermediate] remains constant.

The cw X-band EPR spectra of the reaction of V^IV^O^2+^ with Hpp, Hpyc, or Hpyic after addition of O_2_ vs time show peaks from the formation of complexes [V^IV^(=O)(κ^3^-L^2–4^)(H_2_O)_2_]^+^ which quickly reacts with O_2_ keeping the quantity of [V^IV^(=O)(κ^3^-L^2–4^)(H_2_O)_2_]^+^ constant, whereas the intensity of the peaks of [V^IV^(=O)(H_2_O)_5_]^2+^ decrease. The cw X-band EPR spectra of the frozen CH_3_OH solutions of V^IV^O^2+^ with Hpic or Hqqc show only the slow formation of the [V^IV^(=O)(κ^3^-pic/qqc)(H_2_O)_2_]^+^ species without the formation of the broad signal (Figure S21).

##### ^1^H NMR Spectroscopy

The ^1^H NMR spectra of the oxidation of [V^IV^(=O)(κ^3^-L^1–6^)(H_2_O)_2_]^+^ by O_2_ vs time in solution are shown in Figures 4 and S22–S24. The ^1^H NMR spectra of the reaction of the system V^IV^O^2+^-Hpbq with O_2_ revealed that the peaks of Hpbq shift and the peak of H(7) collapses. The shift of the peaks of the pbq^–^ vs time might be due to chemical exchange of the free Hpbq with the intermediate. The ^1^H NMR spectra of the reaction of the system V^IV^O^2+^-HL^2–4^ with O_2_ show that all peaks collapse to one broad peak that covers a region from 7.4 to 10 ppm and were assigned to the intermediate [V^IV^(=O)(κ^3^- L^2–4^)(H_2_O)(η^1^,η^1^-O_2_)V^IV^(=O)(κ^3^-L^2–4^)(H_2_O)]^2+^, whereas the only discrete peaks are those of the ligand that is decreasing in intensity due to the complexation with the metal ion (Figure S22). Based on the theoretical studies, this difference is attributed to the limited delocalization of the spin density intermediate on the ligand, for HL^2–4^, compared with the ligand HL^1^. The V^IV^ compounds with the sterically hindered ligands HL^5^ and HL^6^ do not show the formation of any broad peaks (Figures S22), suggesting that the intermediate does not form, in agreement with EPR data accounted for the low reactivity of V^IV^O^2+^-pic^–^/qqc^–^ to reduce O_2_.

The reaction of V^IV^O^2+^-HL_1–4_ with O_2_ takes place in two steps; the first step starts at zero time and ends prior to the formation of [V^V^(=O)(η^2^-O_2_)(κ^3^-L^1–4^)(H_2_O)] and *cis*-[V^V^(=O)_2_(κ^3^-L^1–4^)] (lag time), and the second step begins when the peaks of [V^V^(=O)(η^2^-O_2_)(κ^3^-L^1–4^)(H_2_O)] and *cis*-[V^V^(=O)_2_(κ^3^-L^1–4^)] appear in the spectra. The duration of the former step depends on the reactivity of V^IV^O^2+^-HL^1–4^ toward O_2_, and it ranges from ∼5 min for V^IV^O^2+^-Hpp to 2 h for V^IV^O^2+^(1.9 mM)-Hpbq(1.9 mM). In general, 8-aminoquinoline amidate complexes (V^IV^O^2+^-pbq^–^/pyc^–^) exhibit larger lag times than the 2-aminomethylpyridine amidate complexes (V^IV^O^2+^-pp^–^/pyic^–^), suggesting that the former stabilize the intermediate better than the latter.

##### UV–Vis Spectroscopy

The reductive activation of O_2_ by [V^IV^(=Ο)(κ^3^-L^1–6^)(H_2_O)_2_]^+^ in solution was also monitored by UV–vis spectroscopy. During the experiment the O_2_ concentration was kept constant by continuous bubbling into the solution of air or pure O_2_. The reactions of V^IV^–Hpp and V^IV^–Hpyic with O_2_ were much faster than V^IV^–Hpbq and V^IV^–Hpyc, and it was not possible to separate the two steps by UV–vis spectroscopy. The rates of the reaction of V^IV^–Hpbq and V^IV^–Hpyc with O_2_ were similar.

**Figure 6:**
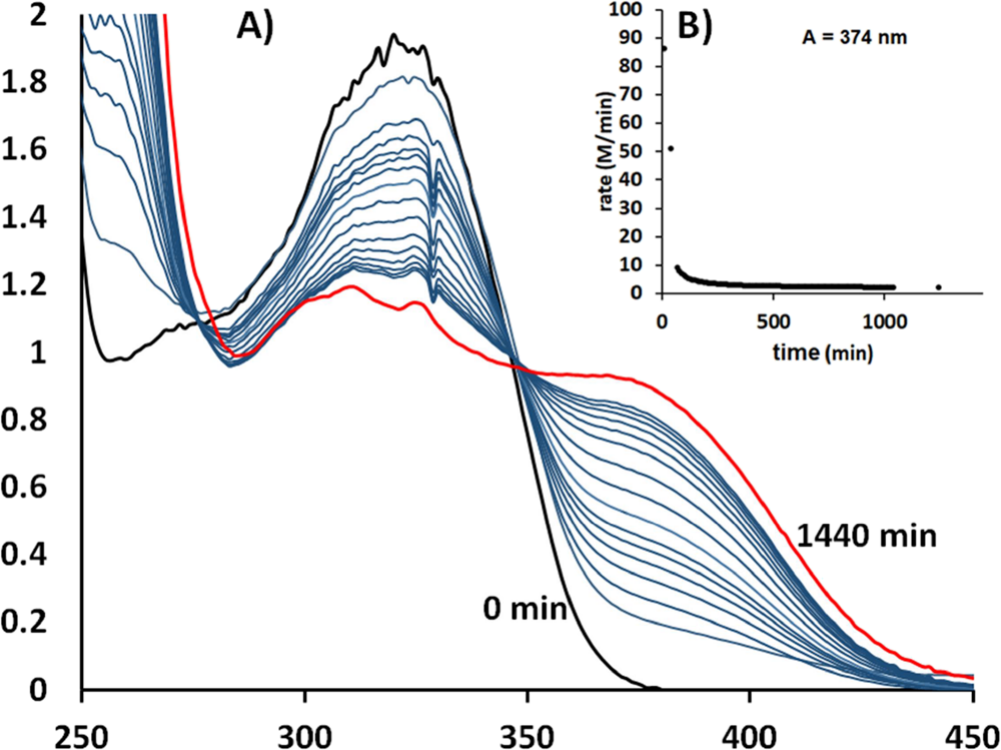
(A) UV–vis spectra of the reaction of equimolar quantities (0.48 mM) of reagents V^IV^OSO_4_·3.5H_2_O and Hpbq in solution with O_2_ vs time (min). The spectra were recorded every 30 min up to 210 min and every 90 min up to 930 min. The concentration of O_2_ in solution was kept constant until the end of the experiment. (B) Graph of the profile of the reaction rate calculated from the absorption at 374 nm vs time and taken from data of graph 4A.

The UV–vis spectra of the reaction of [V^IV^(=O)(κ^3^-pbq)(H_2_O)_2_]^+^ with O_2_ vs time gave two isosbestic points at 280 and 349 nm (Figure 6A). We examined the order of the rate of the reaction with respect to the vanadium species by plotting the rate of the reaction vs time (Figure 6B), and this curve does not fit either to first- or second- order rate toward V^IV^O^2+^, which is indicative for the reaction evolution in more than one steps. This is in line with the results of ^1^H NMR which show two processes taking place, a fast formation of the intermediate and a much slower formation of [V^V^(=O)(η^2^-O_2_)(pbq)(H_2_O)].

The rate law of the formation of the intermediate was calculated from the initial rates at the first 30 min for various concentrations of V^IV^OSO_4_·3.5H_2_O. The logarithmic graph in Figure S25 shows that the reaction rate is a pseudo first order toward V^IV^O^2+^ with *k*_init_ = 0.26 s^–1^.

Moreover, the rate of the formation of the intermediate (first step) increases with the increase of the concentration of V^IV^O^2+^–Hpbq and of O_2_ in solution. The concentration of O_2_ in methanolic solution varies from 1.99 to 10.3 mM,^102,103^ by bubbling air or pure O_2_ into the solution, respectively. The rate of the conversion of intermediate to [V^V^(=O)(η^2^-O_2_)(κ^3^-pbq)(H_2_O)] (second step) remains the same, suggesting that the formation of [V^V^(=O)(η^2^-O_2_)(κ^3^-pbq)(H_2_O)] is dependent only on the concentration of intermediate but not on the concentration of O_2_.

##### ^51^V NMR Spectroscopy

**Figure 7:**
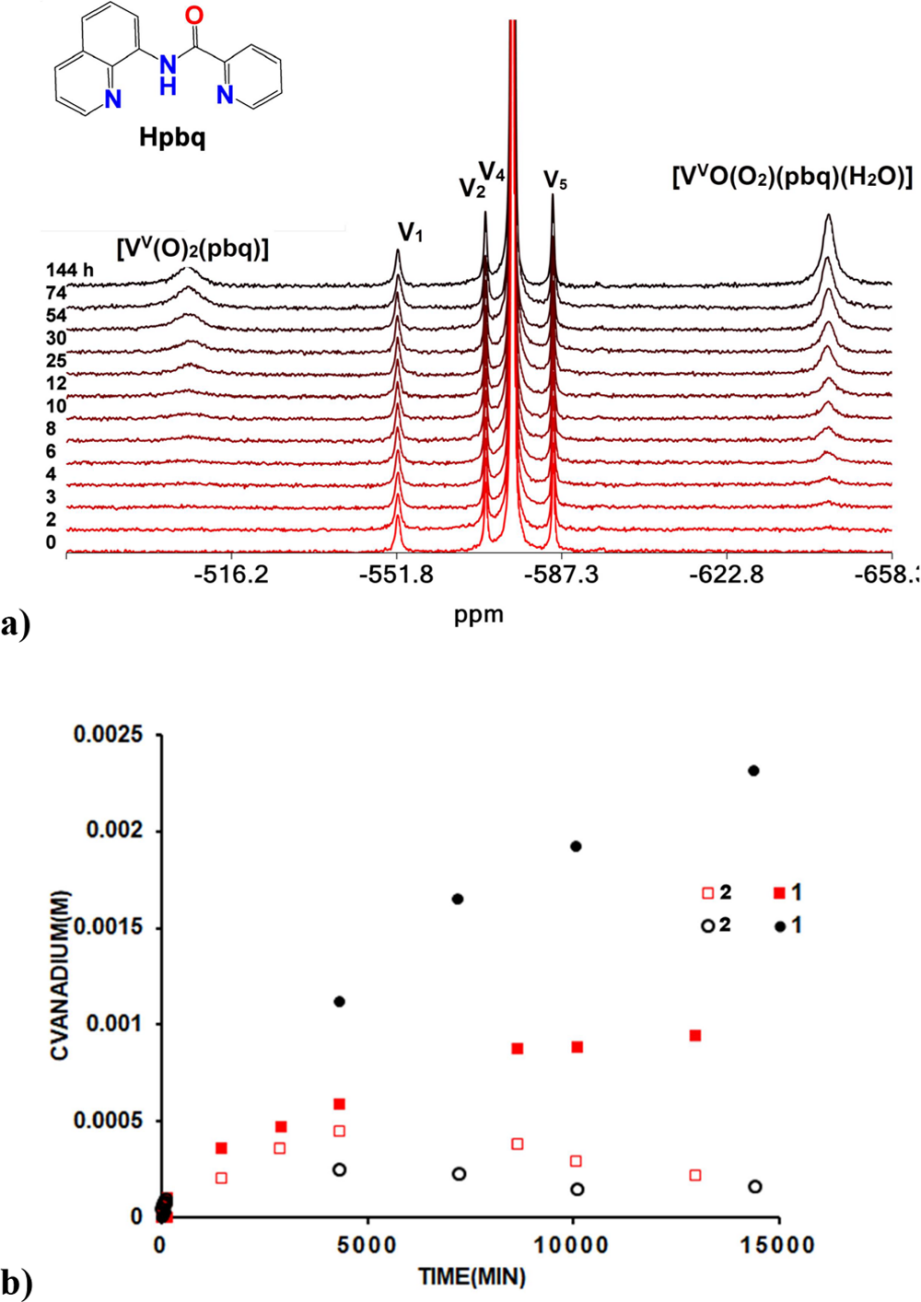
(a) ^51^V NMR spectra of the reaction of V^IV^OSO_4_·3.5H_2_O (0.0144 M)-Hpbq (0.0138 M) in solution with O_2_ vs time (min) and assignments. The V1, V2, V4, and V5 peaks were assigned to monomer, dimer, tetramer, and pentamer vanadate oligomers, respectively, and originated from the external aqueous NaV^V^O_3_ solution used as quantitative standard. (b) Graph showing the concentration of the vanadium(V) compounds [V^V^(=O)(η^2^-O_2_)(κ^3^-pbq)(H_2_O)] and *cis*-[V^V^(=O)_2_(κ^3^-pbq)] vs time in a solution of V^IV^OSO_4_·3.5H_2_O (0.0144 M)-Hpbq (0.0138 M); with hydroquinone (0.0138 M) (black circles), without hydroquinone (red squares).

The ^51^V NMR spectra of the reductive activation of O_2_ by V^IV^O^2+^- HL^1–6^ in solution vs time are shown in Figures 7a, S26, and S27. The quantities of the [V^V^(=O)(η^2^-O_2_)(κ^3^-L^1–4^)(H_2_O)] and *cis*-[V^V^(=O)_2_(κ^3^-L^1–6^)] compounds were calculated by intergrading the signals of the respective peaks below −605 ppm for the former and at −500 ppm for the latter compounds vs the integral of the peaks of an external standard. The concentration of the [V^V^(=O)(η^2^-O_2_)(κ^3^-L^1–4^)(H_2_O)] and *cis*-[V^V^(=O)_2_(κ^3^-L^1–6^)] compounds produced from the oxidation of the respective [V^IV^(=O)(κ^3^-L^1–6^)(H_2_O)_2_]^+^ compounds vs time are depicted in Figures 7b and S28. Oxidation of [V^IV^(=O)(κ^3^-L^5,6^)(H_2_O)_2_]^+^ species by O_2_ produces only the *cis*-[V^V^(=O)_2_(κ^3^-L^5,6^)] complexes. Both *cis*-[V^V^(=O)_2_(κ^3^-L^1–4^)] and [V^V^(=O)(η^2^-O_2_)(κ^3^-L^1–4^)(H_2_O)] compounds are produced only from L^1^ = pbq^–1^, L^2^ = pp^–1^, L^3^ = pyc^–1^, L^4^ = pyic^–1^. The total reactivity of [V^IV^(=O)(κ^3^-L^1–6^)(H_2_O)_2_]^+^ toward O_2_ reduction follows the order Hpp > Hpyic > Hpbq ≥ Hpyc > Hpic > Hqqc. The quantities [V^V^(=O)(η^2^-O_2_)(κ^3^-pbq)(H_2_O)] and [V^V^(=O)(η^2^-O_2_)(κ^3^-pyc)(H_2_O)] are larger than *cis*-[V^V^(=O)_2_(κ^3^-pbq)] and *cis*-[V^V^(=O)_2_(κ^3^-pyc)] respectively. In marked contrast, the quantity of *cis*-[V^V^(=O)_2_(κ^3^-pp/pyic)] is greater, than their peroxide analogues [V^V^(=O)(η^2^-O_2_)(κ^3^-pp/pyic)(H_2_O)].

The ratio of {[V^V^(=O)(η^2^-O_2_)(κ^3^-L^1–4^)(H_2_O)]}:*cis*-[V^V^(=O)_2_(κ^3^-L^1–4^)] from the oxidation of [V^IV^(=O)(κ^3^-L^1–4^)(H_2_O)_2_]^+^ by O_2_ is solvent dependent and the largest quantities of [V^V^(=O)(η^2^-O_2_)(κ^3^-L^1–4^)(H_2_O)] are formed in MeOH, and the ratio [peroxido]/[dioxido] increases with the time (Figure 4b). ^1^H NMR spectroscopy shows that the ligand is not decomposed during the reaction suggesting that methanol is consumed for the reduction of the intermediate. This hypothesis has been confirmed by the addition of hydroquinone, which is a stronger reducing agent than methanol. The addition of hydroquinone inhibits the formation of *cis*-[V^V^(=O)_2_(κ^3^-L^1–4^)], resulting in the formation of remarkably higher quantities of [V^V^(=O)(η^2^-O_2_)(κ^3^-L^1–4^)(H_2_O)]. As shown in Figures 7b and S29, the concentration of [V^V^(=O)(η^2^-O_2_)(κ^3^-pbq)(H_2_O)] is twice higher in the presence of hydroquinone compared with the concentration of it in the absence of hydroquinone. The reactivity of O_2_ reduction to O_2_^2–^ by the [V^IV^O(pbq)(H_2_O)_2_]^+^ is inversely related to the dissociation energies of H^·^ donors, HO-H 118.8 kcal/mol^104^ ≪CH_3_O–H 104.2 kcal/mol,^105^ HOCH_2_–H 96.0 kcal/mol^106,107^ <HOC_6_H_4_O–H 83.4 kcal/mol,^108^ further supporting the above-mentioned mechanism.

The addition of small quantities of H^+^ to the V^IV^O^2+^-(HL^1–4^) solution results in the formation of smaller quantities of [V^V^(=O)(η^2^-O_2_)(κ^3^-L^1–4^)(H_2_O)] (Figure S30), suggesting that protons inhibit the reaction.

##### Comparison of the Reactivity of V^IV^-L^1–4^ with V^IV^-terpy toward O_2_ Reduction

The reactivity of the [V^IV^(=O)(κ^3^-L^1–4^)(H_2_O)_2_]^+^ complexes was compared with the [V^IV^(=O)(κ^3^-terpy)(H_2_O)_2_]^2+^ complexes. Similarly to the amidate ligands, terpy has three nitrogen donor atoms, is planar with a delocalized π bonding system, but it is neutral. ^51^V NMR spectroscopy shows the reaction of a solution of [V^IV^(=O)(κ^3^-terpy)(H_2_O)_2_]^2+^ with O_2_ gives both [V^V^(=O)(η^2^-O_2_)(κ^3^-terpy)(H_2_O)]^+^ and *cis*-[V^V^(=O)_2_(κ^3^-terpy)]^+^; however, the reactivity is far less than the reactivity of [V^IV^(=O)(κ^3^-L^1–4^)(H_2_O)_2_]^+^. Small quantities of [V^V^(=O)(η^2^-O_2_)(κ^3^-terpy)(H_2_O)]^+^ and *cis*-[V^V^(=O)_2_(κ^3^-terpy)]^+^ appear 4 days after the reaction of [V^IV^(=O)(κ^3^-terpy)(H_2_O)_2_]^2+^ with O_2_ in solution.

##### Investigation of the Mechanism of O_2_ Activation by [V^IV^(=O)(κ^3^-L^1–6^)(H_2_O)_2_]^+^ in the Presence of Either Hydroquinone or Triphenylphosphine

**Figure 8:**
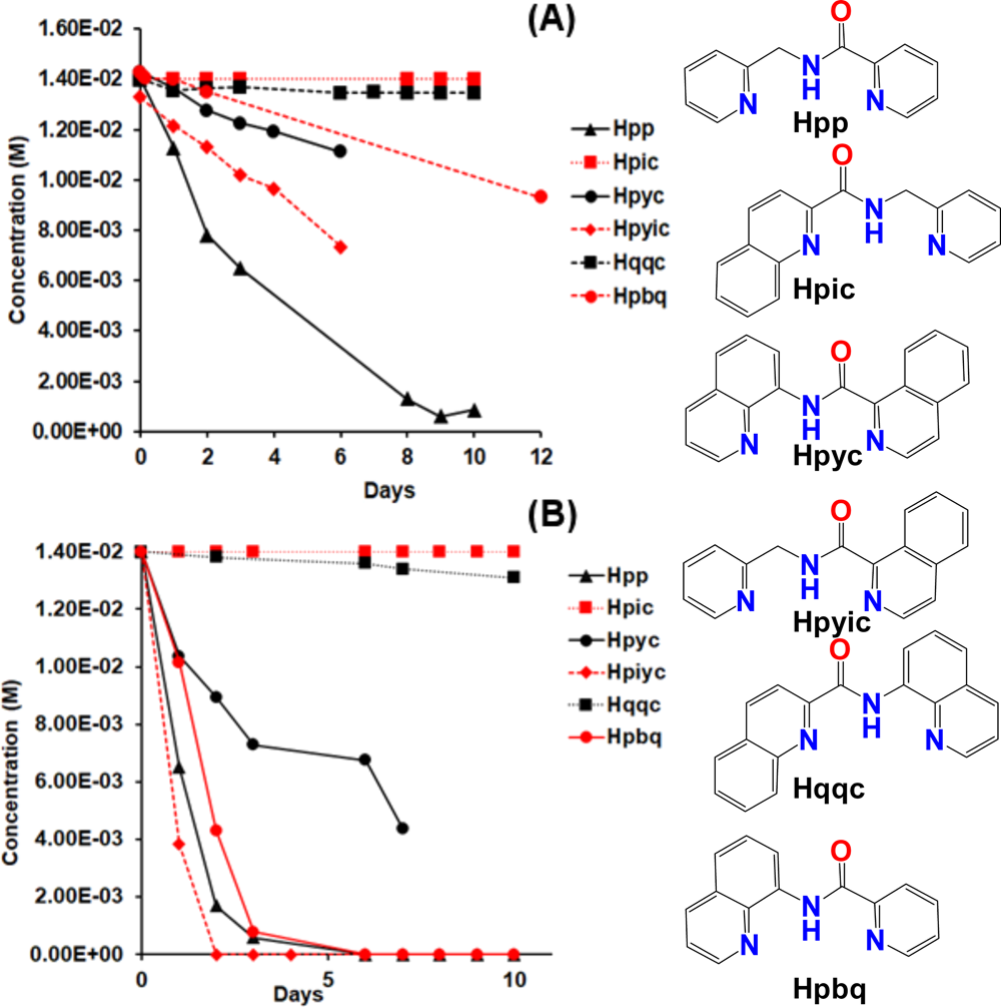
Oxidation of (A) hydroquinone and (B) PPh_3_ in a (10:90, v/v) D_2_O:CD_3_OD solution of V^IV^OSO_4_·3.5H_2_O (0.0144 M)-L^1–6^ (0.0138 M) vs time. The *y* axis shows the concentration of either hydroquinone or PPh_3_ left in solution vs time. (L^1^ = pbq^–1^, L^2^ = pp^–1^, L^3^ = pyc^–1^, L^4^ = pyic^–1^, L^5^ = pic^–1^, L^6^ = qqc^–1^).

**Scheme 6:**
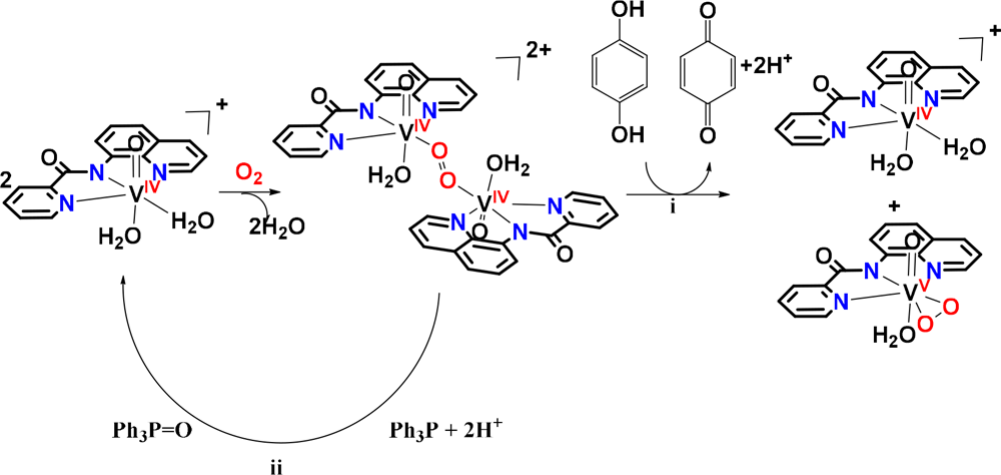
Reactions of [V^IV^(=O)(κ^3^-pbq)(H_2_O)_2_]^+^ with O_2_ in the Presence of Either Hydroquinone (i) or PPh_3_ (ii)

The ^1^H NMR spectra of the VOSO_4_·3.5H_2_O-HL^1–4^-hydroquinone system in solution [D_2_O:CD_3_OD, 10:90, v/v)] show that the two-electron oxidation of hydroquinone is associated with the almost exclusive formation of [V^V^(=O)(η^2^-O_2_)(κ^3^-L^1–4^)(H_2_O)] in agreement with ^51^V spectroscopy (Figure S31). [V^V^(=O)(η^2^-O_2_)(κ^3^-L^1–4^)(H_2_O)] does not oxidize hydroquinone, thus, hydroquinone is oxidized only by the intermediate. More specifically, two equivalents of the intermediate react with one equivalent of hydroquinone leading to the formation of two equivalents of [V^V^(=O)(η^2^-O_2_)(κ^3^-L^1–4^)(H_2_O)] and one equivalent of quinone according to equation i, Scheme 6. The reactivity of the [V^IV^(=O)(κ^3^-L^1–6^)(H_2_O)_2_]^+^ catalysts toward oxidation of hydroquinone follows the order Hpp > Hpyic > Hpyc ≥ Hpbq ≫ Hqqc ∼ Hpic (Figure 8A), which is similar to the tendency of the [V^IV^(=O)(κ^3^-L^1–6^)(H_2_O)_2_]^+^ to reduce O_2_ (*vide supra*).

The ^1^H NMR spectra of the V^IV^OSO_4_·3.5H_2_O-HL^1–6^ with an excess of triphenylphosphine (2- and 20-fold the vanadium concentration) show the full oxidation of triphenylphosphine to triphenylphosphate (Figures S32, S33, and equation ii in Scheme 6). The reactivity of the [V^IV^(=O)(κ^3^-L^1–6^)(H_2_O)_2_]^+^ catalysts toward oxidation of triphenylphosphine follows the order Hpyic ≥ Hpp ≥ Hpbq > Hpyc ≫ Hqqc ∼ Hpic (Figure 6B), which is similar to the tendency of the [V^IV^(=O)(κ^3^-L^1–6^)(H_2_O)_2_]^+^ to reduce O_2_ (*vide supra*). The reaction of O_2_ with [V^IV^(=O)(κ^3^-L^1–4^)(H_2_O)_2_]^+^ in solution and in the presence of small quantities of triphenylphosphine (2- to 3-fold the vanadium concentration) show only the formation of small quantities of [V^V^(=O)(η^2^-O_2_)(κ^3^-L^1–4^)(H_2_O)] (10–20% of the total vanadium), which remain stable during the experiment as it was evident from the ^1^H NMR spectra (Figure S33). At higher quantities of triphenylphosphine, the ^1^H NMR spectra do not show any formation of peroxido V^V^ complexes, and this is attributed to the fact that triphenylphosphine subtracts an oxygen atom directly from the intermediate regenerating [V^IV^(=O)(κ^3^-L^1–4^)(H_2_O)_2_]^+^. In marked contrast to the complexes [V^IV^(=O)(κ^3^-L^1–4^)(H_2_O)_2_]^+^, the cations [V^IV^(=O)(κ^3^-L^5,6^)(H_2_O)_2_]^+^ oxidize triphenylphosphine with O_2_ very slowly (do not show any formation of triphenylphosphate after 12 days).

#### Mechanistic Details of O_2_ Reduction by [V^IV^(=O)(κ^3^-L^1–6^)(OH_2_)_2_]^+^ Probed by DFT Computational Investigations

Two possible pathways toward the activation of O_2_ by the [V^IV^(=O)(κ^3^-L^1–6^)(OH_2_)_2_]^+^ have been investigated by DFT calculations. A mononuclear pathway through the formation a radical [V^V^(=O)(η^2^-O_2_)(κ^3^-L^1–4^)(OH_2_)]^·+^ intermediate and a binuclear pathway through the formation of a binuclear η^1^,η^1^-O_2_ bridged paramagnetic intermediate [V^IV^(=O)(κ^3^-L^1–4^)(H_2_O)(η^1^,η^1^-O_2_)V^IV^(=O)(κ^3^-L^1–4^)(H_2_O)]^2+^.

The optimized geometries of the reactants, intermediates and products involved for both mechanisms with selected structural parameters are shown in Figures S34 and S35. The calculated bond lengths are in line with the experimental ones. The differences of bond lengths between theory and experiment around the coordination spheres of **1**, **2**, **4**, and **6** are within the range 0.001–0.108 Å.

A comparison of the reactivity of the [V^IV^(=O)(κ^3^-L^1–6^)(OH_2_)_2_]^+^ with dioxygen reveals that it is analogous to the distance between H(10) and H(1 or 11) atoms in the six ligands (Scheme 3) (Hpp, 6.126 Å > Hpyic, 5.792 Å > Hpbq, 5.730 > Hpyc 5.412 > Hpic, 5.091 > Hqqc, 4.735). Thus, the most reactive complex is [V^IV^(=O)(κ^3^-pp)(OH_2_)_2_]^+^, while complexes [V^IV^(=O)(κ^3^-pic/qqc)(OH_2_)_2_]^+^ react very slowly with O_2_ forming only the *cis*-[V^V^(=O)_2_(κ^3^-pic/qqc)]. In the optimized distorted octahedral structures of [V^IV^(=O)(κ^3^-L^1–6^)(OH_2_)_2_]^+^, the vanadium atom is out of the basal plane toward the oxido ligand, following a trend pbq^–^, 0.235 Å < pyic^**-**^, 0.277 Å < pp^–^, 0.288 Å < pyc^–^ (0.306 Å) < pic^**-**^ (0.486 Å) < qcc^–^ (0.520 Å), approximately inversely analogous to the distances between H(10) and H(1 or 11).

##### Mononuclear Pathway

**Scheme 7:**
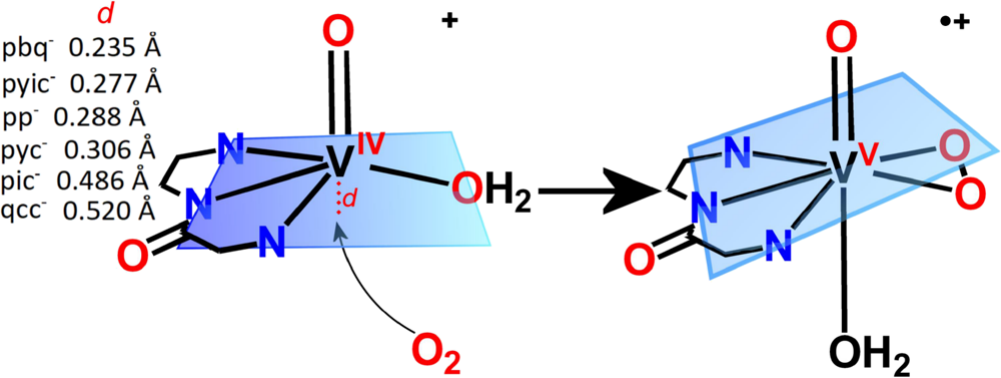
Dioxygen Approach to the [V^IV^(=O)(κ^3^-L^1-6^)(OH_2_)]^+^ Complexes and Formation of the Intermediate Radical

The O_2_-activation proceeds through dissociation of H_2_O in *trans* position to the oxido group of [V^IV^(=O)(κ^3^-L^1–6^)(OH_2_)_2_]^+^ complexes, followed by the coordination of O_2_ to the vanadium(IV) atom of the complexes [V^IV^(=O)(κ^3^-L^1–4^)(OH_2_)]^+^ in a side-on coordination mode, forming the mononuclear [V^V^(=O)(η^2^-O_2_)(κ^3^-L^1–4^)(OH_2_)]^·+^ intermediate (Scheme 7). Calculations performed on starting geometries with an end-on bonding mode coordinated dioxygen to vanadium atom resulted in the repulsion of O_2_ from the coordination environment of [V^V^(=O)(η^1^-Ο_2_)(κ^3^-L^1–4^)(OH_2_)]^**·+**^ complexes.

It is clear, that the reactivity of the vanadium complexes [V^IV^(=O)(κ^3^-L^1–6^)(OH_2_)_2_]^+^ with O_2_ is also dependent on the distance of the vanadium atom from the equatorial plane (N_3_O), and thus, the larger distance the more difficult for the O_2_ to approach the vanadium binding site. This fact justifies the high activity of [V^IV^(=O)(κ^3^-L^1–4^)(OH_2_)]^+^ vs the inactivity of [V^IV^(=O)(κ^3^-L^5,6^)(OH_2_)]^+^ toward O_2_ reduction.

**Figure 9:**
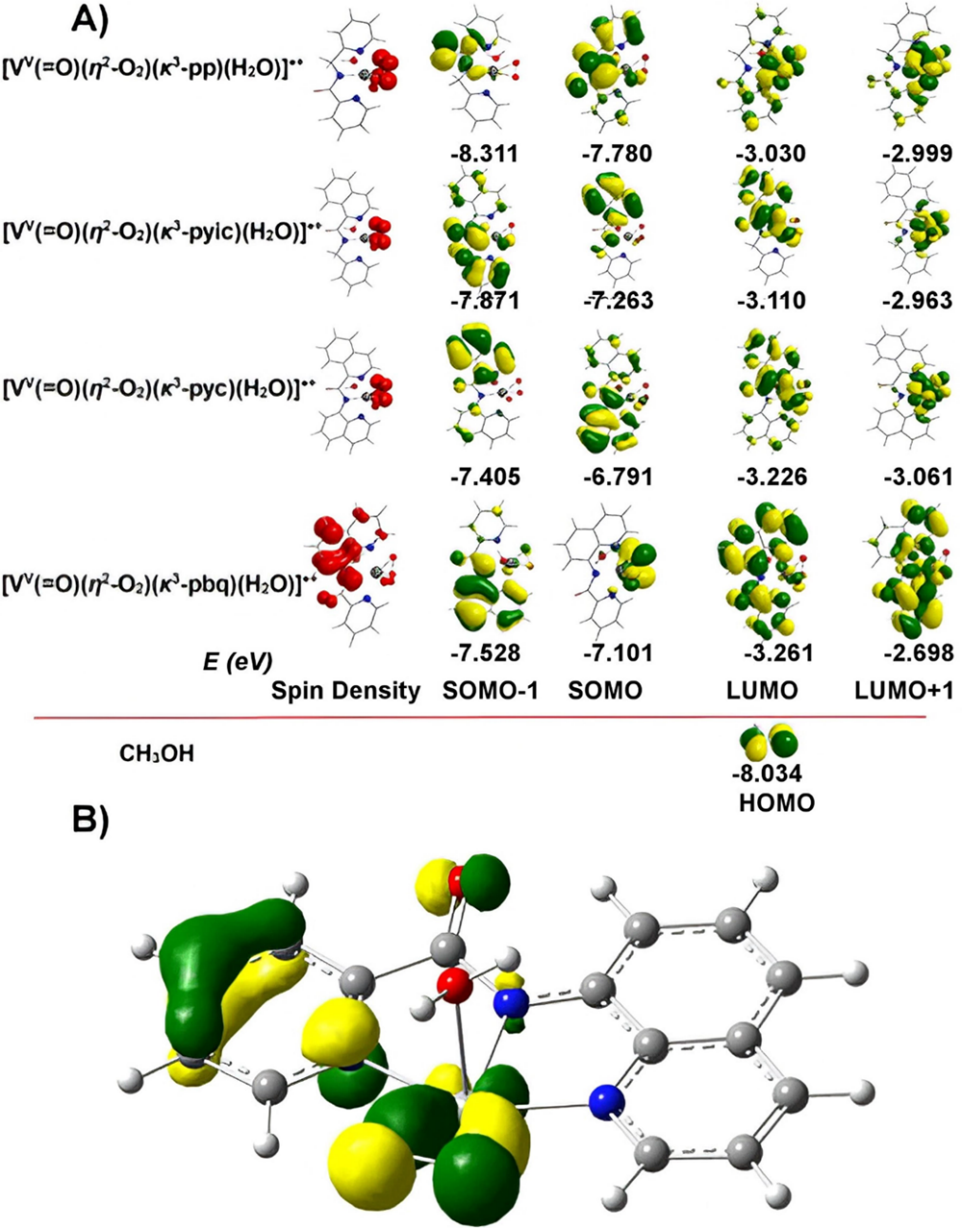
(A) 3D plots of the spin density distribution and frontier molecular orbitals of the [V^V^(=O)(η^2^-O_2_)(κ^3^-L^1–4^)(OH_2_)]^·^^+^ complexes calculated at the PBE0/Def2-TZVP(V)∪6-31+G(d)(E) level of theory in aqueous solution. (L^1^ = pbq^–1^, L^2^ = pp^–1^, L^3^ = pyc^–1^, L^4^ = pyic^–1^). (B) The η^2^ binding mode of the O_2_ ligand to the metal center is exemplified by the respective bonding MO, arising from the in-phase combination of Ο_2_ π* orbitals with the vanadium d AO.

The spin density in the [V^IV^(=O)(κ^3^-L^1–6^)(OH_2_)_2_]^+^ complexes is totally localized on the vanadium metal center, while the lowest unoccupied π-type MOs (LUMO) and LUMO+1 are localized on the L^–^ ligands (Figures 9 and S36). The nature of the [V^IV^(=O)(κ^3^-L^1–6^)(OH_2_)_2_]^+^ MOs suggests their interaction with the π HOMO of O_2_ leading to η^2^-O_2_ bonding mode in the [V^V^(=O)(η^2^-O_2_)(κ^3^-L^1–4^)(OH_2_)]^·^^+^ complexes. The estimated O–O bond distances in the [V^V^(=O)(η^2^-O_2_)(κ^3^-L^1–4^)(OH_2_)]^·+^ complexes found in the range 1.274–1.395 Å are longer than that of 1.203 Å for “free” O_2_ and very close to the estimated O–O bond distance of 1.330 Å for a “free” peroxido (O_2_^·–^) radical.

The spin density of the [V^V^(=O)(η^2^-O_2_)(κ^3^-pbq)(OH_2_)]^·+^ complex is distributed to the pbq^–^ ligand, whereas, in the [V^V^(=O)(η^2^-O_2_)(κ^3^-L^2–4^)(OH_2_)]^·+^ (L_2_ = pp^–1^, L_3_ = pyc^–1^, L_4_ = pyic^–1^) complexes, is localized on the η^2^-O_2_^·**–**^ bonded dioxygen (Figure S36) dictating the oxidation of V(IV) to V(V). The different spin density distribution pattern in the [V^V^(=O)(η^2^-O_2_)(κ^3^-L^1–4^)(OH_2_)]^·+^ complexes is associated with the different frontier molecular orbitals (FMO) pattern of [V^V^(=O)(η^2^-O_2_)(κ^3^-L^1–4^)(OH_2_)]^·+^ relative to the remaining [V^V^(=O)(η^2^-O_2_)(κ^3^-L^1–4^)(OH_2_)]^·+^ complexes.

The bonding σ(V–O) NBOs are constructed from the interaction of spd hybrid orbitals. (13% s, 30% p, and 56% d character) of V with an sp hybrid (18% s and 81% p-character) on oxygen donor atoms and are described as σ(V–O) = 0.44*h*_V_ + 0.88*h*_O_. The bonding σ(O–O) NBOs are constructed from the interaction of sp hybrid orbitals of the oxygen donor atoms of the superperoxido radical (14–16% s and 86–84% p character) for all complexes, except [V^V^O(η^2^-O_2_)(pbq)(OH_2_)]^·+^ where sp hybrid orbitals have 8% *s* and 92% *p*-character and are described as σ(O–O) = 0.71*h*_O(1)_ + 0.71*h*_O(2)_. On the other hand the π(O–O) NBOs are constructed from the overlap of 2p orbitals of the oxygen donor atoms. Notice that π(O–O) NBOs are not formed in the [V^V^O(η^2^-O_2_)(κ^3^-pbq)((OH_2_)]^·+^ complex. The occupancy of the π(O–O) NBOs is nearly to 1 |e| indicating that the single electron resides on the π(O–O) NBO of the coordinated superperoxido radical. According to the NBO analysis the O atoms of the superperoxido radical in [V^V^O(η^2^-O_2_)(κ^3^-L^2–4^)(OH_2_)]^·^^+^ complexes are almost neutral acquiring natural atomic charges of −0.002 up to 0.010 |*e*|. In the [V^V^O(η^2^-O_2_)(κ^3^-pbq)(OH_2_)]^·^^+^ complex the O atoms of the peroxido moiety bear negative natural atomic charges of −0.254 and −0.257 |*e*| indicating that electron density is transferred from the pbq^–^ ligand to η^2^-O_2_ bonded moiety which accounts for the observed spin density distribution on the pbq^–^ ligand in the [V^V^(=O)(η^2^-O_2_)(κ^3^-pbq)(OH_2_)]^·^^+^ complex (Figures S37 and S38).

**Figure 10:**
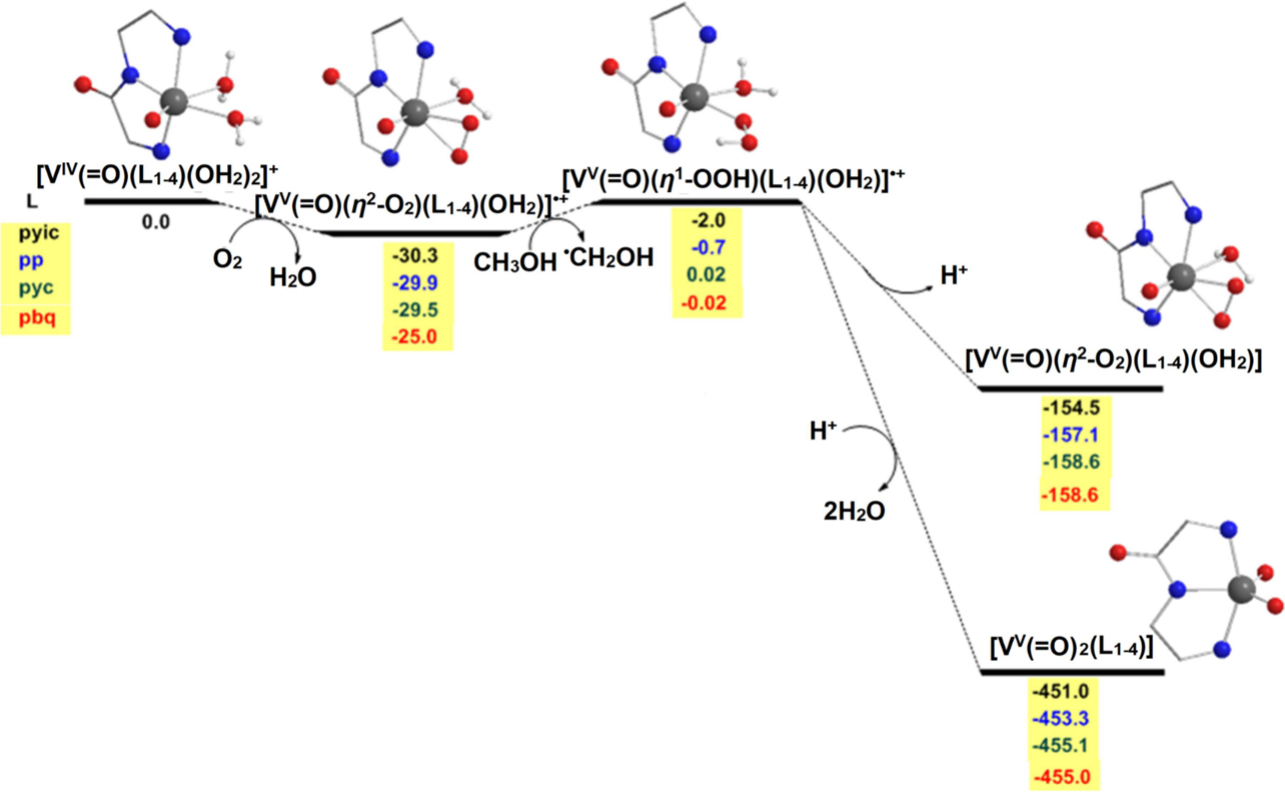
Free energy (Δ*G* in kcal/mol) reaction profiles of the reductive activation of O_2_ to O_2_^2–^ by the [V^IV^(=O) κ^3^-L_1–4_)(OH_2_)_2_]^+^ complexes following the mononuclear O_2_ activation reaction pathway calculated at the PBE0/Def2-TZVP(V)∪6-31+G(d)(E) level of theory in aqueous solution.

The next step in the reductive activation of O_2_ to O_2_^2–^ would involve a H atom abstraction from H atom donating reducing molecules (e.g., CH_3_OH, hydroquinone) by the [V^V^(=O)(η^2^-O_2_)(κ^3^-L^1–4^)(OH_2_)]^·^^+^ intermediates yielding the hydroperoxido, [V^V^(=O)(κ^3^-L_1–4_)(OH_2_)(η^1^-O–OH)]^+^ intermediates (Figure 10). Both the nature of the LUMO and LUMO+1 MOs and the charge distribution on the peroxido moiety support the interaction of the H atom donating reducing agents, with the [V^V^(=O)(η^2^-O_2_)(κ^3^-L^1–4^)(OH_2_)]^·^^+^ intermediates to afford the [V^V^(=O)(κ^3^-L^1–4^)(OH_2_)(η^1^-O–OH)]^+^ species. Deprotonation of [V^V^(=O)(κ^3^-L^1–4^)(OH_2_)(η^1^-O–OH)]^+^ will give the peroxido species.

The dioxido species can be obtained by the reduction of [V^V^(=O)(η^2^-O_2_)(κ^3^-L^1–4^)(OH_2_)]^·^^+^ intermediates by CH_3_OH or/and [V^IV^O(κ^3^-L^1–4^)(OH_2_)_2_]^+^, giving both [V^V^(=O)(η^2^-O_2_)(κ^3^-L^1–4^)(OH_2_)] and *cis*-[V^V^(=O)_2_(κ^3^-L^1–4^)] (Figure 10, eq 9). However, protonation of the distal oxygen atom of [V^V^(=O)(κ^3^-L^1–4^)(OH_2_)(η^1^-O-**O**H)]^+^ bearing the higher negative natural atomic charge yields the transient [V^V^(=O)(κ^3^-L^1–4^)(OH_2_)(η^1^-O**O**H_2_)]^+^ intermediates which easily release a water to produce the *cis*-dioxidovanadium(V) product (Figure 10). This agrees with the experimental data, in which, addition of an acid to the [V^IV^(=O)(κ^3^-L^1–6^)(OH_2_)_2_]^+^/O_2_ methanol solution increases the quantity of *cis*-[V^V^(=O)_2_(κ^3^-L^1–4^)] over the [V^V^(=O)(η^2^-O_2_)(κ^3^-L^1–4^)(OH_2_)] vanadium(V) species.
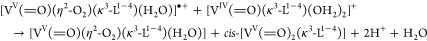
9

#### Binuclear O_2_-Activation Pathway by the [V^IV^(=O)(κ^3^-L)(OH_2_)_2_]^+^ Complexes

**Figure 11:**
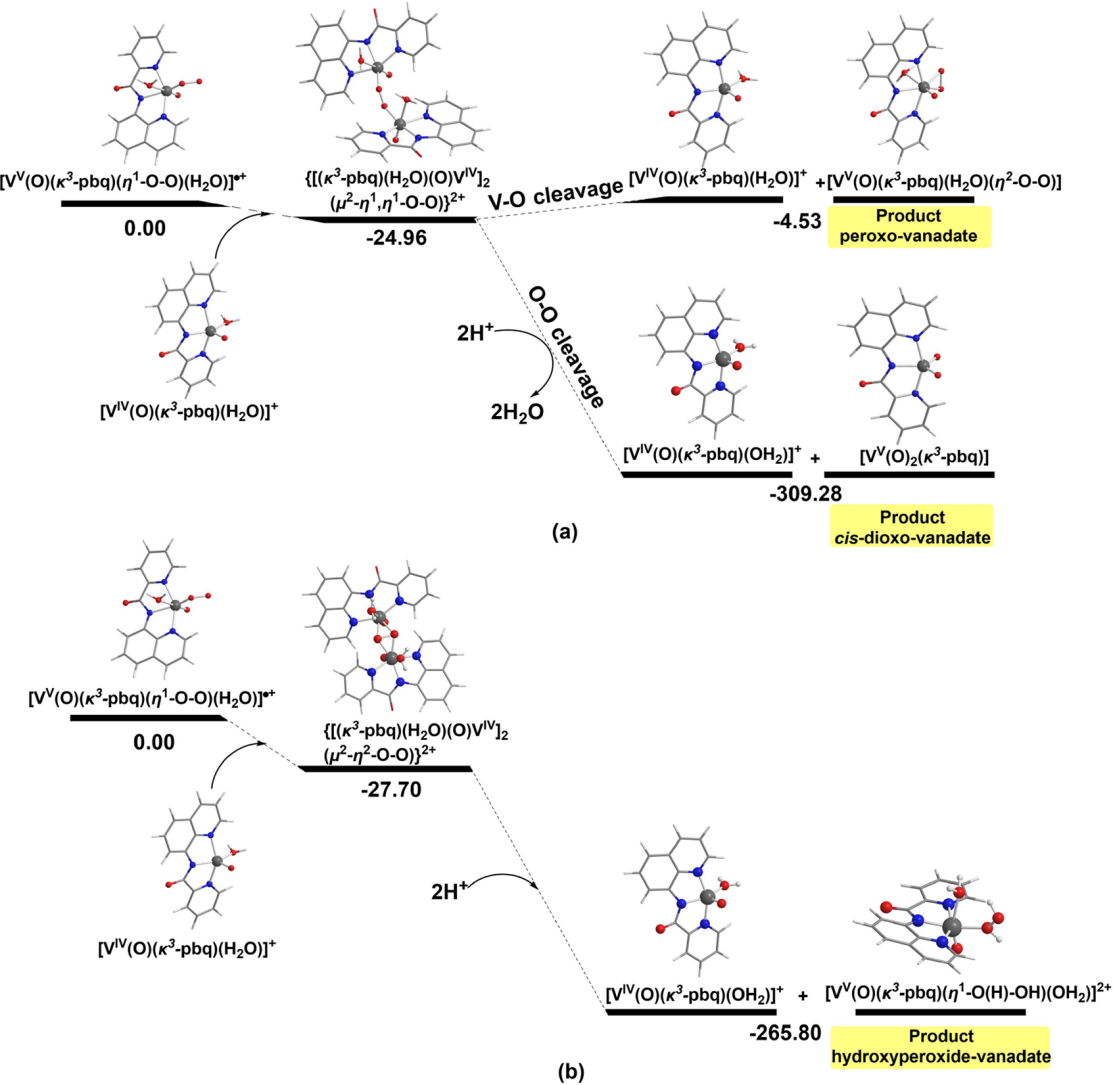
Geometric and energetic profile for the reductive activation of O_2_ by the [V^IV^(=O)(κ^3^-pbq)(OH_2_)_2_]^+^ complex involving (a) μ^2^,η^1^,η^1^-Ο_2_-bridged vanadium dimer intermediate and (b) μ^2^,η^2^-Ο_2_-bridged vanadium dimer intermediate calculated by the PBE0/Def2-TZVP(V)U6-31+G(d)(E)/PCM computational protocol in aqueous solution.

An alternative reaction pathway that leads to the formation of both the peroxido-vanadium(V) and *cis*-dioxido-vanadium(V) products involves the trapping of the reactive [V^V^(=O)(*n*^1^–O-O)(κ^3^-L^1–4^)(OH_2_)]^·^^+^ superoxido monomer by a second [V^IV^(=O)(κ^3^-L^1–4^)(OH_2_)]^+^ species to yield peroxido dimers, formulated as [(H_2_O)(κ^3^-L^1–4^)(O=)V^IV^(μ^2^-*n*^1^,*n*^1^-O-O)V^IV^(=O)(κ^3^-L^1–4^)(H_2_O)]^2+^, in which the superoxido bridge is coordinated to vanadium metal centers in an end-on (*n*^1^,*n*^1^-) coordination mode (Figure 11). In Figure 11b, it is given the energetic profile of the reaction proceeding through the [(H_2_O)(κ^3^-pbq)(O=)V(μ^2^-*n*^2^-O-O)V(=O)(κ^3^-pbq)(H_2_O)]^2+^ dimer intermediate. However, it should be noticed that this path leads to a different product namely the [V(=O)(pbq)(η^1^-O(H)–OH)(OH_2_)]^2+^ species.

The formation of the [(H_2_O)(κ^3^-L^1–4^)(O=)V^IV^(μ^2^-*n*^1^,*n*^1^–O-O)V^IV^(=O)(κ^3^-L^1–4^)(H_2_O)]^2+^ is enthalpically favored by 24.96 kcal/mol (pbq^–^) (Figure 11). The Δ*G* sequence of the formation of the binuclear intermediate follows the pyic^–1^ > pp^–1^ > pyc^–1^ > pb*q*^–1^ > pic^–1^ > qqc^–1^ order, which parallels the experimentally established reactivity.

**Scheme 8:**
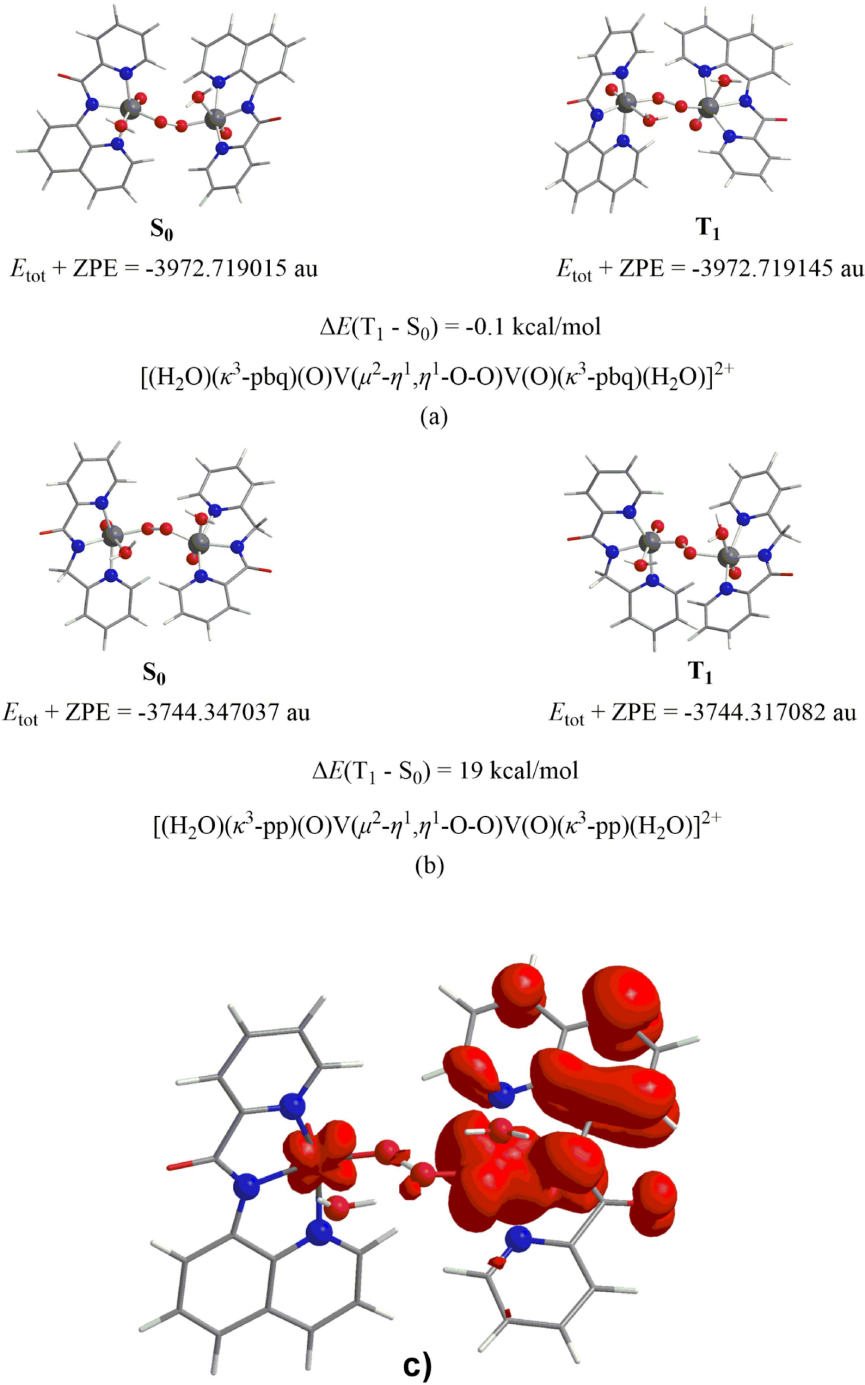
ZPE Corrected Total Electronic Energies, *E*_tot_ + ZPE Calculated for the Optimized Geometries of (a) [(H_2_O)(κ^3^-pbq)(O=)V^IV^(μ^2^-*n*^1^,*n*^1^-O-O)V^IV^(=O)(κ^3^-pbq)(H_2_O)]^2+^ Dimer at S_0_ (Left) and T_1_ (Right) States and (b) [(H_2_O)(κ^3^-pp)(O=)V(μ^2^-*n*^1^,*n*^1^-O-O)V(=O)(κ^3^-pp)(H_2_O)]^2+^ Dimer at S_0_ (Left) and T_1_ (Right) States at the PBE0/Def2-TZVP(V)U6-31+G(d)(E) Level of Theory in Aqueous Solution (c) 3D Isosurface Plot of the Spin Density Calculated for the Triplet State of [(H_2_O)(κ^3^-pbq)(O=)V^IV^(μ^2^-*n*^1^,*n*^1^-O-O)V^IV^(=O)(κ^3^-pbq)(H_2_O)]^2+^ Dimer Intermediate at the PBE0/Def2-TZVP(V)U6-31+G(d)(E)/PCM Level

Notice that, for the dimer with pbq^–^ ligand the first triplet excited state, T_1_ is estimated to be more stable than the respective singlet ground state, S_0_ by about 0.1 kcal/mol (Scheme 8a). This means that both singlet S_0_ and triplet T_1_ states of this dimer are nearly degenerate a phenomenon reported earlier^109^ for other dimeric complexes among them and a Vanadium(IV) dimer with maltolato ligands.^110^ In contrast, for the vanadium complexes with ligands L^2^-L^4^, for example the S_0_ state of the dimer with the pp^–^ ligand, is more stable than the respective T_1_ state by about 19 kcal/mol (Scheme 8b) in line with the absence of a signal for the intermediates of these compounds.

The natural atomic charges (in blue), Wiberg bond order (WBO) and 3D plots of FMOs of intermediates calculated by the PBE0/Def2-TZVP(V)∪6-31+G(d)(E)/PCM computational protocol in aqueous solution are given in the SI (Figure S39). In the [(H_2_O)(κ^3^-pbq)(O=)V^IV^(μ^2^-*n*^1^,*n*^1^-O-O)V^IV^(=O)(κ^3^-pbq)(H_2_O)]^2+^ intermediate the two V–OO and O–O bond distances were computed to be 1.832, 1.807, and 1.365 Å, respectively.

In Scheme 8c, it is depicted the spin density calculated for the triplet state of the μ^2^, η^1^, η^1^-Ο_2_-bridged vanadium dimer intermediate (Figure 11). It is obvious that the unpaired electrons reside on both the metal centers and in part on one of the ligands. This is in line with the experimental findings obtained from the EPR measurements.

The [(H_2_O)(κ^3^-pbq)(O=)V^IV^(μ^2^-*n*^1^,*n*^1^-O-O)V^IV^(=O)(κ^3^-pbq)(H_2_O)]^2+^ intermediate complex can undergo either the V–O bond cleavage affording the *cis*-dioxo-vanadium(V) product with release of 284.32 kcal/mol or V–OO bond cleavage affording the peroxido-vanadium(V) product and the oxidized [V^V^(=O)(κ^3^-pbq)(OH_2_)]^2+^ species (Figure 11). The V–OO bond cleavage demands a relatively low bond dissociation energy of 20.43 kcal/mol.

According to NBO population analysis, the O atoms of the peroxido bridge in [(H_2_O)(κ^3^-pbq)(O=)V^IV^(μ^2^-*n*^1^,*n*^1^-O-O)V^IV^(=O)(κ^3^-pbq)(H_2_O)]^2+^ acquiring negative natural atomic charges (−0.170 and −0.195 |*e*|) are activated toward the exothermic protonation or H atom acquisition yielding the [(H_2_O)(κ^3^-pbq)(O=)V^IV^(μ^2^-*n*^1^,*n*^1^-O–OH)V^IV^(=O)(κ^3^-pbq)(H_2_O)]^3+^ and [(H_2_O)(κ^3^-pbq)(O=)V^IV^(μ^2^-*n*^1^,*n*^1^-HO–OH)V^IV^(=O)(κ^3^-pbq)(H_2_O)]^4+^ intermediates. The estimated WBO(O–O) value of 1.022 indicates a single bond character for the O–O bond.

It is evident that in the next step the dihydroperoxido [(H_2_O)(κ^3^-pbq)(O=)V^IV^(μ^2^-*n*^1^,*n*^1^-HO–OH)V^IV^(=O)(κ^3^-pbq)(H_2_O)]^4+^ intermediate could undergo either an O–O or a V–O bond cleavage releasing water or H_2_O_2_ to give the *cis*-dioxido product [V^V^(=O)_2_(κ^3^-pbq)] and an oxidized [V^V^(=O)(κ^3^-pbq)(OH_2_)]^2+^ species.

#### Comparison of the O_2_ Activation Mechanism from the V^IV^/Amidate Complexes with Other V^IV^, Co^II^, and Fe^II^ Compounds

The V^IV^/amidate complexes are superior dioxygen activators to the V^IV^/terpyridine complex. Both classes of complexes contain N_3_ chelate ligands, and the main difference is that the amidate ligands are negatively charged, while the terpyridine ligand is neutral. Apparently, this supports the fact that V^IV^ complexes with negatively charged nitrogenous ligands are better O_2_ activators than the neutral ones.^54−59^ The stabilization of the intermediate (**Id**) by using amidate ligands with an extended π-delocalized system allowed its full spectroscopic and electrochemical characterization. The experimental results of this study revealed that the nature of the intermediate from the direct reaction of [V^IV^(=O)(κ^3^-L^1–4^)(H_2_O)_2_]^+^ with O_2_ is different from the electrochemically synthesized O_2_^·–^ centered radical suggested by Kelm and Kruger.^54,55^

It has been reported in the literature that the reaction of V^IV^ complexes,^54−59^ with O_2_ follows a two-step mechanism through a mononuclear intermediate radical. However, a mononuclear intermediate radical is expected to show the vanadium hyperfine splitting of the organic radical in the cw-EPR spectrum as was observed by Kelm and Kruger. The cw-EPR signals of the intermediates, reported in the literature, were silent suggesting that the intermediate might not be mononuclear. In contrast to the EPR silent intermediates of the oxygenated solutions of the V^IV^ complexes with the ligands L^2–4^ and the compounds reported in the literature, the intermediate of the reaction [V^IV^(=O)(κ^3^-pbq)(H_2_O)_2_]^+^ with O_2_ gives a signal revealing the existence of a binuclear spin coupled V^IV^ molecule.

In this study, theoretical calculations reveal that the species [V^V^(=O)(η^2^-O_2_)(κ^3^-pbq)(H_2_O)]^·+^ can be trapped by a [V^IV^(=O)(κ^3^-pbq)(H_2_O)_2_]^+^ molecule giving a more stable EPR active binuclear intermediate [V^IV^(=O)(κ^3^-pbq)(H_2_O)(η^1^,η^1^-O_2_)V^IV^(=O)(κ^3^-pbq)(H_2_O)]^2+^. Theory also suggests that the spin density of the pbq^–^ intermediates is distributed to the metal ion and the pbq^–^ ligand. The EPR silent intermediates from the reaction of [V^IV^(=O)(κ^3^-L^2–4^)(H_2_O)_2_]^+^ with O_2_ are the binuclear [V^IV^(=O)(κ^3^-L^2–4^)(H_2_O)(η^1^,η^1^-O_2_)V^IV^(=O)(κ^3^-L^2–4^)(H_2_O)]^2+^ species, in which the two spins are antiferromagnetically coupled.

A two-step mechanism has been also proposed for the O_2_ activation by Fe^II^-porphyrin (O_2_ → H_2_O, 4e^–^ reduction) and Co^II^-salophen (O_2_ → O_2_^2–^, 2e^–^ reduction) complexes through direct coordination of O_2_ to the two metal ions.^111−113^ However, there are significant differences compared to the mechanism of the vanadium complexes. O_2_ activation from both Co^II^ and Fe^II^ complexes, in contrast to V^IV^ compounds, is catalyzed by light. The V^IV^-amidate and Co^II^-salophen have zero order *p*O_2_-dependence for the reduction of O_2_ to O_2_^2–^ by the Co^II^-salophen catalyst.^112,113^ In contrast, the rate of the reduction of O_2_ to H_2_O by the Fe^II^(porphyrin) complex follows a first-order kinetics toward [O_2_]. Both Co^II^ and Fe^II^ compounds follow a first-order rate law for the O_2_ reduction with respect to [H^+^], thus suggesting a protonated hydroperoxide intermediate [M-η^1^-O–OH]. In contrast, H^+^ does not accelerate the 2e^–^ reduction of O_2_ to O_2_^2–^ by the V^IV^-amidate catalysts, proposing that protonation of V-η^2^-O_2_ is not a rate-determining step in agreement with the theoretical calculations. However, the presence of protons favors the 4e^–^ than the 2e^–^ reductive activation of O_2_ by the V^IV^-amidate catalysts.

#### Galvanic Cell

The performances of cell **A** {Zn|V^III^,V^II^||V^V^O_2_^+^,V^IV^O^2+^,H_2_O_2_|C(s)} and cell **B** {Zn|V^III^,V^II^||[**Id**, [V^IV^O(κ^3^-bpq)(H_2_O)_2_]^+^|O_2_|C(s)} were examined and compared. The pbq^–^ vanadium complexes were chosen for the battery over the other vanadium amidate compounds because they form the most stable intermediate (larger lag time).

The cell functioned according to the following equations
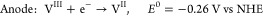
10
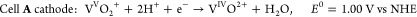
11
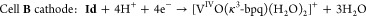
12

The discharge products, V^III^ ions at the anode and V^VI^O^2+^ions at the cathode, are chemically charged (regenerated) by zinc and hydrogen peroxide or O_2_ in respective reactors according to eqs 13–15:
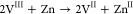
13
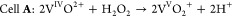
14
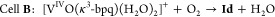
15

The overall reactions are described in eqs 16 and 17
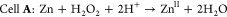
16
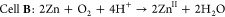
17

**Figure 12:**
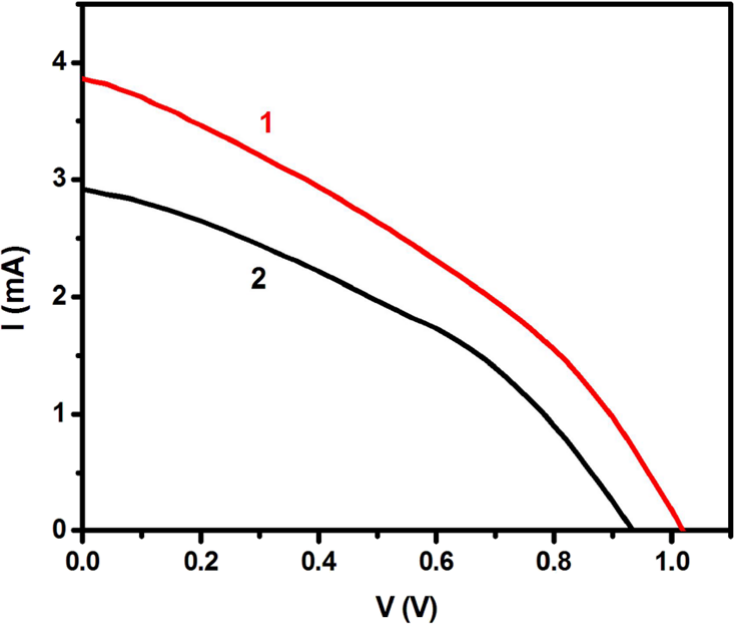
Current–voltage plots for cell **A** (red line 1) and cell **B** (black line 2).

In both cells, oxidation of the vanadium in cathode (Figure S40) was clearly marked by change of color from light blue to dark brown. There is not any precipitation of [V^V^O(η^2^-O_2_)(κ^3^-pbq)(H_2_O)] or *cis*-[V^V^O_2_(κ^3^-pbq)] in the solution. The main species in the cathodic solution is the **Id**. This agrees with the triphenylphosphine ^1^H NMR experiment (*vide supra*) that provides evidence for [V^V^O(η^2^-O_2_)(κ^3^-pbq)(H_2_O)] to be formed only after the most of the reducing agent has been consumed. Therefore, [V^V^O(η^2^-O_2_)(κ^3^-pbq)(H_2_O)] will not be formed until the fuel in anode is consumed. The current–voltage plots for the two cells **A** and **B** are shown in Figure 12.

Both cells gave similar current–voltage curves. The difference in current is due to differences in functional concentration of the redox species. The difference in the short-circuit voltage (at zero current) is due to the difference between the cathode electrolytes and consequently to the intrinsic cell potential.

**Figure 13:**
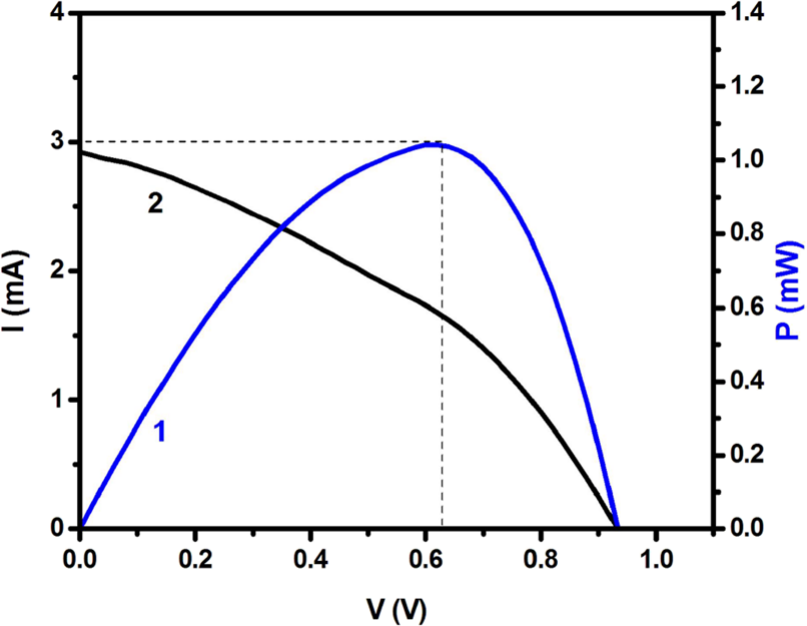
Power production by the cell **B** (blue line, 1). The black line, 2, is the current–voltage curve for cell **B**.

The cell gives maximum power output at about 0.61 V generating a short current of 3 mA (Figure 13). These data show that an inexpensive functional cell can be made by employing Hbpq as an intermediate of V^IV^OSO_4_·3.5H_2_O oxidation, which instead of using H_2_O_2_, generates H_2_O_2_ in situ from atmospheric O_2_. Another striking feature of the present cell is that it does not require solar light or other source of energy for the production of H_2_O_2_, allowing power production development through this technology to be compact, cost-effective, and durable.^9,18,114^

## Conclusions

Reaction of V^IV^O^2+^ with nitrogeneous amidate ligands (HL^1–6^) bearing an extended π-delocalized aromatic system, −1 charge upon deprotonation of the amide nitrogen atom and, in the presence of O_2_, resulted in the 2e^–^ reduction of O_2_ to O_2_^2–^ and formation of the pentagonal-bipyramidal peroxidovanadium(V), [V^V^(=O)(η^2^-O_2_)(κ^3^-L^1–4^)(H_2_O)] and the trigonal bipyramidal dioxido *cis*-[V^V^(=O)_2_(κ^3^-L^1–6^)] complexes. The judiciously chosen features of the designed ligands resulted in the thermodynamic stabilization of the intermediate radicals, allowing their full spectroscopic and electrochemical characterization. Time-dependent spectroscopic investigation, variation of ligand steric interactions, EPR, ES-MS, and theoretical calculations revealed that the mechanism of O_2_ activation from [V^IV^(=O)(κ^3^-L^1–4^)(H_2_O)_2_]^+^ is through the formation of a binuclear [V^IV^(=O)(κ^3^-L^1–4^)(H_2_O)(η^1^,η^1^-O_2_)V^IV^(=O)(κ^3^-L^1–4^)(H_2_O)]^2+^ intermediate. Furthermore, the stabilization of the intermediate enhanced the reactivity of V^IV^O^2+^ complexes, resulting in the synthesis of the most reactive V^IV^ complexes reported so far, toward to the 2e^–^ reduction of O_2_.

A galvanic cell using [V^IV^(=O)(κ^3^-pbq)(H_2_O)_2_]^+^, *cis*-[V^V^(=O)_2_(κ^3^-pbq)]/O_2_ as a cathode has been constructed, exhibiting a similar power output with the V^IV^O^2+^,V^V^O_2_^+^/H_2_O_2_ cathode cell; thus, demonstrating that this new technology can find applications in fuel cell and other energy related uses. The new cell, described in this report, has the advantage of the in situ generation of H_2_O_2_, thus decreasing the overall cost and becoming environmentally friendly assuring environmental and economical sustainability.

The new technology developed in this study opens new avenues, proposing the replacement of the peroxido vanadium(V) compounds with the intermediate radicals of the V^IV^O^2+^-L adducts with O_2_ in several applications such as catalytic activation of hydrocarbons and activation of O_2_ in metal-air batteries allowing their extensive commercialization. Furthermore, the amidate ligands mimic proteins and the reactivity of [V^IV^(=O)(L)(H_2_O)_2_]^+^ with O_2_ helps to further understand the bioactivity of vanadates in biological systems, targeting at the development of antidiabetic and/or anticancer drugs.
