# Supplementary material for: Experimental and Theoretical Investigation of the Mechanism of the Reduction of O2 from Air to O22– by VIVO2+–N,N,N-Amidate Compounds and Their Potential Use in Fuel Cells
Source: Inorg Chem. 2024 Feb 6;63(7):3229–49. doi: 10.1021/acs.inorgchem.3c03272 (PMC10880062; doi:10.1021/acs.inorgchem.3c03272)
Supplement: Supplementary file 1 — ic3c03272_si_001.pdf [file ic3c03272_si_001.pdf]

# Supporting Information

## Experimental and Theoretical Investigation of the Mechanism of the Reduction of $O_2$ from Air to $O_2^{2-}$ by $V^{IV}O^{2+}$–*N*,*N*,*N*-Amidate Compounds and Their Potential Use in Fuel Cells

Michael Papanikolaou,[a] Sofia Hadjithoma,[a] Odysseas Keramidas,[a] Chryssoula Drouza,[b] Angelos Amoiridis,[a] Alexandros Themistokleous,[a] Sofia C. Hayes,[a] Haralampos N. Miras,*[c] Panagiotis Lianos,*[d] Athanassios C. Tsipis,*[e] Themistoklis A. Kabanos*[e] and Anastasios D. Keramidas,*[a]

[a]*Department of Chemistry, University of Cyprus, Nicosia 2109, Cyprus. E-mail: akeramid@ucy.ac.cy*

[b]*Department of Agricultural Sciences, Biotechnology and Food Science, Cyprus University of Technology, Limassol 3036, Cyprus. E-mail: chryssoula.drouza@cut.ac.cy*

[c] *School of Chemistry, The University of Glasgow. Glasgow G12 8QQ (UK). Email: Charalampos.moiras@glasgow.ac.uk*

[d]*Department of Chemical Engineering, University of Patras, 26500 Patras, Greece, E-.mail: lianos@upatras.gr*

[e]*Section of Inorganic and Analytical Chemistry, Department of Chemistry, University of Ioannina, Ioannina 45110, Greece. E-mail: tkampano@uoi.gr and attsipis@uoi.grb*

**Contents**

**Experimental** S5

**Figure S1.** ORTEP diagrams of $[V^{IV}O(qqc)(\mu\text{-}O)V^{V}O(qqc)](HSO_4).H_2O$ (**8**) with atomic numbering scheme and thermal ellipsoids at 50% probability level. S9

**Figure S2.** ORTEP diagrams of **1'** with atomic numbering scheme and thermal ellipsoids at 50% probability level. S9

**Figure S3.** Diagram of the mean *d*(V-($\eta^2$-$O_2$) *vs* *d*(O-O) of all monoperoxo complexes in the literature up to now (black filled circles). The mean value of all the bond lengths is 1.869 Å for *d*(V-($\eta^2$-$O_2$) and 1.424 Å for *d*(O-O) (red rhomb). All values are obtained from Cambridge Crystallographic Database. S10

**Figure S4.** ORTEP diagrams of **4** with atomic numbering scheme and thermal ellipsoids at 50% probability level. S10

**Figure S5.** ORTEP diagrams of **6** with atomic numbering scheme and thermal ellipsoids at 50% probability level. S11

**Figure S6.** ORTEP diagrams of **9** with atomic numbering scheme and thermal ellipsoids at 50% probability level. S11

**Figure S7.** DFT optimized structures of A) **8** and B) **7**. S12

**Figure S8.** IR(ATR) spectra of **7**. S13

**Figure S9.** Resonance Raman spectra with excitation at 368.9 nm of the final V-$O_2$ product after reaction with (black) $^{16}O_2$ and (blue) $^{18}O_2$. The asterisks denote solvent bands (dichloromethane). The vertical dashed lines denote areas of change due to isotopic substitution. S14

**Figure S10.** UV-vis spectrum of a methanol solution of **1** (1.96 x $10^{-4}$ M). S15

**Figure S11.** UV-vis spectrum of a methanol solution of $[V^{V}(=O)(\eta^2\text{-}O_2)(\kappa^3\text{-pp-}N_{py},N_{am},N_q)(H_2O)]$. Concentration 0.85 x $10^{-4}$ M. S15

**Figure S12**. UV-vis spectrum of a methanol solution of $HL_4$ (red line) and $[V^{V}(=O)(\eta^2\text{-}O_2)(\kappa^3\text{-pyc-}N_{py},N_{am},N_q)(H_2O)]$ (black line). Concentrations for both 0.96 x $10^{-4}$ M. S16

**Figure S13.** UV-vis spectrum of a methanol solution of Hpyic (red line) and **3** (black line). Concentrations for both 1.92 x $10^{-4}$ M. S16

**Figure S14.** UV-vis spectrum of a methanol solution of Hpic (red line) and **5** (black line). Concentrations for both 1.92 x $10^{-4}$ M. S17

**Figure S15.** UV-vis spectrum of a chloroform solution of **6**. Concentration 1.92 x $10^{-4}$ M. S17

**Figure S16.** Cyclic voltammographs of $CH_3CN$ or $CH_2Cl_2$ solutions of $pbq^-$, $[V^{V}O_2(pbq)]$ and $[V^{V}O(\eta^2\text{-}O_2)(pbq)(H_2O)]$. Working electrode: glassy carbon, Auxiliary electrode: platinum, Reference electrode: Ag/AgCl. Scan Rate 100 mV/s. S18

**Figure S17.** Cyclic voltammographs of $CH_3CN$ solution of $[V^{V}O(\eta^2\text{-}O_2)(pbq)(H_2O)]$ at various scan rates. Working electrode: glassy carbon, Auxiliary electrode: platinum, Reference electrode: Ag/AgCl. S19

**Figure S18.** A) The X-band cw EPR spectrum of $V^{IV}(=O)^{2+}$-Hpbq - $O_2$ system in a frozen (120 K) solution (full black line) and the simulated spectrum (dotted red line) considering three paramagnetic vanadium complexes present in solution $[V^{IV}(=O)(H_2O)_5]^{2+}$, $[V^{IV}(=O)(pbq)(H_2O)_2]^{+}$ and **Id**. B) The spectra of the **Id** (red dotted line) and the experimental (full black line) reproduced from the simulation parameters using the following parameters for both vanadium centers $g_x$= 1.970 $g_y$=1.993 $g_z$=1.955 $A_x$=22 x $10^{-4}$ cm$^{-1}$ $A_y$=23 x $10^{-4}$ cm$^{-1}$ $A_z$=150 x $10^{-4}$ cm$^{-1}$ $A_x$'=9 x $10^{-4}$ cm$^{-1}$ $A_{y|}$'=5 x $10^{-4}$ cm$^{-1}$ $A_z$=9 x $10^{-4}$ cm$^{-1}$ and lwpp=2.0 mT, and 592 x $10^{-4}$, 247 x $10^{-4}$ and 241 x $10^{-4}$ cm$^{-1}$ isotropic, antisymmetric and symmetric electron–electron coupling. Arrows show the peaks used for the simulation. C) EPR signal at the half-field of the forbidden $\Delta M_s$=2 transition (black line) and simulation using the parameters of B (red line). S20

**Figure S19**. Positive ion mass spectrum of the $VOSO_4$ : $C_{15}H_{11}N_3O$ reaction mixture in $H_2O$:$CH_3OH$ (1:3) after 2 hrs of reaction time. The isotopic distribution envelopes correspond to singly charged species centred at 332.05, 347.01, 646.04, 698.05 and 716.06 m/z and can be attributed to the mono- and binuclear vanadium species. S21

**Figure S20.** A) The X-band cw EPR spectra of $V^{IV}OSO_4{\cdot}3.5H_2O$ (0.0144 M)-Hpbq (0.0138 M) in solution *vs*. time, one spectrum every 20 min, and assignments of the three species, B) overlay of all spectra from 0 to 240 min. S22

**Figure S21.** The X-band cw EPR spectra of $V^{IV}OSO_4{\cdot}3.5H_2O$ (0.0144 M)-Hpic (0.0138 M) in $H_2O$:$CH_3OH$ (25:75, v/v) frozen solutions vs time. S22

**Figure S22. (A)** $^1H$ NMR spectra of a $V^{IV}OSO_4{\cdot}3.5H_2O$ (0.0144 M)-Hpbq (0.0138 M) in solution *vs*. time (first 130 min), and proton assignments. **(B)** $^1H$ NMR spectra of a $V^{IV}OSO_4{\cdot}3.5H_2O$ (0.0144 M)-Hbpq (0.0138 M) in solution *vs*. time (first 7 days) and assignments of the peaks of compounds $[V^V(=O)(\eta^2\text{-}O_2)(\kappa^3\text{-pbq})(H_2O)]$ and *cis*-$[V^V(=O)_2(\kappa^3\text{-pbq})]$. S23

**Figure S23.** $^1H$ NMR spectra of the reaction of the $D_2O$:$CH_3OH$ (25:75, v/v) solution of $V^{IV}OSO_4{\cdot}3.5H_2O$ (0.0144 M)-Hpyc (0.0138 M)- *vs* time (h) and assignments. S24

**Figure S24.** $^1H$ NMR spectra of the reaction of the $D_2O$:$CH_3OH$ (25:75, v/v) solution of $V^{IV}OSO_4{\cdot}3.5H_2O$ (0.0144 M)-Hqqc (0.0138 M)- *vs* time (h) and assignments. S24

**Figure S25.** Graph of ln([**1**]) *vs* ln(Initial Rates) ([**1**] =4.0 $10^{-4}$–1.6 $10^{-3}$ M) and linear fitting. S25

**Figure S26.** $^{51}V$ NMR spectra of the reaction of the $D_2O$:$CH_3OH$ (25:75, v/v) solution of $V^{IV}OSO_4{\cdot}3.5H_2O$ (0.0144 M)-Hpyc (0.0138 M)- *vs* time (h) and assignments. The V1, V2, V4, V5 peaks are assigned to vanadates monomer, dimer, tetramer and pentamer respectively originated from the external aqueous $NaVO_3$ solution used as quantitative standard S26

**Figure S27.** $^{51}V$ NMR spectra of the reaction of the $D_2O$:$CH_3OH$ (25:75, v/v) solution of $V^{IV}OSO_4{\cdot}3.5H_2O$ (0.0144 M)-Hpyic (0.0138 M)- *vs* time (h) and assignments. The V1, V2, V4, V5 peaks are assigned to vanadates monomer, dimer, tetramer and pentamer respectively originated from the external aqueous $NaVO_3$ solution used as quantitative standard S27

**Figure S28.** Concentration of **2L** and **3L** species *vs* time diagram of (25:75, v/v) $D_2O$:$CD_3OD$ solution of $V^{IV}OSO_4{\cdot}3.5H_2O$ (0.0144 M)-L (0.0138 M). S28

**Figure S29.** $^{51}V$ NMR spectra of the reaction of the $D_2O$:$CH_3OH$ (25:75, v/v) solution of $V^{IV}OSO_4{\cdot}3.5H_2O$ (0.0144 M)-Hpbq (0.0138 M)-hydroquinone (0.0138 M) with $O_2$ *vs* time (h) and assignments. The V1, V2, V4, V5 peaks are assigned to vanadates monomer, dimer, tetramer and pentamer respectively originated from the external aqueous $NaVO_3$ solution used as quantitative standard S29

**Figure S30.** Graph showing the concentration of the vanadium species *vs* time of the $D_2O$:$CH_3OH$ (25:75, v/v) solution of a $V^{IV}OSO_4{\cdot}3.5H_2O$ (0.0144 M)-Hpbq (0.0138 M) a) without $H_2SO_4$ (red circles) and b) with $H_2SO_4$ (0.0050 M) (black circles). Species concentrations were calculated by integration of $^{51}V$ NMR peaks of the spectra. The filled shapes represent the concentration of **1**, empty shapes represent the concentration of **4** S30

**Figure S31.** $^1H$ NMR spectra of a (25:75, v/v) $D_2O$:$CD_3OD$ solution of $V^{IV}OSO_4{\cdot}3.5H_2O$ (0.0144 M)-Hpbq (0.0138 M)-hydroquinone (0.0138 M) vs time (first 7 days) and assignments. S31

**Figure S32.** $^1H$ NMR spectra of a (25:75, v/v) $D_2O$:$CD_3OD$ solution of $V^{IV}OSO_4{\cdot}3.5H_2O$ (0.0144 M)-Hpbq (0.0138 M)-triphenylphosphine (0.0276 M) vs time (first 12 days) and assignments. S31

**Figure S33.** $^1H$ NMR spectra of a (25:75, v/v) $D_2O$:$CD_3OD$ solution of $V^{IV}OSO_4{\cdot}3.5H_2O$ (0.0144 M)-Hpbq (0.0138 M)-triphenylphosphine (0.276 M) vs time (first 12 days) and assignments. S32

**Figure S34**. Geometric reaction profiles of the reductive activation of $O_2$ to $O_2^{2-}$ by the other $[V^{IV}O(L)(OH_2)_2]^+$ (L = pp, pyic, pyc, pbq, qqc, pic) complexes with selected structural parameters (bond lengths in Å) calculated at the PBE0/Def2-TZVP(V)$\cup$6-31+G(d)(E) level of theory in aqueous solution. S33
**Figure S35**. Optimized geometries of the peroxo-bridged dinuclear $\{[(L)(H_2O)(O)V]_2(\mu^2$-$\kappa^1,\kappa^1$-O-O)$\}^{2+}$ and their monoprotonated intermediates with selected structural parameters (bond lengths in Å) calculated at the PBE0/Def2-TZVP(V)$\cup$6-31+G(d)(E) level of theory in aqueous solution. S34
**Figure S36**. 3D-plots of the spin density distribution and frontier molecular orbitals of representative $[V^{IV}O(L)(OH_2)]^+$ complexes calculated at the PBE0/Def2-TZVP(V)$\cup$6-31+G(d)(E) level of theory in aqueous solution. S35
**Figure S37**. 3D-plots of the *BD*(V-O) and *BD*(O-O) NBOs of repreentative $[V^VO(L)(\kappa^2$-$O_2)(OH_2)]^{+\bullet}$ complexes calculated at the PBE0/Def2-TZVP(V)$\cup$6-31+G(d)(E) level of theory in aqueous solution. S35
**Figure S38**. 3D-plots of the frontier molecular orbitals, LUMO composition and electrostatic potential fitting atomic charges, q(ESP), on V, proximal $O_{(1)}$ and distal $O_{(2)}$ atoms of the $[V^VO(pbq)(\kappa^1$-OOH$)(OH_2)]^+$ complexes calculated at the PBE0/Def2-TZVP(V)$\cup$6-31+G(d)(E) level of theory in aqueous solution. S36
**Figure S39**. Equilibrium geometries, natural atomic charges (in blue), *WBO*s and 3D plots of FMOs of intermediates calculated by the PBE0/Def2-TZVP(V)$\cup$6-31+G(d)(E)/PCM computational protocol in aqueous solution. S37
**Figure S40.** SEM images of the structure of the carbon cloth electrode (left) and the mesoporous carbon film (right) S38
**Table S1.** Crystallographic and experimental data for **1**$\cdot H_2O$ and **1'.** S39
**Table S2.** Crystallographic and experimental data for **4** and **5.** S40
**Table S3.** Crystallographic and experimental data for **6** and **9**.MeOH**.** S41
**Table S4**. Bond lengths [Å] and angles [°] for **1**. S42
**Table S5**. Bond lengths [Å] and angles [°] for **1'**. S44
**Table S6.** Bond lengths [Å] and angles [°] for **2** S46
**Table S7.** Bond lengths [Å] and angles [°] for **4** S49
**Table S8.** Bond lengths [Å] and angles [°] for **5.** S50
**Table S9.** Bond lengths [Å] and angles [°] for **6** S51
**Table S10.** Bond lengths [Å] and angles [°] for **9** S53
**Table S11.** Resonance Raman peaks (excitation at 368.91 nm) experimental, calculated by theory (values in parenthesis) and assignments. S54
**Table S12.** $^1$H NMR chemical shifts and assigmants of ligands and complexes **1-6**. S55
**Table S13**. $^{51}$V NMR chemical shifts (ppm) of the 25:75 $D_2O$:$CD_3OD$ solution of peroxido and dioxide vanadium complexes. S56
**Table S14.** Redox potentials (mV) and assignments of the waves from the CVs of the $CH_3CN$ solutions of pdb$^-$ and the complexes $[V^{IV}O(pbq)(H_2O)]^+$, $[V^VO_2(pbq)]$, $[V^VO(\eta^2$-$O_2)(pbq)(H_2O)]$ and $[V^VO(\eta^2$-$O_2)(pbq)(H_2O)]$. S56

**Experimental**

***NMR Spectroscopy***. NMR spectra were recorded on a Bruker Avance 500 spectrometer at 500 MHz for $^{1}H$ and 131.75 MHz for $^{51}V$. A 30º-pulse width, 3000 Hz spectral window, 1 s relaxation delay and a 90º -pulse width, 25000 Hz spectral window, 0.1 s relaxation delay was applied for $^{1}H$ and $^{51}V$ NMR respectively. The purity of the ligands was confirmed by NMR spectroscopy. The samples were prepared from purified molecules in $CD_3OD$ at room temperature just prior to NMR spectrometric measurements. A finger insert to NMR tube containing an aqueous solution of $NaV^{V}O_3$ was used as quantification external standard. The experiments of the oxidation of hydroquinone and triphenylphosphine from $O_2$ was performed by monitoring concurrently two solutions one with the presence of the catalyst and one blank without the presence of the catalyst. The relation of the vanadium concentration in the insert with that of the solution in the NMR tube was calculated by comparing the integrals of the vanadate peaks in the insert tube and the peaks of a standard aqueous peroxo-vanadate solution in the outer tube, made by mixing $NaV^{V}O_3$ and excess of $H_2O_2$. Data acquisition and processing were accomplished using TopSpin 4.0.6 and MultiSpecNMR 4.0.0. Standard pulse programs as implemented in TopSpin were used for data acquisition.

***UV-vis Spectroscopy.*** The UV-vis measurements of the compounds were recorded on a Photonics UV-vis spectrophotometer Model 400, equipped with a CCD array, operating in the range 250 to 1000 nm. Data processing was accomplished using MultiSpecUVVIS 1.0.0. Single crystals of either **1**·$H_2O$ or **2** were dissolved in $CH_3OH$ in concentration ranging from 1.0 x $10^{-5}$ to 2.0 x $10^{-3}$ M. The extinction coefficients of the peaks were calculated from the spectra and used for calculation of the concentration of the vanadium(IV/V) compounds during their reaction with the atmospheric $O_2$. The UV-vis spectra of the reaction of various concentrations of equimolar $V^{IV}OSO_4 \cdot 3.5H_2O$-Hpbq in $CH_3OH$:$H_2O$ solutions (75:25, v/v), with continuous bubbling of atmospheric $O_2$ or pure $O_2$, were recorded *vs* time, at either 1 min or 10 min time

intervals. The pseudo first order $k_{int}$ reaction was calculated from the slope of the graph ln(initial reaction rates) at $t = 0$ *vs* the ln(complex **1** concentration). The initial reaction rates for various concentrations of the equimolar $V^{IV}OSO_4 \cdot 3.5H_2O$-Hpbq solutions were calculated from the absorption at 374 nm of the first 30 spectra obtained in the time period of 30 min.

***X-ray Structure Analysis*.** Details of crystallographic and experimental data for $\mathbf{1} \cdot H_2O$**, 2** are depicted in Table S1. X-ray diffraction data of single crystals of the vanadium(V) peroxo compounds were collected by means of a Xcalibur Oxford diffractometer equipped with a Sapphire 3 CCD detector and a 4-cycle Kappa geometry goniometer, using enhanced Mo *K*□ ($\lambda$ = 0.71073 Å) X-ray source and graphite radiation monochromator. Analytical absorption correction was applied using CrysAlis RED software. CrysAlis CCD and CrysAlis RED software were used for data collection and data reduction/cell refinement respectively.[1, 2] The structure of the compounds was solved by direct methods and refined by full-matrix least-squares techniques on $F^2$ by using SHELXS-97.[3,4] Special computing molecular graphics incorporated in the WinGX 3.2 interface were used.[5] All the non-H atoms were anisotropically refined. The positions of hydrogen atoms in all structures were calculated from stereochemical considerations and kept fixed isotropic during refinement or found in DF map and refined with isotropic thermal parameters.

***EPR spectroscopy.*** The c. w. X-band EPR spectra of the reaction of equimolar $V^{IV}OSO_4 \cdot 3.5H_2O$-Hpbq (0.10-2.0 mM) in $CH_3OH$:$H_2O$ solutions (75:25, v/v) with atmospheric $O_2$ *vs* time at room temperature and at 10 min - time intervals, were measured on an ELEXSYS E500 Bruker spectrometer at resonance frequency ~9.44 GHz, modulation frequency 100 MHz, modulation phase 0.0 deg, modulation amplitude 1.000 G, sweep width 2500 G, power attenuation 20.0 dB, number of scans 10, conv. time 9.7 ms, and at 130 K. The resonance frequency was accurately measured with solid DPPH ($g$ = 2.0036). Data acquisition and

processing were accomplished using Xepr and MultiSpecEPR 4.0.0 softwares. The spectra were simulated using the software EasySpin 5.0.0.[6]

***Electrochemistry.*** Cyclic voltammetry (CV) and round disk voltammetry (RDV) experiments were recorded using an EG&G Princeton Applied Research 273A potentiostat/galvanostat. Electrochemical procedures were performed with a three-electrode configuration: a platinum disk or rotating disk electrode (RDE) was used as the working electrode, a platinum wire as the auxiliary electrode, and Ag/AgCl (0.20 V *vs* NHE) (mixali check) as reference. The potential of the reference electrode was measured with ferrocene (0.65 V *vs* NHE). All the potential values are referred to NHE. The electrochemical measurements were carried out in $CH_3CN$ solutions of $But_4NClO_4$ (0.1 M) purged with $N_2$ prior to the measurements where was required at 298 K. Scan rate (ν) of 100 mV $s^{-1}$ was used for cyclic voltammograms and linear sweep voltammetry experiments.

***IR and Resonance Raman measurements.*** IR measurements were obtained on a Shimadzu. Resonance Raman (RR) measurements were obtained by using instrumentation described in detail previously.[7] The 368.9 nm excitation wavelength employed in the RR experiments was produced via Raman shifting the second harmonic from a Q-switched Nd:YAG laser (PRO-230, 30 Hz, Spectra Physics) at 532 nm. About 100 μL of the sample were placed in a spinning cell arrangement consisting of an EPR quartz tube (diameter: 4 mm) attached to a rheostat-controlled motor for choice of rotation speed. Use of the spinning cell prolonged the lifetime of the samples. Even though relatively high excitation energy was used (33 μJ/pulse); the RR spectra of the compounds did not show any alterations over time. The Raman scattered light was collected in a backscattering geometry and delivered to a 0.75 m focal-length Czerny-Turner spectrograph, equipped with a 2400-grooves/mm holographic grating. The slit was set to 150 μm providing for 5 $cm^{-1}$ spectral resolution at the wavelength employed in this study. The scattered light was detected by a $LN_2$-cooled, 2048 x 512 pixel, back-illuminated UV-enhanced

CCD detector (Spec10:2KBUV/LN, Princeton Instruments). Each spectrum presented is the accumulation of 10-20 10-min spectra. Frequency calibration was accomplished with the use of cyclohexane. MATLAB was used for spectral treatment and analysis.

***Galvanic Cell.*** The cell is comprised of a Zn anode electrode and a cathode electrode, made of carbon cloth with added carbon black film (Figure S16) having 1.0 $cm^2$ active area. Nafion was employed as ion transfer membrane separating anode from cathode compartment. The anode compartment was filled with an aqueous solution of 0.1 M $V^{IV}OSO_4 \cdot 3.5H_2O$ and 0.5 M $H_2SO_4$ reduced by an excess of Zn dust, while the cathode compartment was filled with two different electrolytes: either 0.1 M $V^{IV}OSO_4 \cdot 3.5H_2O$ and 0.5 M $H_2SO_4$ oxidized with excess of $H_2O_2$ (cell **A**) or 0.1 M $V^{IV}OSO_4 \cdot 3.5H_2O$ and 0.1 M Hpbq dissolved in a mixture of 25% water and 75% methanol containing 0.5 M $NaClO_4$ (cell **B**). The distance between anode and cathode electrodes was ~1 cm.

**ESI-MS Experimental Details**

All MS data were collected using a Q-trap, time-of-flight MS (Maxis Impact MS) instrument supplied by Bruker Daltonics Ltd. The detector was a time-of-flight, micro-channel plate detector and all data was processed using the Bruker Daltonics Data Analysis 4.1 software, whilst simulated isotope patterns were investigated using Bruker Isotope Pattern software and Molecular Weight Calculator 6.45. The calibration solution used was Agilent ES tuning mix solution, Recorder No. G2421A, enabling calibration between approximately 100 *m/z* and 2000 *m/z*. This solution was diluted 60:1 with MeCN. The reaction mixture was introduced into the MS *via* direct injection at 180 µL $h^{-1}$. The ion polarity for all MS scans recorded was positive, at 180 $^{o}C$, with the voltage of the capillary tip set at 4000 V, endplate offset at –500 V, funnel 1 RF at 300 Vpp and funnel 2 RF at 400 Vpp.

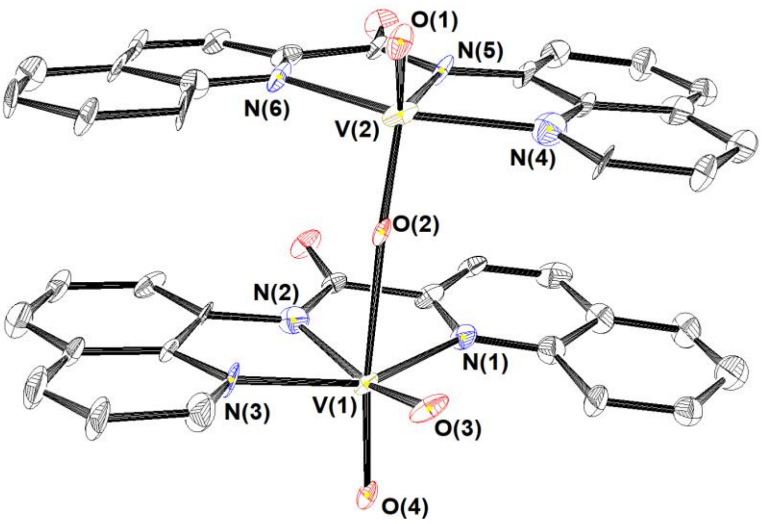


**Figure S1.** ORTEP diagrams of $[V^{IV}O(qqc)(\mu\text{-}O)V^{V}O(qqc)](HSO_4).H_2O$ (**8**) with atomic numbering scheme and thermal ellipsoids at 50% probability level.

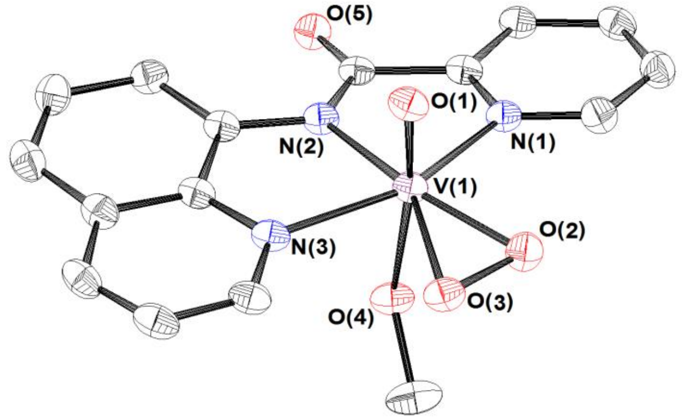


**Figure S2.** ORTEP diagrams of **1’** with atomic numbering scheme and thermal ellipsoids at 50% probability level.

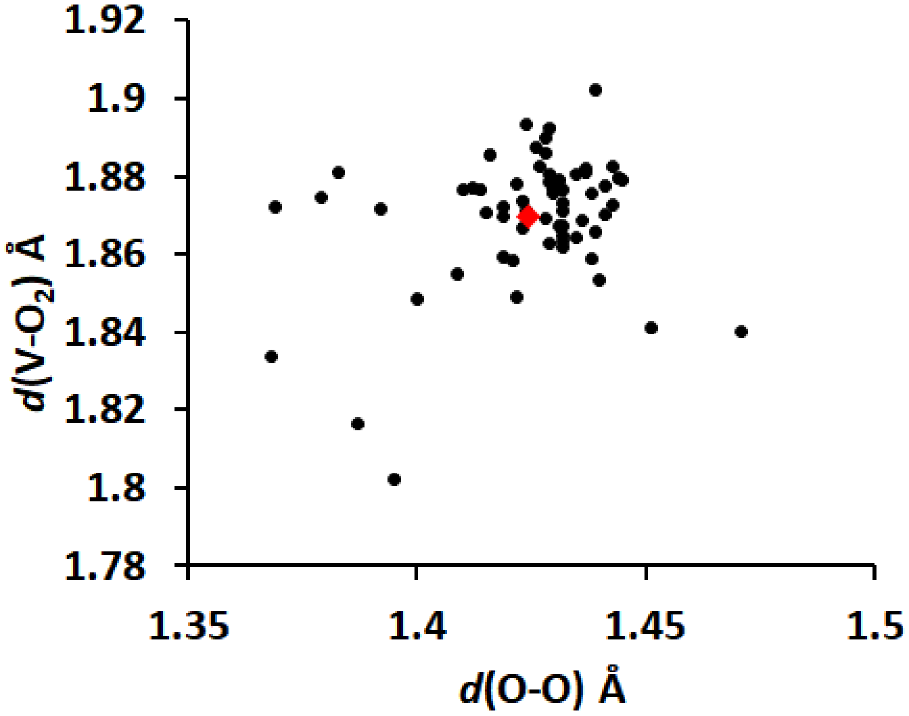


**Figure S3.** Diagram of the mean *d*(V-($\eta^2$-$O_2$) *vs* *d*(O-O) of all monoperoxo complexes in the literature up to now (black filled circles). The mean value of all the bond lengths is 1.869 Å for *d*(V-($\eta^2$-$O_2$) and 1.424 Å for *d*(O-O) (red rhomb). All values are obtained from Cambridge Crystallographic Database.

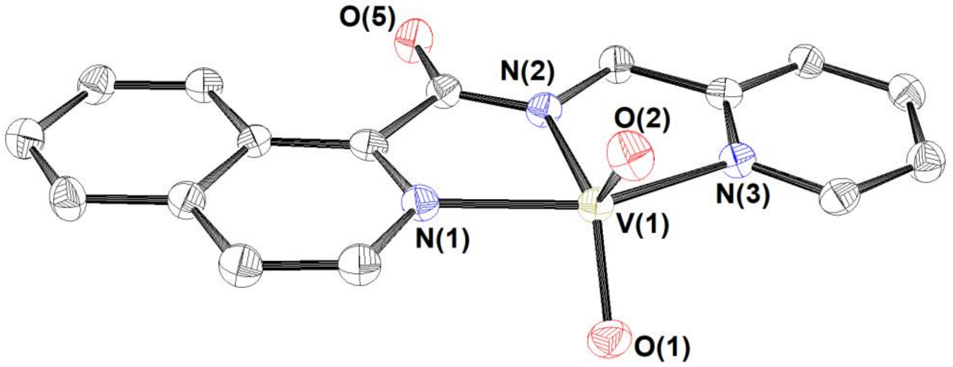


**Figure S4.** ORTEP diagrams of **4** with atomic numbering scheme and thermal ellipsoids at 50% probability level.

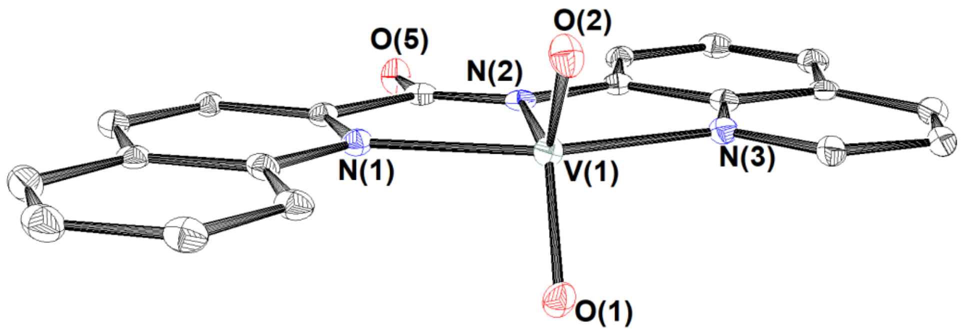


**Figure S5.** ORTEP diagrams of **6** with atomic numbering scheme and thermal ellipsoids at 50% probability level.

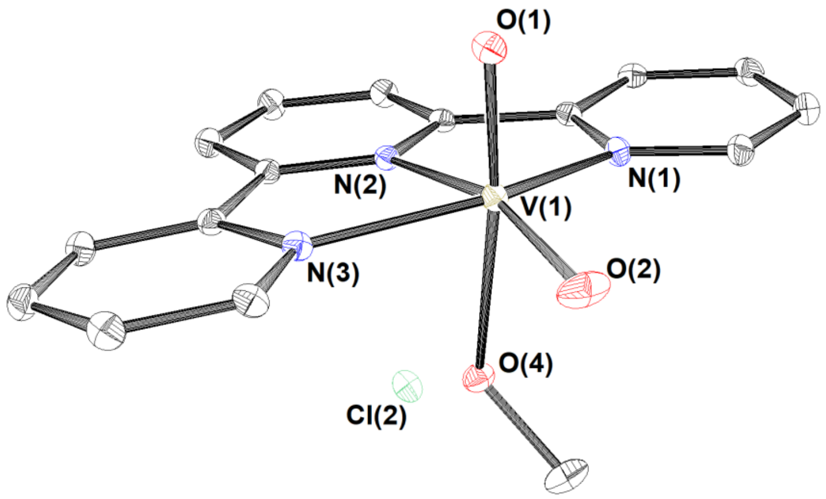


**Figure S6.** ORTEP diagrams of **9** with atomic numbering scheme and thermal ellipsoids at 50% probability level.

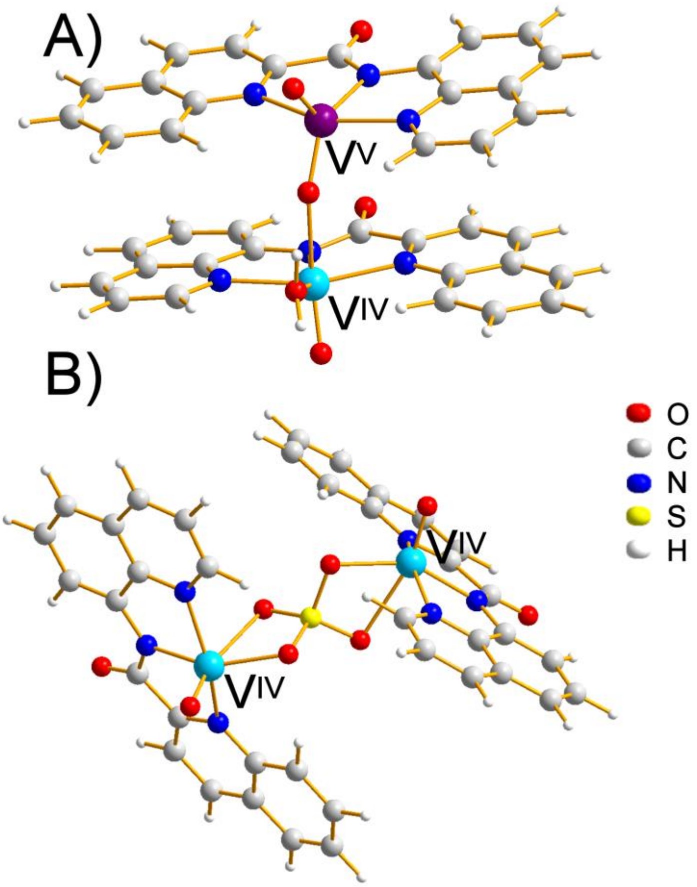


**Figure S7.** DFT optimized structures of A) **8** and B) **7**.

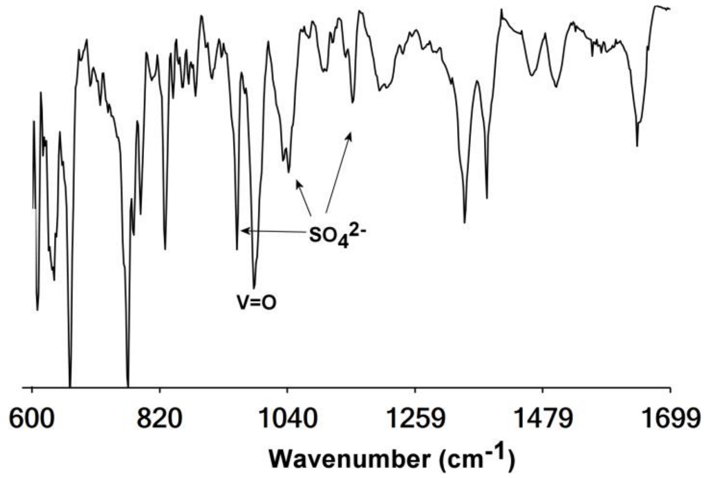


**Figure S8.** IR(ATR) spectra of **7**.

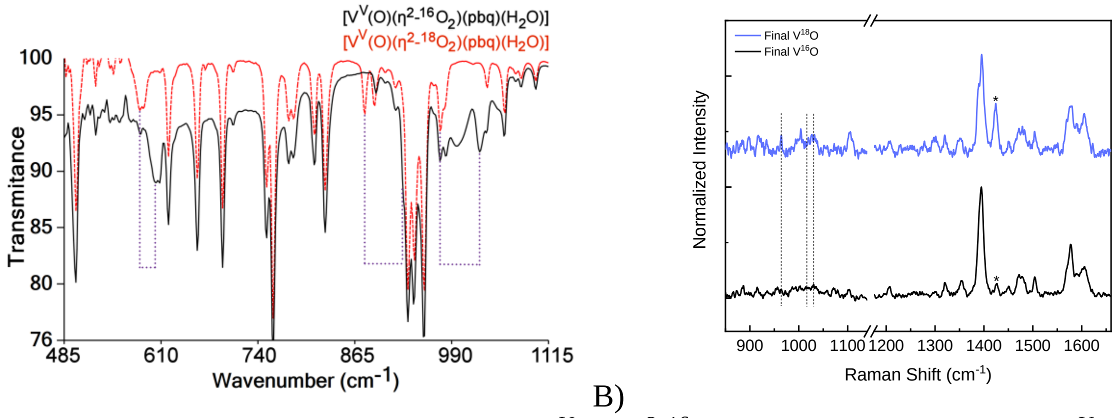


A) B)

**Figure S9.** A) IR(ATR) spectra of [$V^V$(O)(η$^2$-$^{16}O_2$)(pbq)($H_2O$)].$H_2O$ and $V^V$(O)(η$^2$-$^{18}O_2$)(pbq)($H_2O$)].$H_2O$ and B) Resonance Raman spectra of $CH_2Cl_2$ solutions of [$V^V$(O)(η$^2$-$^{16}O_2$)(pbq)($H_2O$)].$H_2O$ and $V^V$(O)(η$^2$-$^{18}O_2$)(pbq)($H_2O$)].$H_2O$ with excitation at 368.9 nm of the final V-$O_2$ product after reaction with (black) $^{16}O_2$ and (blue) $^{18}O_2$. The asterisks denote solvent bands. Dashed lines denote areas of change due to isotopic substitution. Strong bands of the free ligand at 1394 and 1577 $cm^{-1}$, assigned to C=C and C=N stretching and C-H bending (Table S11) appear shifted upon coordination, originated to C=N, C=C bond length changes. A broad band observed at 1020 $cm^{-1}$ in the of [$V^V$(=O)($\eta^2$-$^{16}O_2$)($\kappa^3$-pbq)($H_2O$)].$H_2O$ appears to split into two bands in the [$V^V$(=O)($\eta^2$-$^{18}O_2$)($\kappa^3$-pbq)($H_2O$)].$H_2O$. Deconvolution of this broad band in the two spectra shows that in the V-$^{16}O_2$ complex a third band at 1018 $cm^{-1}$ contributes to the broadening of the peak and is attributed to the O-O stretching of the bridging dioxygen. Isotopic substitution with $^{18}O_2$, shifts this band to 962 $cm^{-1}$, in the same vicinity as the V=O stretching band at 957 $cm^{-1}$, leaving behind the two bands at 993 and ~1030 $cm^{-1}$ due to pyridine ring breathing.

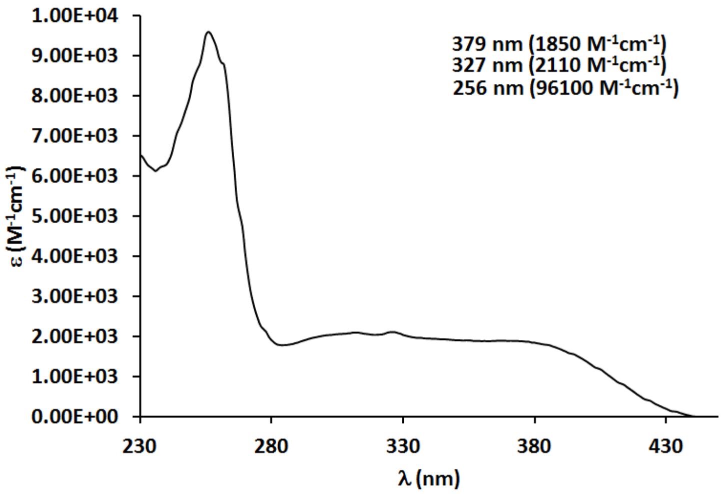


**Figure S10.** UV-vis spectrum of a methanol solution of **1** (1.96 x $10^{-4}$ M).

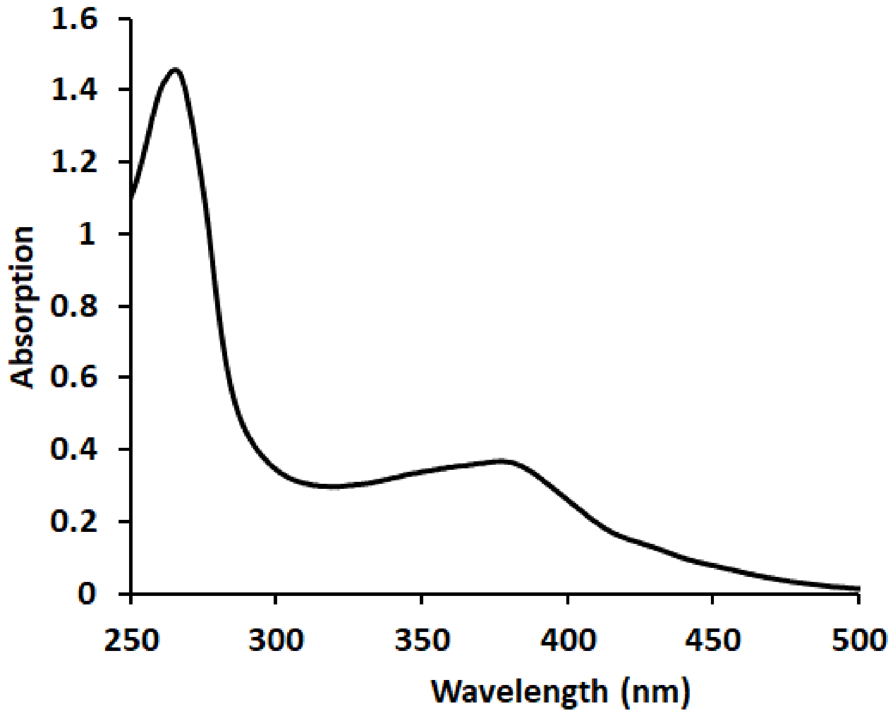


**Figure S11.** UV-vis spectrum of a methanol solution of [$V^V$(=O)($\eta^2$-$O_2$)($\kappa^3$-pp-$N_{py}$,$N_{am}$,$N_q$)($H_2O$)]. Concentration 0.85 x $10^{-4}$ M.

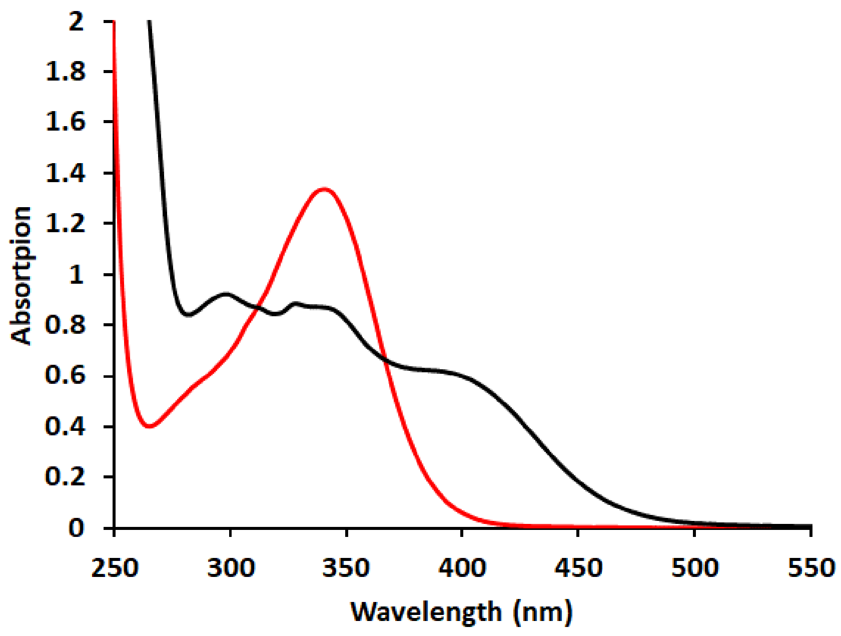


**Figure S12**. UV-vis spectrum of a methanol solution of $HL_4$ (red line) and [$V^V$(=O)($\eta^2$-$O_2$)($\kappa^3$-pyc-$N_{py}$,$N_{am}$,$N_q$)($H_2O$)] (black line). Concentrations for both 0.96 x $10^{-4}$ M.

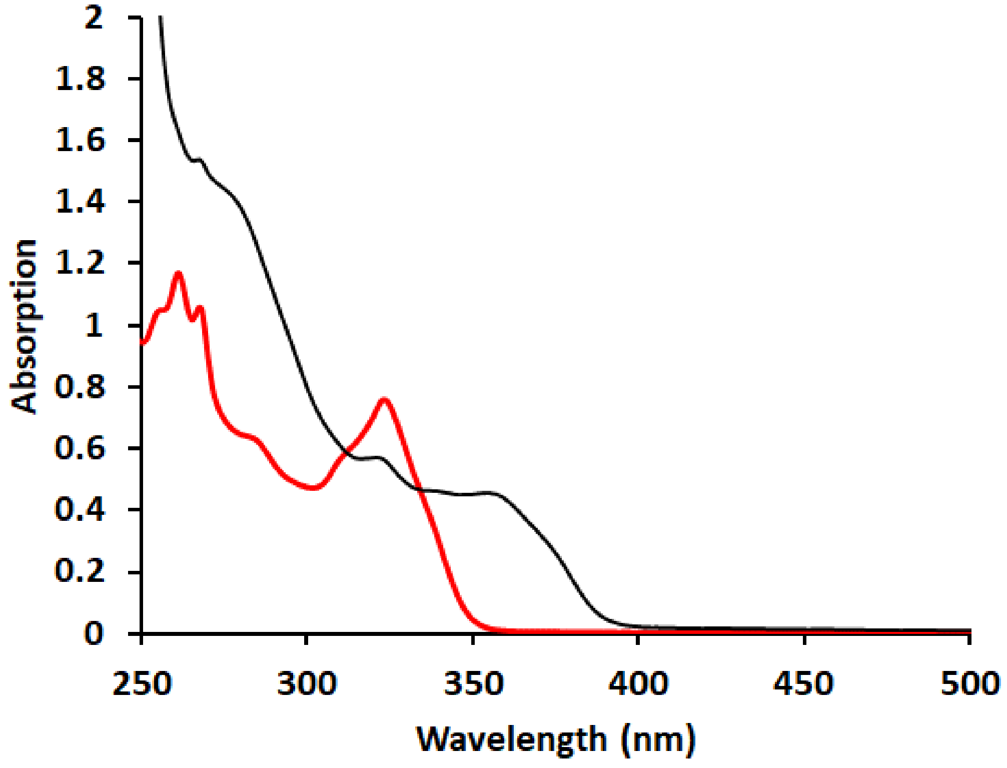


**Figure S13.** UV-vis spectrum of a methanol solution of Hpyic (red line) and **3** (black line). Concentrations for both 1.92 x $10^{-4}$ M.

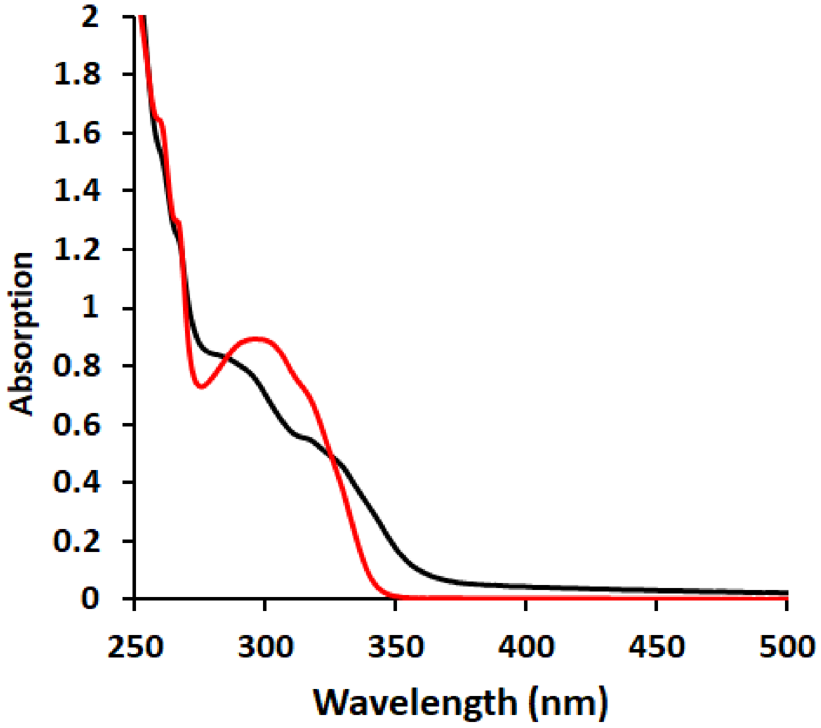


**Figure S14.** UV-vis spectrum of a methanol solution of Hpic (red line) and **5** (black line). Concentrations for both 1.92 x $10^{-4}$ M.

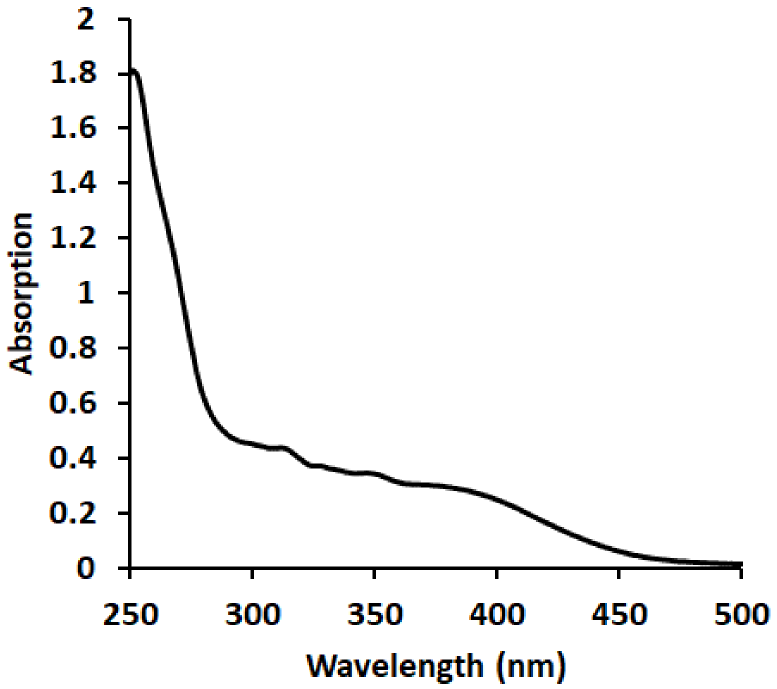


**Figure S15.** UV-vis spectrum of a chloroform solution of **6**. Concentration 1.92 x $10^{-4}$ M.

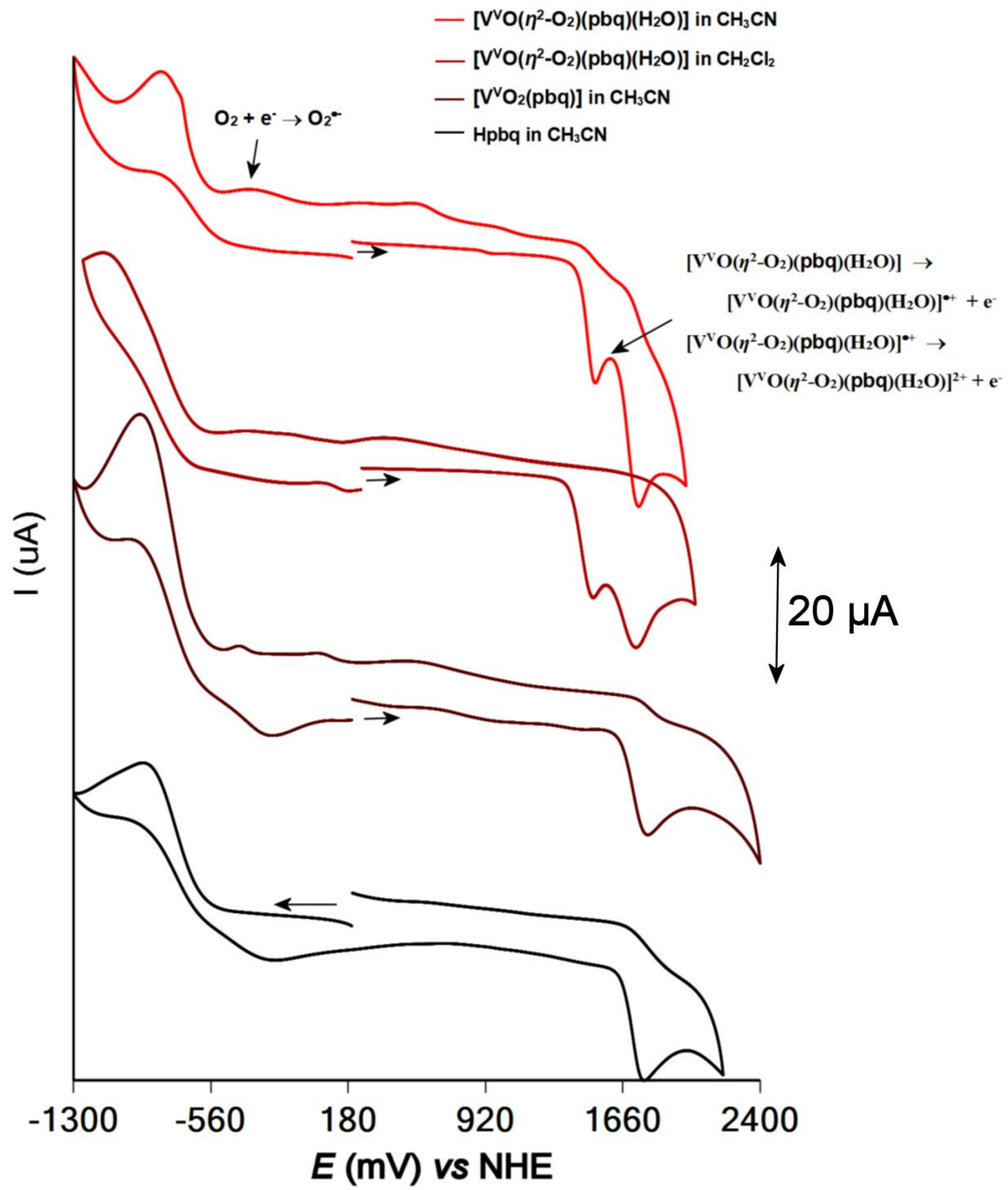


**Figure S16.** Cyclic voltammographs of $CH_3CN$ or $CH_2Cl_2$ solutions of pdb$^-$, [$V^VO_2$(pbq)] and [$V^VO(\eta^2$-$O_2$)(pbq)($H_2O$)]. Working electrode: glassy carbon, Auxiliary electrode: platinum, Reference electrode: Ag/AgCl. Scan Rate 100 mV/s.

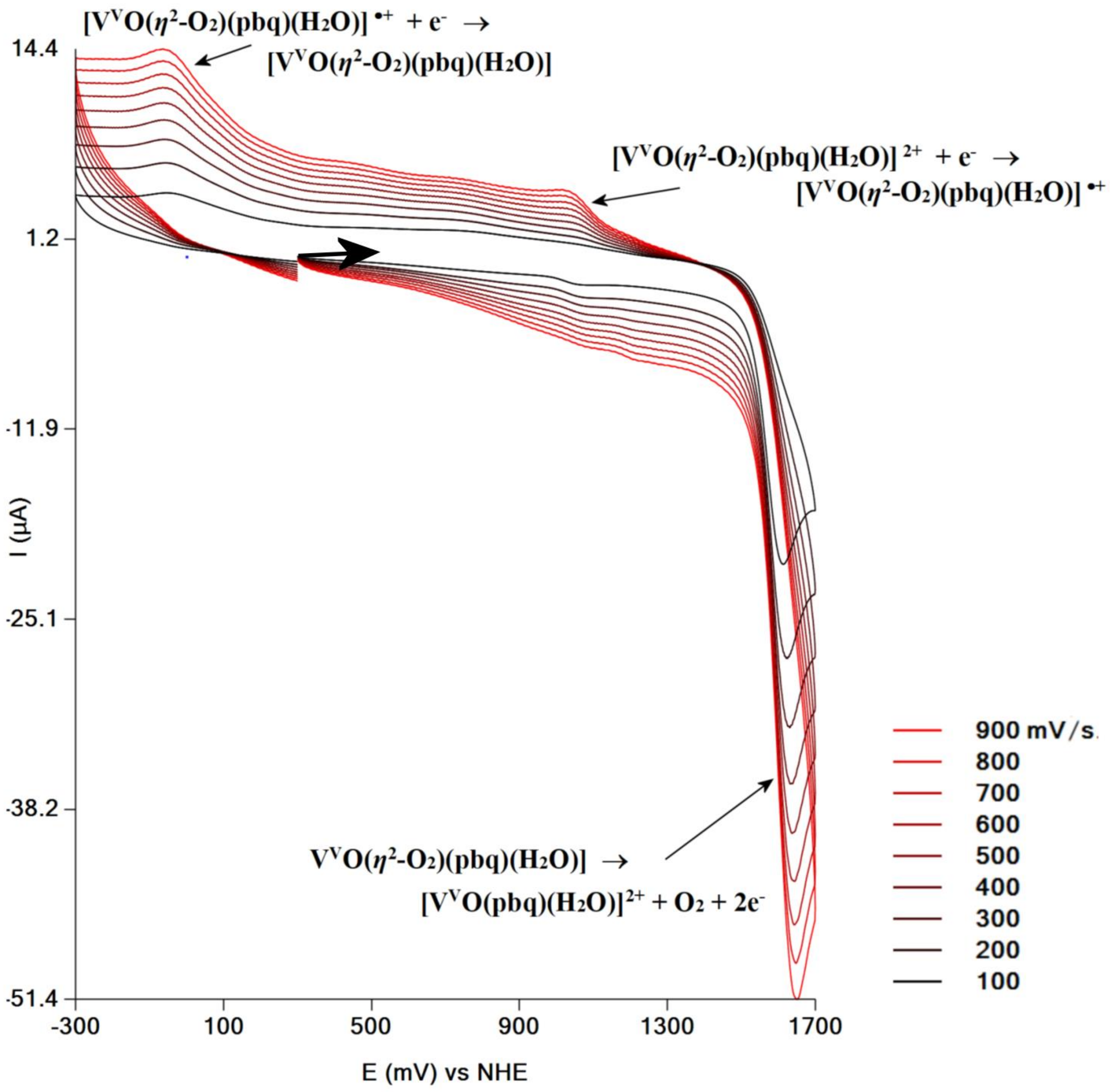


**Figure S17.** Cyclic voltammographs of $CH_3CN$ solution of [$V^VO(\eta^2-O_2)(pbq)(H_2O)$] at various scan rates. Working electrode: glassy carbon, Auxiliary electrode: platinum, Reference electrode: Ag/AgCl.

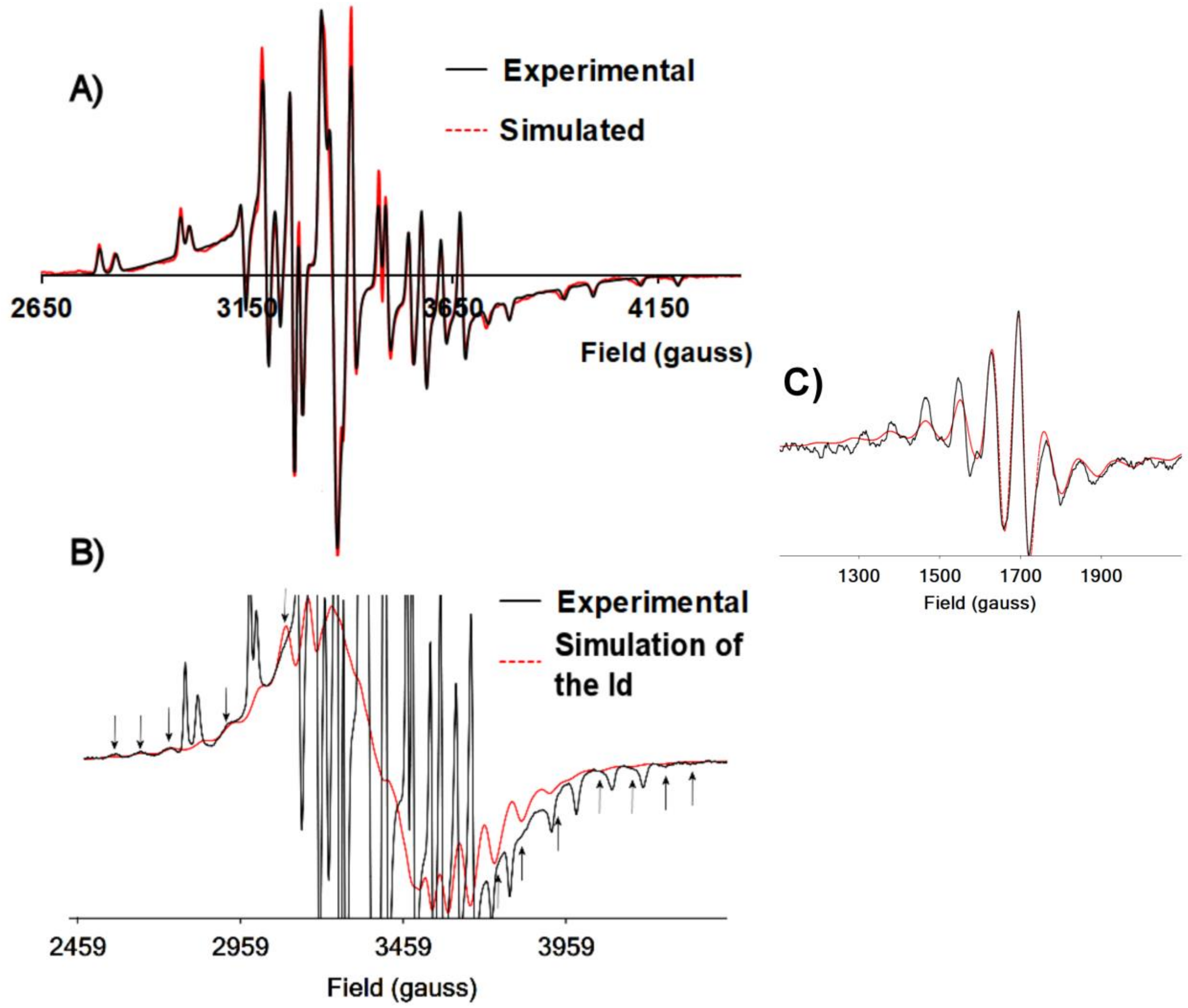


**Figure S18.** A) The X-band cw EPR spectrum of $V^{IV}(=O)^{2+}$-Hpbq - $O_2$ system in a frozen (120 K) solution (full black line) and the simulated spectrum (dotted red line) considering three paramagnetic vanadium complexes present in solution $[V^{IV}(=O)(H_2O)_5]^{2+}$, $[V^{IV}(=O)(pbq)(H_2O)_2]^{+}$ and **Id**. B) The spectra of the **Id** (red dotted line) and the experimental (full black line) reproduced from the simulation parameters using the following parameters for both vanadium centers $g_x$= 1.970 $g_y$=1.993 $g_z$=1.955 $A_x$=22 x $10^{-4}$ $cm^{-1}$ $A_y$=23 x $10^{-4}$ $cm^{-1}$ $A_z$=150 x $10^{-4}$ $cm^{-1}$ $A_x$'=9 x $10^{-4}$ $cm^{-1}$ $A_{y|}$'=5 x $10^{-4}$ $cm^{-1}$ $A_z$=9 x $10^{-4}$ $cm^{-1}$ and lwpp=2.0 mT, and 592 x $10^{-4}$, 247 x $10^{-4}$ and 241 x $10^{-4}$ $cm^{-1}$ isotropic, antisymmetric and symmetric electron–electron coupling. Arrows show the peaks used for the simulation. C) EPR signal at the half-field of the forbidden $\Delta M_s$=2 transition (black line) and simulation using the parameters of B (red line).

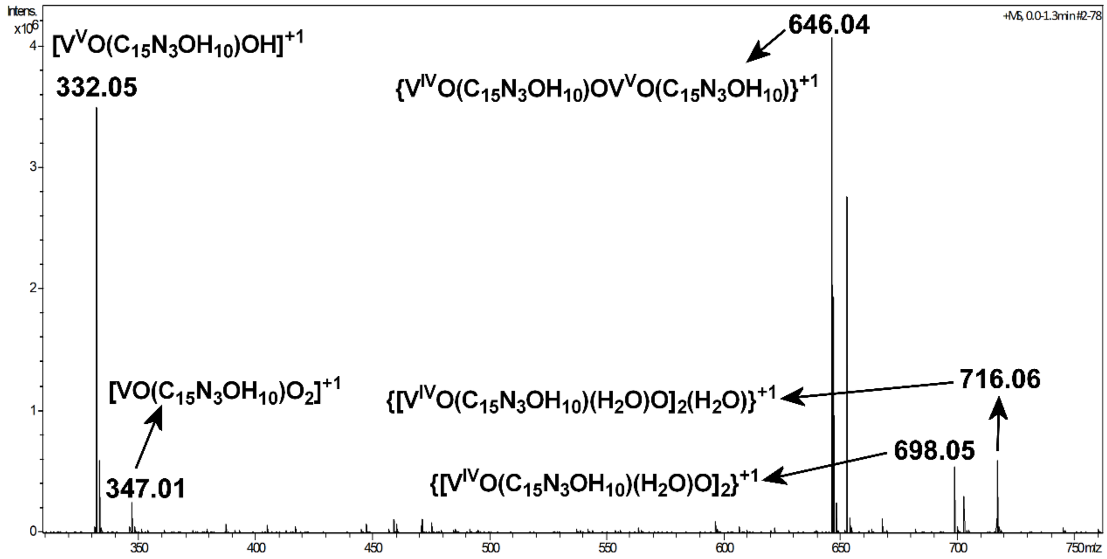


**Figure S19**. Positive ion mass spectrum of the $VOSO_4$ : $C_{15}H_{11}N_3O$ reaction mixture in $H_2O$:$CH_3OH$ (1:3) after 2 hrs of reaction time. The isotopic distribution envelopes correspond to singly charged species centred at 332.05, 347.01, 646.04, 698.05 and 716.06 m/z and can be attributed to the mono- and binuclear vanadium species.

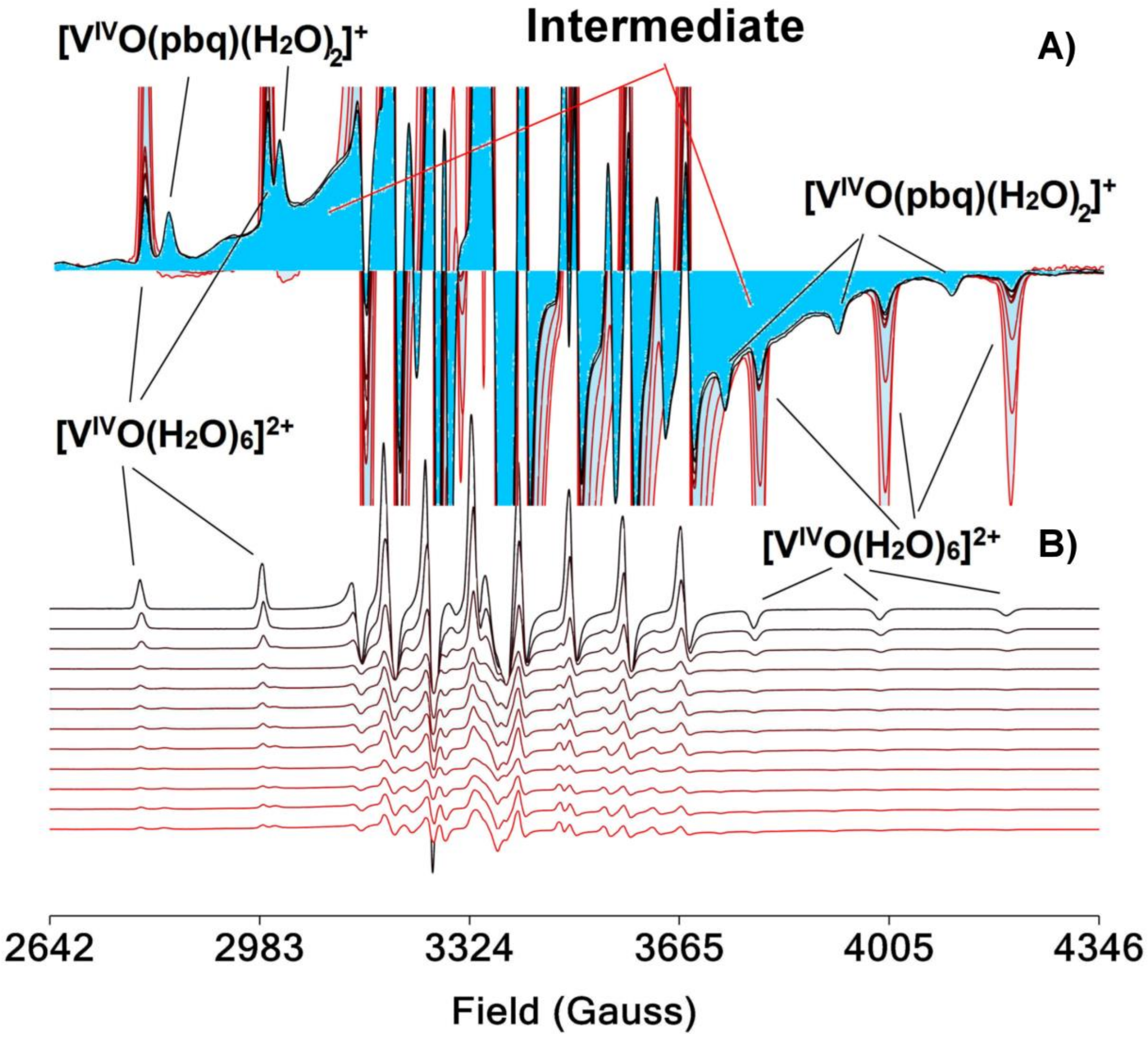


**Figure S20. A)** The X-band cw EPR spectra of $V^{IV}OSO_4{\cdot}3.5H_2O$ (0.0144 M)-Hpbq (0.0138 M) in solution *vs*. time, one spectrum every 20 min, and assignments of the three species, B) overlay of all spectra from 0 to 240 min.

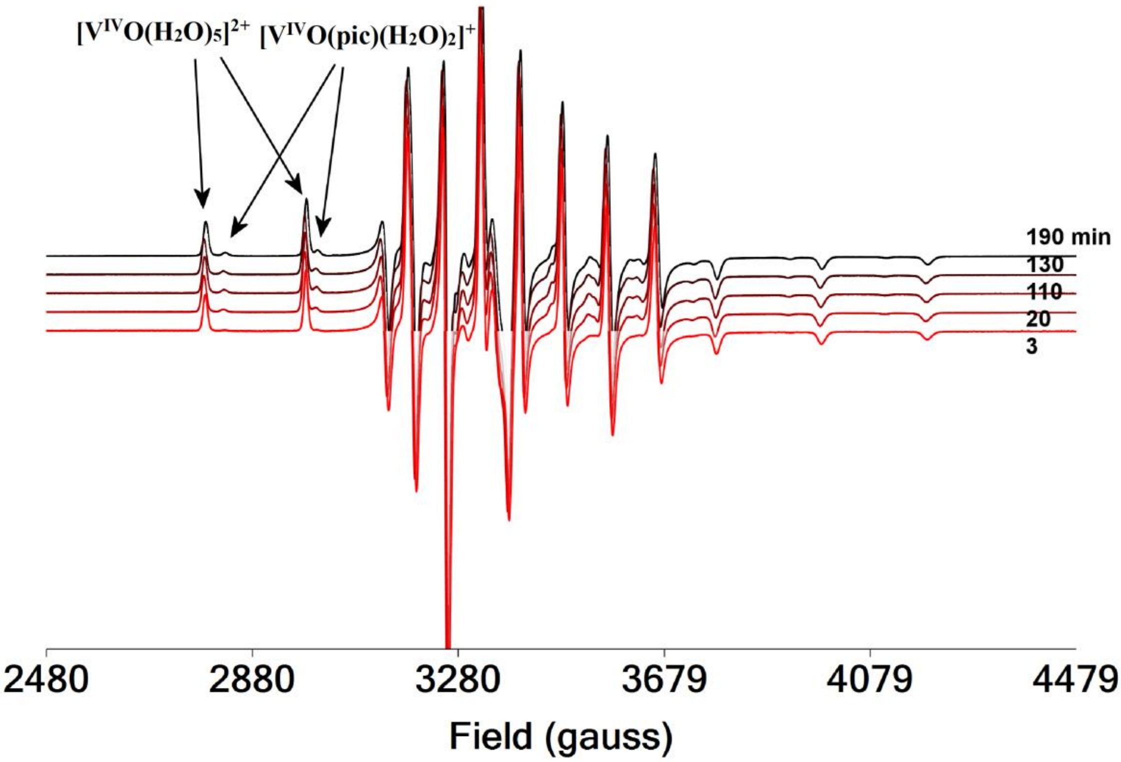


**Figure S21.** The X-band cw EPR spectra of $V^{IV}OSO_4 \cdot 3.5H_2O$ (0.0144 M)-Hpic (0.0138 M) in $H_2O$:$CH_3OH$ (25:75, v/v) frozen solutions vs time.

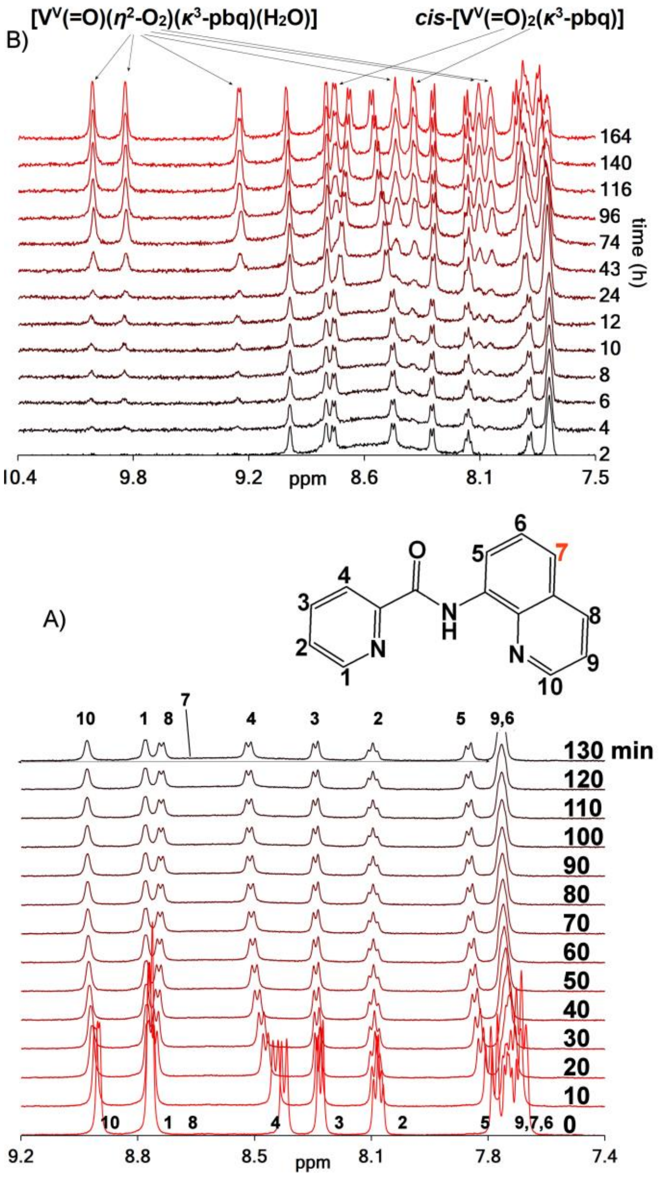


**Figure S22. (A)** $^1$H NMR spectra of a $V^{IV}OSO_4 \cdot 3.5H_2O$ (0.0144 M)-Hpbq (0.0138 M) in solution *vs*. time (first 130 min), and proton assignments. **(B)** $^1$H NMR spectra of a $V^{IV}OSO_4 \cdot 3.5H_2O$ (0.0144 M)-Hbpq (0.0138 M) in solution *vs*. time (first 7 days) and assignments of the peaks of compounds $[V^V(=O)(\eta^2\text{-}O_2)(\kappa^3\text{-pbq})(H_2O)]$ and *cis*-$[V^V(=O)_2(\kappa^3\text{-pbq})]$.

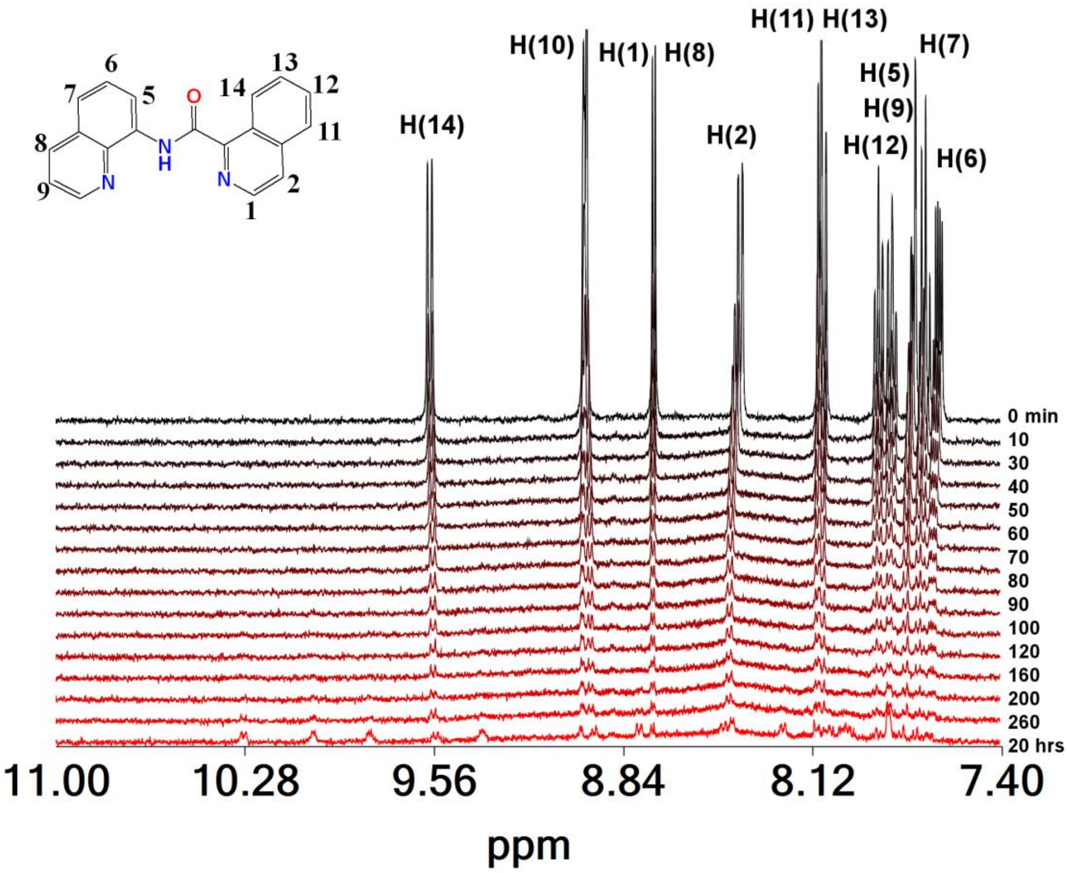


**Figure S23.** $^1$H NMR spectra of the reaction of the $D_2O$:$CH_3OH$ (25:75, v/v) solution of $V^{IV}OSO_4 \cdot 3.5H_2O$ (0.0144 M)-Hpyc (0.0138 M)- *vs* time (h) and assignments.

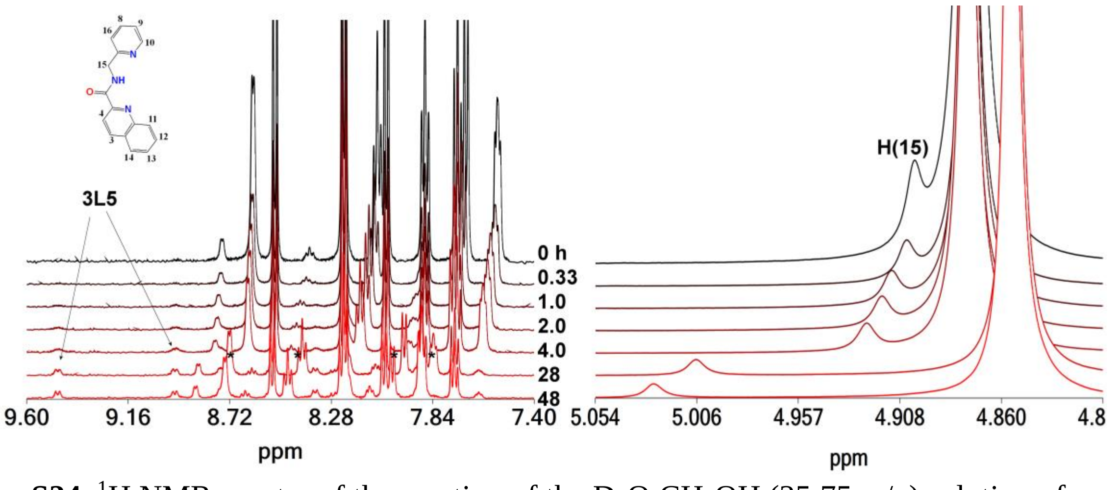


**Figure S24.** $^1$H NMR spectra of the reaction of the $D_2O$:$CH_3OH$ (25:75, v/v) solution of $V^{IV}OSO_4 \cdot 3.5H_2O$ (0.0144 M)-Hqqc (0.0138 M)- *vs* time (h) and assignments.

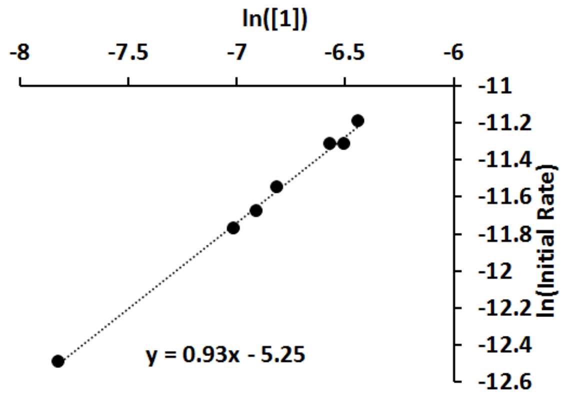


**Figure S25.** Graph of ln([**1**]) *vs* ln(Initial Rates) ([**1**] = 4.0 $10^{-4}$ – 1.6 $10^{-3}$ M) and linear fitting.

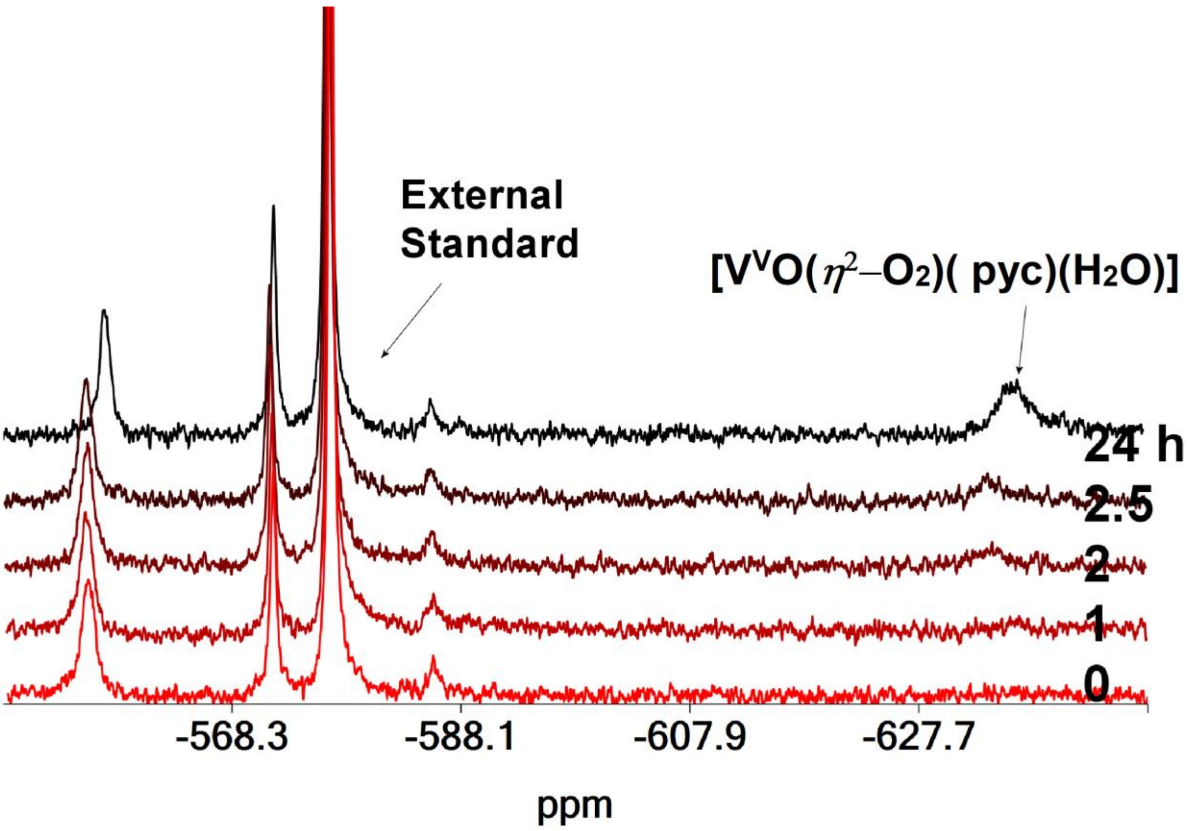


**Figure S26.** $^{51}$V NMR spectra of the reaction of the $D_2O$:$CH_3OH$ (25:75, v/v) solution of $V^{IV}OSO_4 \cdot 3.5H_2O$ (0.0144 M)-Hpyc (0.0138 M)- *vs* time (h) and assignments. The V1, V2, V4, V5 peaks are assigned to vanadates monomer, dimer, tetramer and pentamer respectively originated from the external aqueous $NaVO_3$ solution used as quantitative standard.

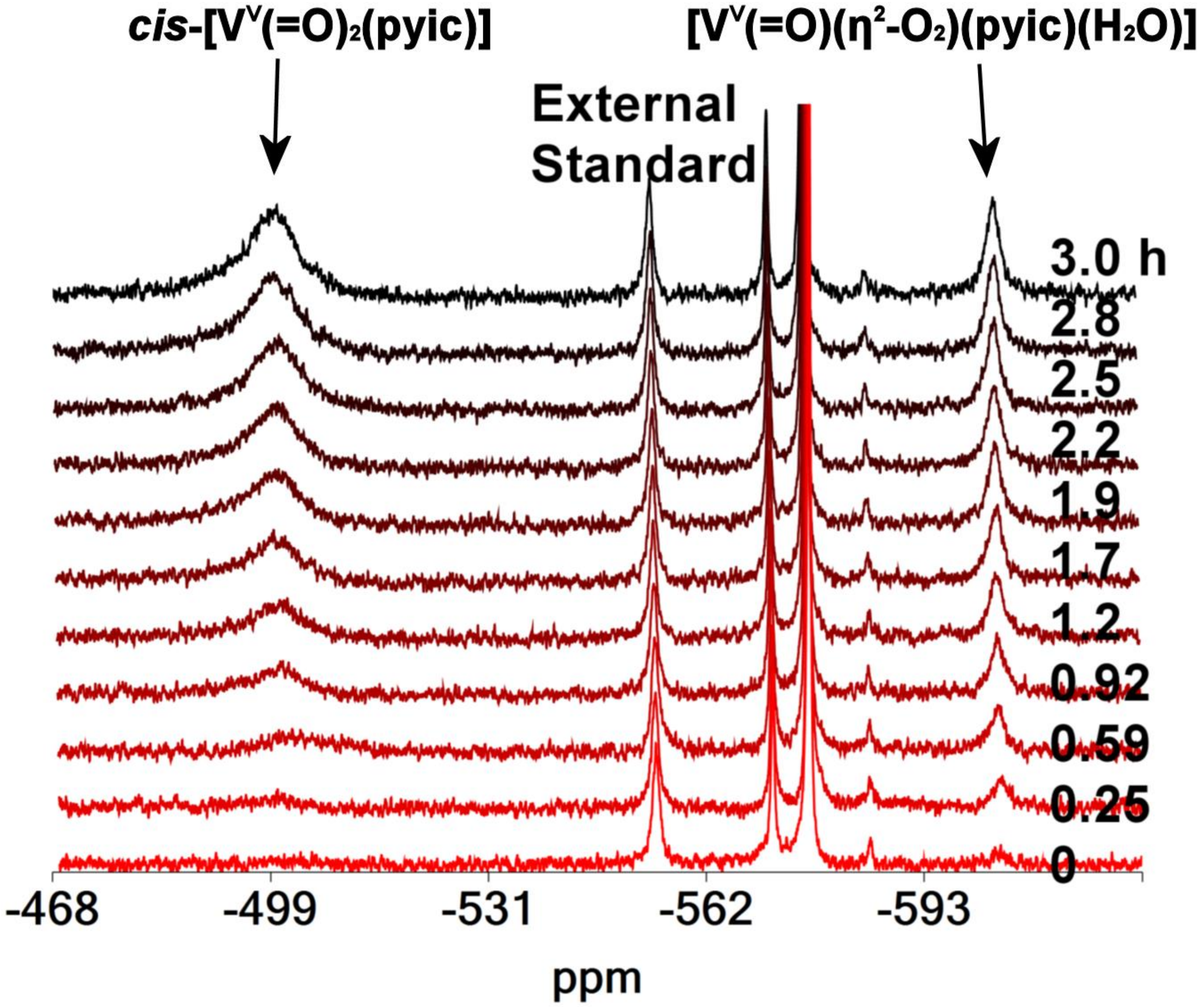


**Figure S27.** $^{51}V$ NMR spectra of the reaction of the $D_2O$:$CH_3OH$ (25:75, v/v) solution of $V^{IV}OSO_4 \cdot 3.5H_2O$ (0.0144 M)-Hpyic (0.0138 M)- *vs* time (h) and assignments. The V1, V2, V4, V5 peaks are assigned to vanadates monomer, dimer, tetramer and pentamer respectively originated from the external aqueous $NaVO_3$ solution used as quantitative standard.

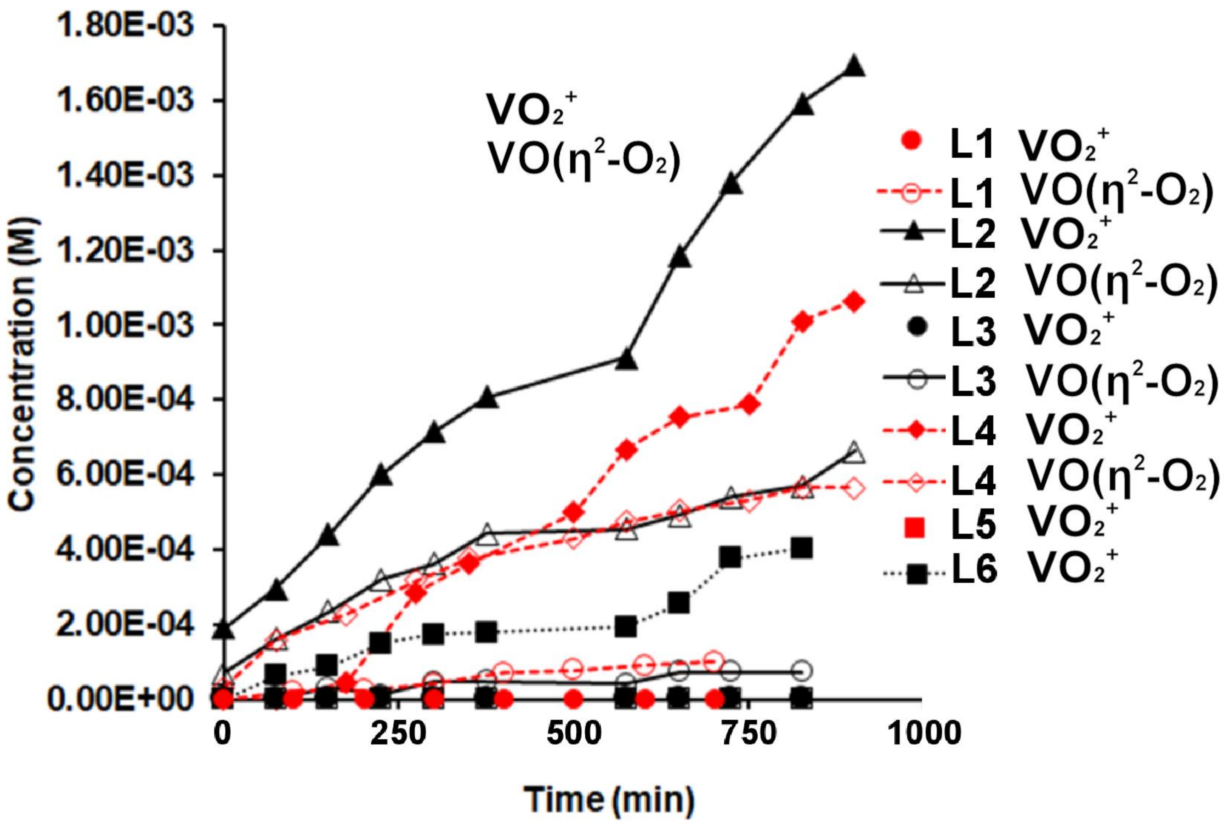


**Figure S28.** Concentration of **2L** and **3L** species *vs* time diagram of (25:75, v/v) $D_2O$:$CD_3OD$ solution of $V^{IV}OSO_4 \cdot 3.5H_2O$ (0.0144 M)-L (0.0138 M).

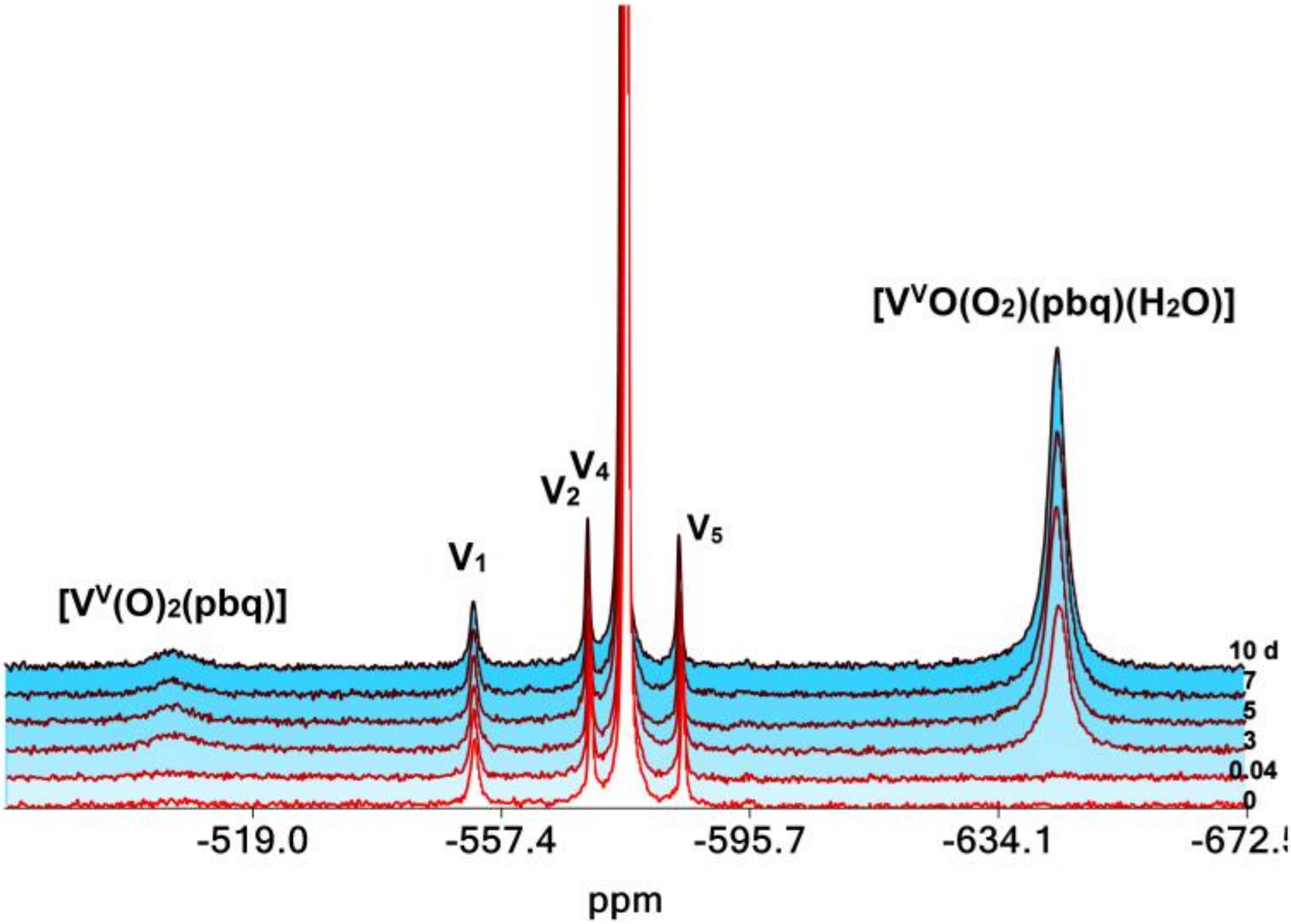


**Figure S29.** $^{51}V$ NMR spectra of the reaction of the $D_2O$:$CH_3OH$ (25:75, v/v) solution of $V^{IV}OSO_4 \cdot 3.5H_2O$ (0.0144 M)-Hpbq (0.0138 M)-hydroquinone (0.0138 M) with $O_2$ *vs* time (h) and assignments. The V1, V2, V4, V5 peaks are assigned to vanadates monomer, dimer, tetramer and pentamer respectively originated from the external aqueous $NaVO_3$ solution used as quantitative standard.

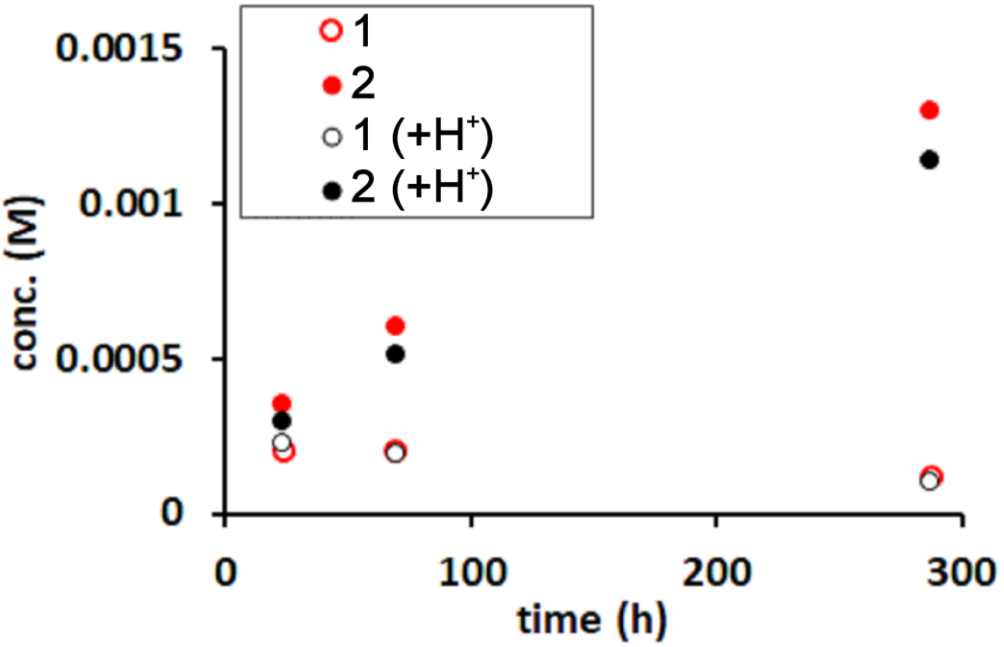


**Figure S30.** Graph showing the concentration of the vanadium species *vs* time of the $D_2O$:$CH_3OH$ (25:75, v/v) solution of a $V^{IV}OSO_4\cdot3.5H_2O$ (0.0144 M)-Hpbq (0.0138 M) a) without $H_2SO_4$ (red circles) and b) with $H_2SO_4$ (0.0050 M) (black circles). Species concentrations were calculated by integration of $^{51}V$ NMR peaks of the spectra. The filled shapes represent the concentration of **1**, empty shapes represent the concentration of **4**.

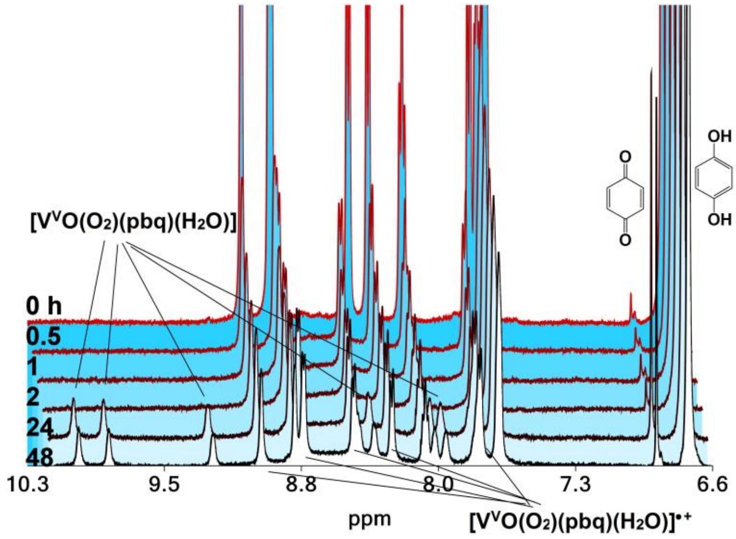


**Figure S31.** $^{1}$H NMR spectra of a (25:75, v/v) $D_2O$:$CD_3OD$ solution of $V^{IV}OSO_4 \cdot 3.5H_2O$ (0.0144 M)-Hpbq (0.0138 M)-hydroquinone (0.0138 M) vs time (first 7 days) and assignments.

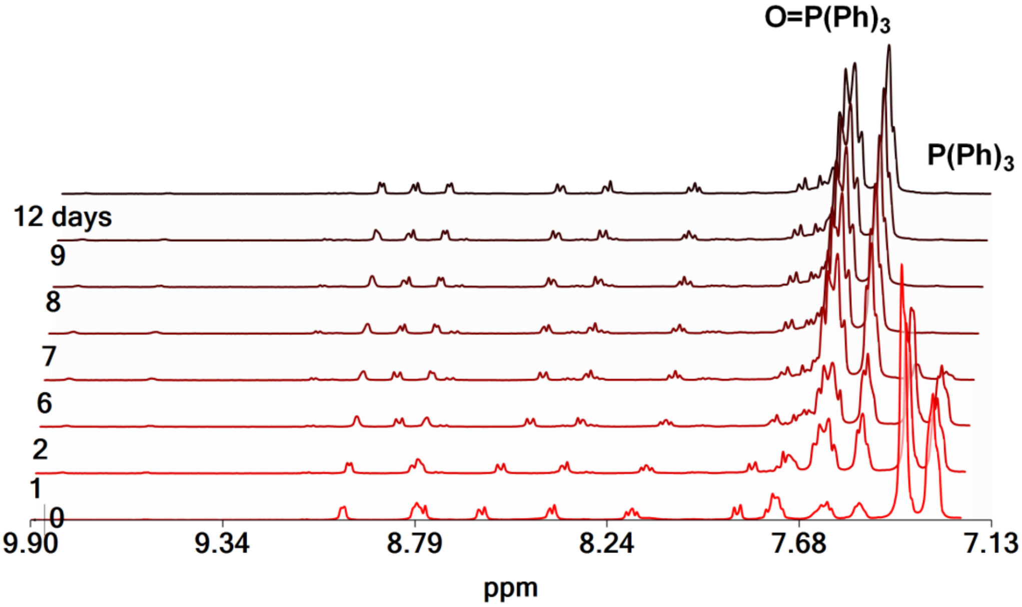


**Figure S32.** $^{1}$H NMR spectra of a (10:90, v/v) $D_2O$:$CD_3OD$ solution of $V^{IV}OSO_4 \cdot 3.5H_2O$ (0.0144 M)-Hpbq (0.0138 M)-triphenylphosphine (0.0276 M) vs time (first 12 days) and assignments.

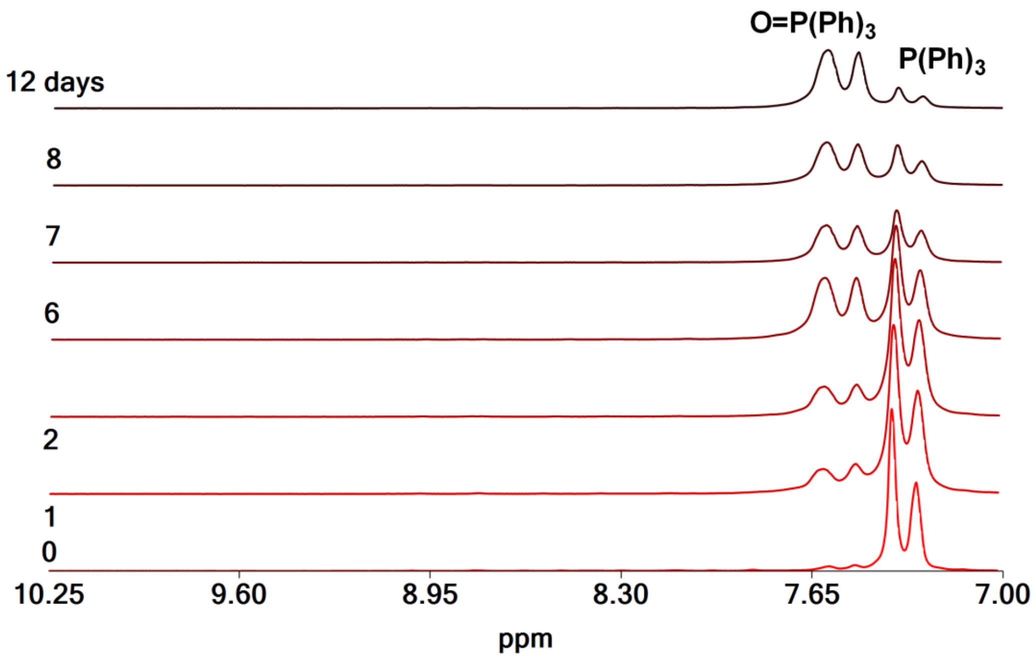


**Figure S33.** $^{1}H$ NMR spectra of a (10:90, v/v) $D_2O:CD_3OD$ solution of $V^{IV}OSO_4 \cdot 3.5H_2O$ (0.0144 M)-Hpbq (0.0138 M)-triphenylphosphine (0.276 M) vs time (first 12 days) and assignments.

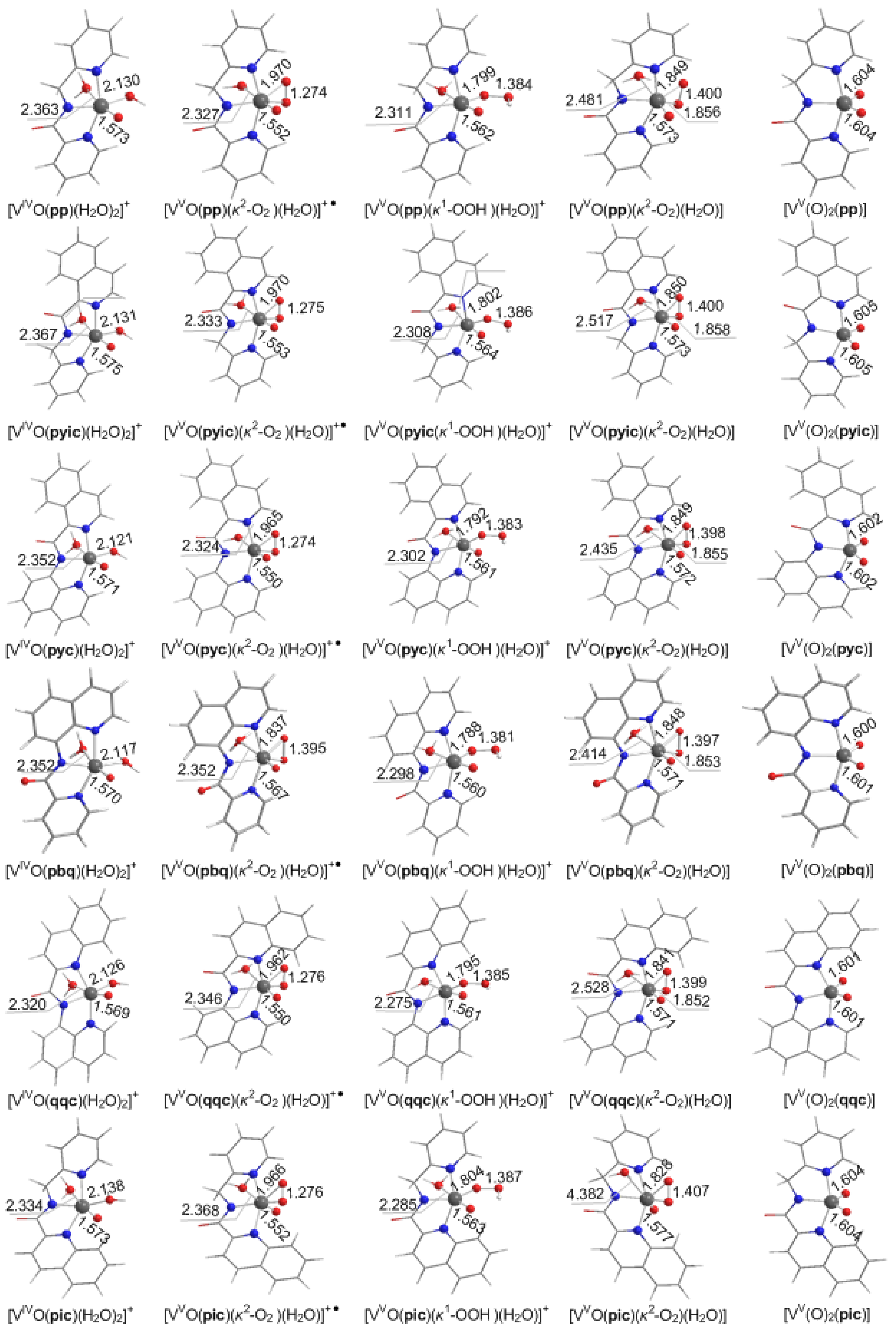


**Figure S34**. Geometric reaction profiles of the reductive activation of $O_2$ to $O_2^{2-}$ by the other $[V^{IV}O(L)(OH_2)_2]^+$ (L = pp, pyic, pyc, pbq, qqc, pic) complexes with selected structural parameters (bond lengths in Å) calculated at the PBE0/Def2-TZVP(V) ∪ 6-31+G(d)(E) level of theory in aqueous solution.

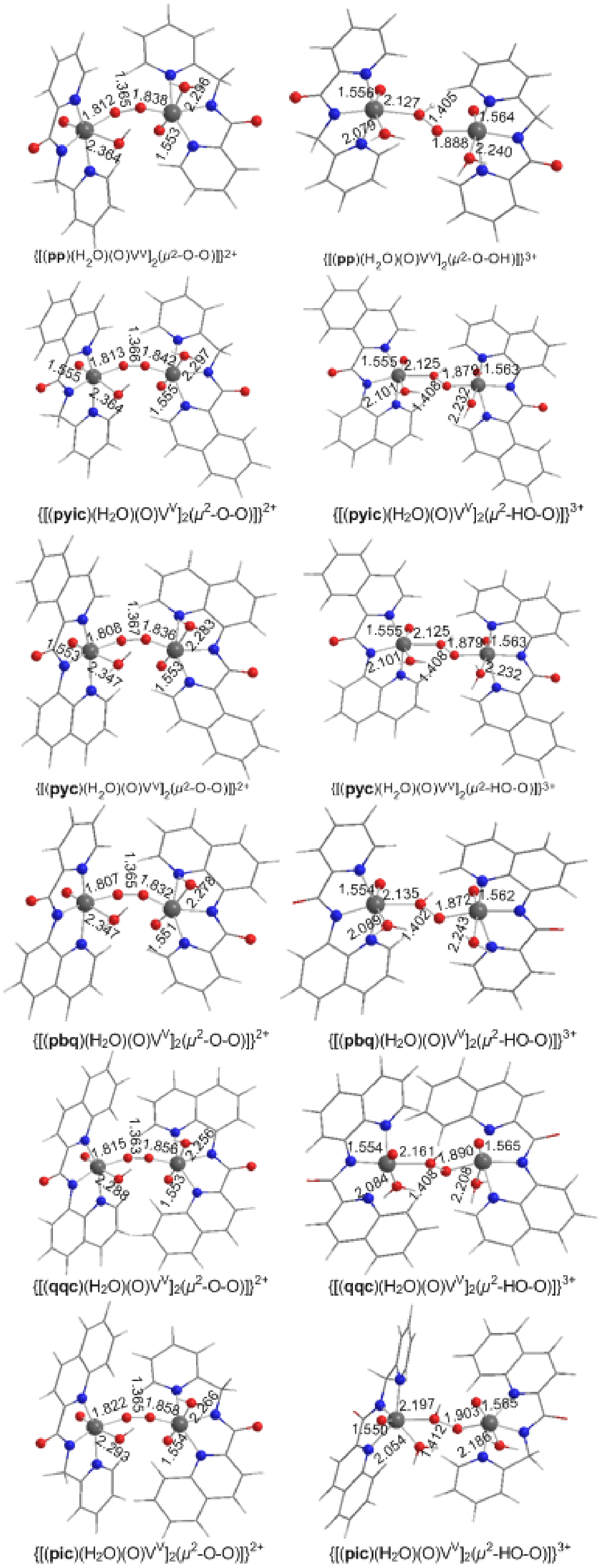


**Figure S35**. Optimized geometries of the peroxo-bridged dinuclear $\{[(L)(H_2O)(O)V]_2(\mu^2\text{-}\kappa^1,\kappa^1\text{-O-O})\}^{2+}$ and their monoprotonated intermediates with selected structural parameters (bond lengths in Å) calculated at the PBE0/Def2-TZVP(V) ◡ 6-31+G(d)(E) level of theory in aqueous solution.

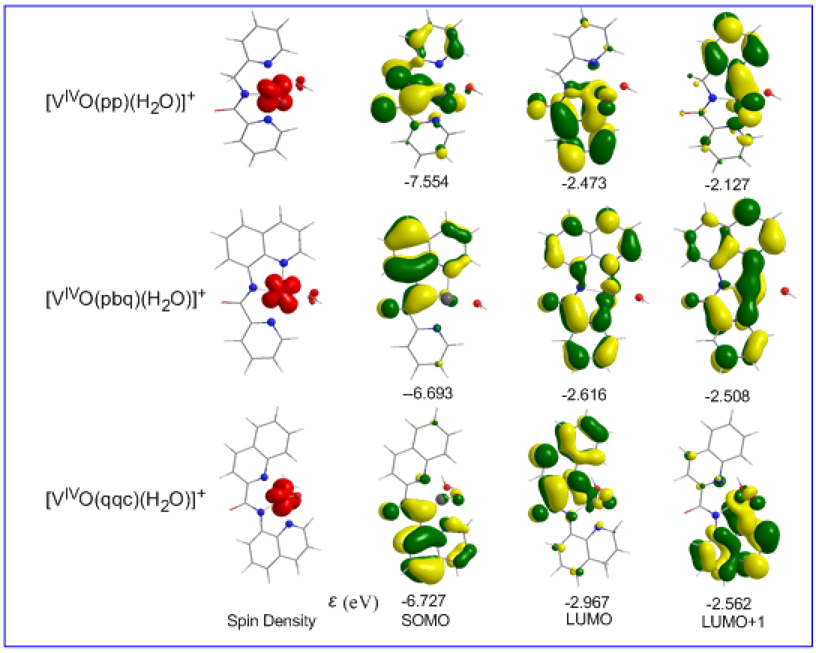


**Figure S36**. 3D-plots of the spin density distribution and frontier molecular orbitals of representative $[V^{IV}O(L)(OH_2)]^+$ complexes calculated at the PBE0/Def2-TZVP(V)∪6-31+G(d)(E) level of theory in aqueous solution.

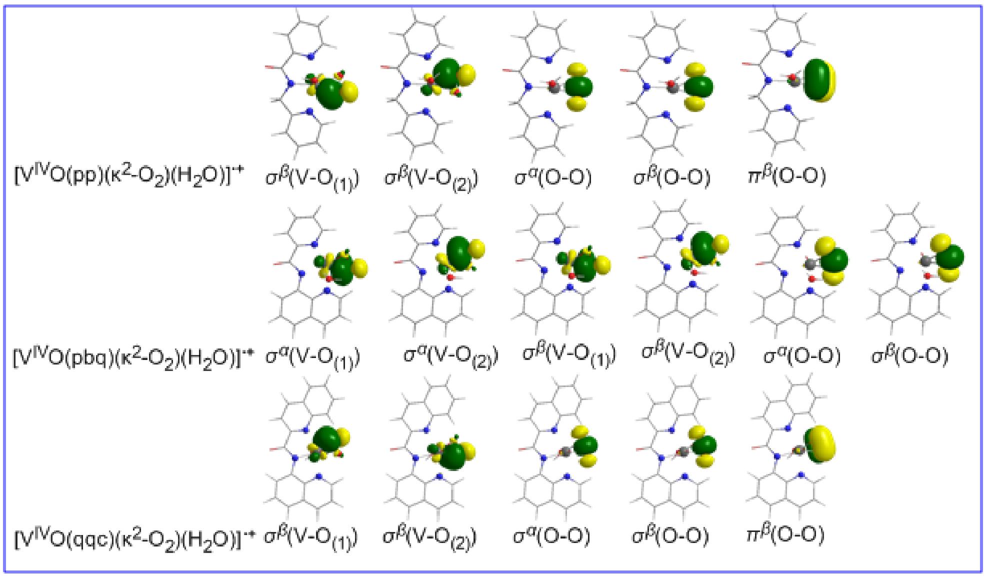


**Figure S37**. 3D-plots of the *BD*(V-O) and *BD*(O-O) NBOs of repreentative $[V^VO(L)(\kappa^2\text{-}O_2)(OH_2)]^{+\bullet}$ complexes calculated at the PBE0/Def2-TZVP(V)∪6-31+G(d)(E) level of theory in aqueous solution.

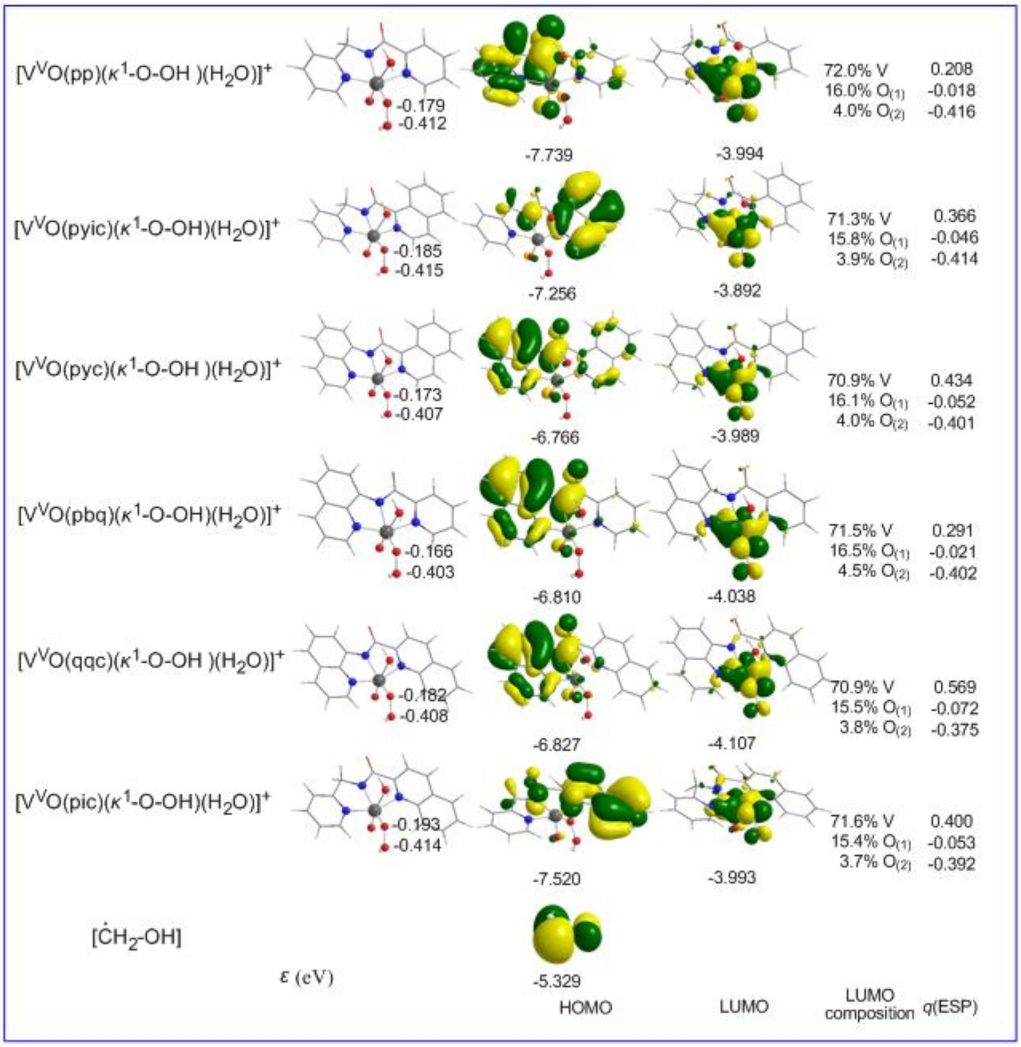


**Figure S38**. 3D-plots of the frontier molecular orbitals, LUMO composition and electrostatic potential fitting atomic charges, q(ESP), on V, proximal $O_{(1)}$ and distal $O_{(2)}$ atoms of the $[V^{V}O(pbq)(\kappa^{1}\text{-}OOH)(OH_2)]^{+}$ complexes calculated at the PBE0/Def2-TZVP(V)∪6-31+G(d)(E) level of theory in aqueous solution.

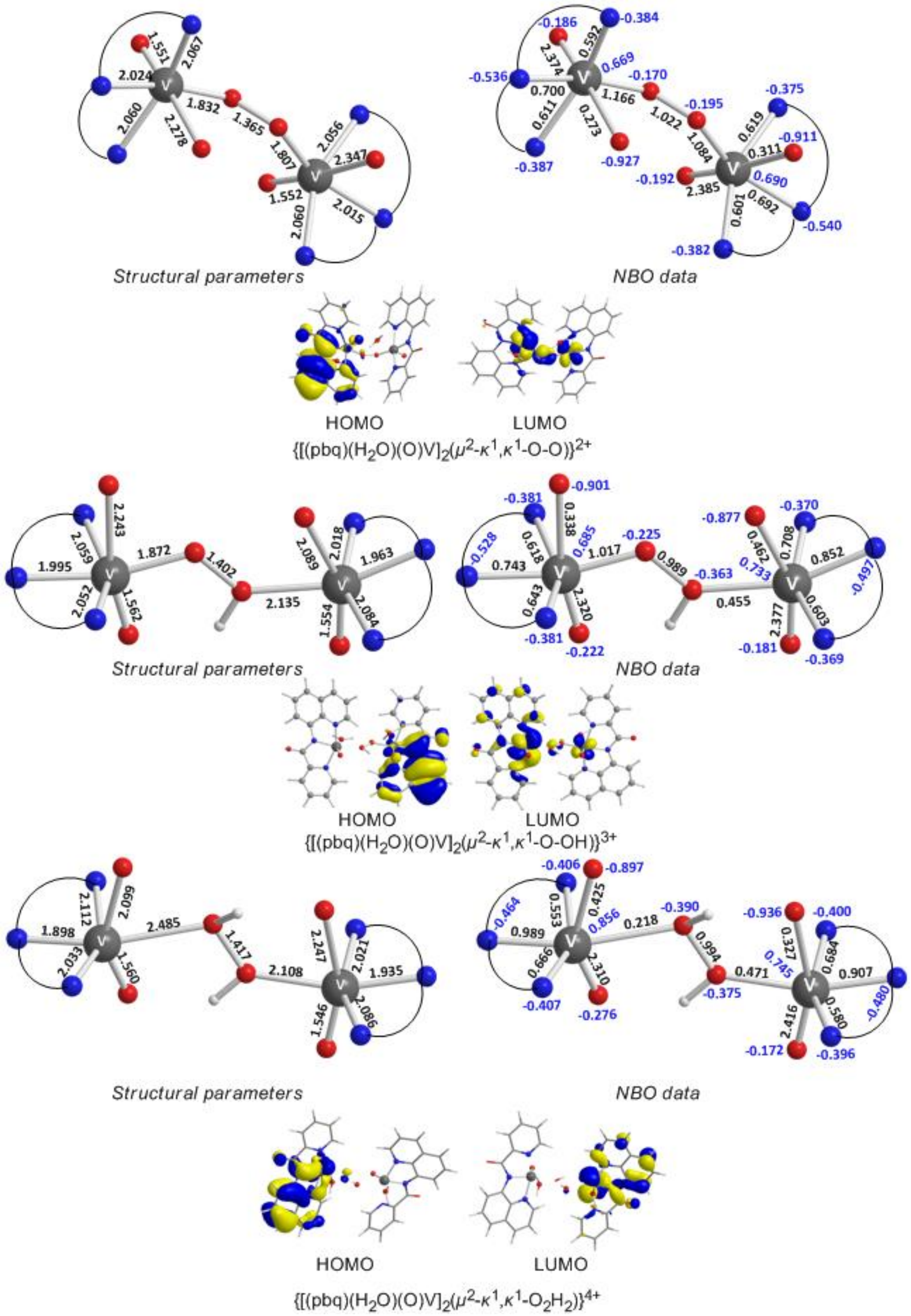


**Figure S39**. Equilibrium geometries, natural atomic charges (in blue), *WBO*s and 3D plots of FMOs of intermediates calculated by the PBE0/Def2-TZVP(V)$\cup$6-31+G(d)(E)/PCM computational protocol in aqueous solution.

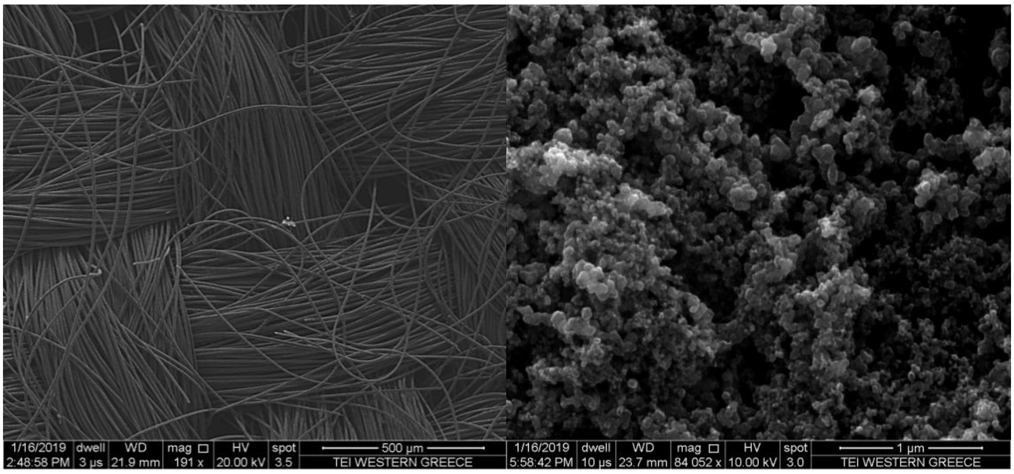


**Figure S40.** SEM images of the structure of the carbon cloth electrode (left) and the mesoporous carbon film (right)

**Table S1.** Crystallographic and experimental data for **1**·$H_2O$,**1'**and **2.**[a]

| parameters | **1**·$H_2O$ | **1'** | **2** |
|---|---|---|---|
| Empiricalformula | $C_{15}$ $H_{14}$ $N_3$ $O_6$ V | $C_{16}$ $H_{14}$ $N_3$ $O_5$ V | $C_{15}$ $H_{10}$ $N_3$ O3 V |
| Formulaweight | 383.23 | 379.24 | 331.20 |
| Temperature | 100(2) K | 100(2) K | 100(2) K |
| Wavelength | 1.54184 Å | 1.54184 Å | 1.54184 Å |
| Crystsyst | Monoclinic | Monoclinic | Monoclinic |
| Spacegroup | *P*21/*n* | *P*21/*c* | *P*21/c |
| *a*(Å) | 8.2766 (2) | 8.8472(6) | 15.2572 (3) |
| *b*(Å) | 10.8579 (3) | 7.4074(5) | 12.5438 (2) |
| *c*(Å) | 16.8325 (5) | 24.0139(16) | 6.9639 (1) |
| $\alpha$(deg) | 90 | 90 | 90 |
| β(deg) | 90.732(3) | 99.558(7) | 103.025 (2) |
| γ(deg) | 90 | 90 | 90 |
| Vol.($Å^3$) | 1512.55(7) | 1551.90(18) | 1298.48 (4) |
| *Z* | 4 | 4 | 4 |
| $D_{calcd}$ ($Mg/m^3$) | 1.683 | 1.623 | 1.694 |
| Abscoeff ($mm^{-1}$) | 5.871 | 5.667 | 6.557 |
| Θ range for data collection (deg) | 4.847-74.730 | 5.069 - 74.162 | 2.963-76.775 |
| Index ranges | -9<=h<=10<br>-12<=k<=13<br>-20<=l<=20 | -8<=h<=10<br>-8<=k<=5<br>-29<=l<=29 | -19<=h<=18<br>-12<=k<=15<br>-8<=l<=8 |
| Collected | 9699 | 5151 | 8885 |
| Unique | 3030 | 3022 | 2630 |
| Rint | 0.0660 | 0.0369 | 0.0366 |
| data/restraints/parameters | 3030 / 0 / 242 | 3022 / 0 / 242 | 2630 / 0 / 199 |
| GOF on $F^2$ | 1.043 | 1.031 | 1.068 |
| Max/min□*p* ($eÅ^{-3}$) | 0.433 and -0.533 | 0.948 and -0.533 | 1.096 and -0.519 |
| Final *R* indices *(R*, $R_w$) [I>2σ(I)] | 0.0500<br>0.1298 | 0.0609<br>0.1672 | 0.0478<br>0.1311 |
| *R*, w*R* (alldata) | 0.0597<br>0.1396 | 0.0642<br>0.1712 | 0.0499<br>0.1332 |

[a]$R = \Sigma||F_o| - |F_c||/\Sigma|F_o|$, $wR = [\Sigma w(|F_o|^2 - |F_c|^2)/\Sigma w|F_o|^2]^{1/2}$, GOF = $[\Sigma[w(F_o^2 - F_c^2)^2]/(n-p)]^{1/2}$, $w = 1/[\sigma^2(F_o^2) + (aP)^2 + bP]$, where$P = (F_o^2 + 2F_c^2)/3$.

**Table S2.** Crystallographic and experimental data for **4** and **5.** [a]

| parameters | **4** | **5** |
|---|---|---|
| Empiricalformula | C16 H12 N3 O3 V | C16 H12 N3 O3 V |
| Formulaweight | 345.23 | 345.23 |
| Temperature | 100(2) K | 100(2) K |

| | | |
|---|---|---|
| Wavelength | 1.54184 Å | 0.71073 Å |
| Crystsyst | Monoclinic | Monoclinic |
| Spacegroup | *P*21/*c* | *P*21/*n* |
| *a*(Å) | 14.2018(10) | 6.9305(5) |
| *b*(Å) | 14.5305(11) | 14.1020(12) |
| *c*(Å) | 6.9060(6) | 14.2356(12) |
| □(deg) | 90 | 90 |
| □(deg) | 97.637(8) | 94.012(7) |
| □(deg) | 90 | 90 |
| Vol.($Å^3$) | 1412.48(19) | 1387.89(19) |
| *Z* | 4 | 4 |
| $□_{calcd}$ (Mg/□□) | 1.633 | 1.652 |
| Abscoeff ($mm^{-1}$) | 6.064 | 0.734 |
| □range for data collection (deg) | 4.373 to 72.414 | 3.282 to 29.543 |
| Indexranges | -15<=h<=17, -16<=k<=17, -7<=l<=8 | -8<=h<=9, -19<=k<=16, -14<=l<=18 |
| Collected | 5154 | 8026 |
| Unique | 2685 | 3334 |
| Rint | 0.0633 | 0.0265 |
| data/restraints/parameters | 2685 / 0 / 210 | 3334 / 0 / 256 |
| GOF on $F^2$ | 1.215 | 1.054 |
| Max/min□*p* ($eÅ^{-3}$) | 2.976 and -0.811 | 0.931 and -0.576 |
| Final *R* indices (*R*, $R_w$) [I>2□□I)] | 0.1080<br>0.2820 | 0.0425<br>0.1036 |
| *R*, w*R*(alldata) | 0.1177<br>0.2961 | 0.0533<br>0.1113 |

[a] $R = \Sigma||F_o| - |F_c||/\Sigma|F_o|$, $wR = [\Sigma w(|F_o|^2 - |F_c|^2)/\Sigma w|F_o|^2]^{1/2}$, GOF = $[\Sigma[w(F_o^2 - F_c^2)^2]/(n - p)]^{1/2}$, $w = 1/[\sigma^2(F_o^2) + (aP)^2 + bP]$, where$P = (F_o^2 + 2F_c^2)/3$.

**Table S3.** Crystallographic and experimental data for **6** and **9**.MeOH. [a]

| parameters | **6** | **9**.MeOH |
|---|---|---|
| Empiricalformula | C38 H24 N6 O6 V2 | C16 H15 Cl N3 O3 V |
| Formulaweight | 762.51 | 383.70 |
| Temperature | 100(2) K | 100(2) K |
| Wavelength | 0.71073 Å | 1.54184 Å |
| Crystsyst | Monoclinic | Orthorhombic |
| Spacegroup | *P*21/*c* | *Pbca* |
| *a*(Å) | 15.832(2) | 7.4075(2) |
| *b*(Å) | 15.5035(10) | 16.3749(5) |
| *c*(Å) | 12.6820(12) | 26.1694(8) |

| | | |
|---|---|---|
| □(deg) | 90 | 90 |
| □(deg) | 100.976(10) | 90 |
| □(deg) | 90 | 90 |
| Vol.(Å$^3$) | 3055.9(5) | 3174.27(16) |
| *Z* | 4 | 8 |
| □$_{calcd}$ (Mg/□$^□$) | 1.657 | 1.606 |
| Abscoeff (mm$^{-1}$) | 0.676 | 6.961 |
| □range for data collection (deg) | 3.286 to 29.705 | 3.378 to 73.075 |
| Indexranges | -19<=h<=20, -20<=k<=17, -17<=l<=16 | -8<=h<=6, -19<=k<=18, -30<=l<=32 |
| Collected | 19878 | 6733 |
| Unique | 7404 | 3086 |
| Rint | 0.0285 | 0.0332 |
| data/restraints/parameters | 7404 / 0 / 469 | 3086 / 3 / 221 |
| GOF on $F^2$ | 1.081 | 1.054 |
| Max/min□*p* (eÅ$^{-3}$) | 2.010 and -0.472 | 0.916 and -0.535 |
| Final *R* indices *(R*, $R_w$*)* [I>2□□I)] | 0.0564 0.1464 | 0.0422 0.1100 |
| *R*, w*R*(alldata) | 0.1464 0.1612 | 0.0515 0.1159 |

$^a R = \Sigma||F_o| - |F_c||/\Sigma|F_o|$, $wR = [\Sigma w(|F_o|^2 - |F_c|^2)/\Sigma w|F_o|^2]^{1/2}$, GOF = $[\Sigma[w(F_o^2 - F_c^2)^2]/(n - p)]^{1/2}$, $w = 1/[\sigma^2(F_o^2) + (aP)^2 + bP]$, where $P = (F_o^2 + 2F_c^2)/3$.

**Table S4**. Bond lengths [Å] and angles [°] for **1**.

| | |
|---|---|
| V(1)-O(1) | 1.599(2) |
| V(1)-O(3) | 1.888(2) |
| V(1)-O(2) | 1.888(2) |
| V(1)-N(2) | 2.101(2) |
| V(1)-N(3) | 2.134(2) |
| V(1)-N(1) | 2.145(2) |
| V(1)-O(4) | 2.243(2) |
| O(2)-O(3) | 1.424(3) |
| N(2)-C(6) | 1.327(4) |
| N(2)-C(7) | 1.412(4) |
| O(5)-C(6) | 1.240(4) |
| N(1)-C(1) | 1.334(4) |
| N(1)-C(5) | 1.342(4) |
| N(3)-C(13) | 1.327(4) |
| N(3)-C(14) | 1.365(4) |
| C(6)-C(5) | 1.497(4) |
| C(13)-C(12) | 1.408(4) |
| C(12)-C(11) | 1.369(4) |
| C(11)-C(15) | 1.404(4) |
| C(7)-C(8) | 1.388(4) |
| C(7)-C(14) | 1.416(4) |
| C(4)-C(3) | 1.378(5) |
| C(4)-C(5) | 1.396(4) |
| C(8)-C(9) | 1.421(5) |
| C(15)-C(10) | 1.414(4) |
| C(15)-C(14) | 1.416(4) |
| C(10)-C(9) | 1.371(5) |
| C(1)-C(2) | 1.388(4) |
| C(2)-C(3) | 1.393(5) |
| | |
| O(1)-V(1)-O(3) | 101.91(10) |
| O(1)-V(1)-O(2) | 102.15(10) |
| O(3)-V(1)-O(2) | 44.30(9) |
| O(1)-V(1)-N(2) | 95.81(10) |
| O(3)-V(1)-N(2) | 150.30(9) |
| O(2)-V(1)-N(2) | 152.17(10) |
| O(1)-V(1)-N(3) | 94.74(10) |
| O(3)-V(1)-N(3) | 125.72(9) |
| O(2)-V(1)-N(3) | 81.87(9) |
| N(2)-V(1)-N(3) | 75.56(9) |
| O(1)-V(1)-N(1) | 92.91(10) |
| O(3)-V(1)-N(1) | 80.51(10) |
| O(2)-V(1)-N(1) | 124.53(10) |
| N(2)-V(1)-N(1) | 74.92(10) |
| N(3)-V(1)-N(1) | 150.08(10) |
| O(1)-V(1)-O(4) | 172.78(9) |
| O(3)-V(1)-O(4) | 84.20(9) |
| O(2)-V(1)-O(4) | 84.90(9) |
| N(2)-V(1)-O(4) | 77.05(8) |
| N(3)-V(1)-O(4) | 84.60(8) |
| N(1)-V(1)-O(4) | 84.24(8) |
| O(3)-O(2)-V(1) | 67.83(12) |
| C(6)-N(2)-C(7) | 121.6(2) |
| C(6)-N(2)-V(1) | 120.7(2) |
| C(7)-N(2)-V(1) | 117.69(19) |
| O(2)-O(3)-V(1) | 67.87(12) |
| C(1)-N(1)-C(5) | 119.0(3) |
| C(1)-N(1)-V(1) | 125.1(2) |

| | |
|---|---|
| C(5)-N(1)-V(1) | 115.64(19) |
| C(13)-N(3)-C(14) | 118.7(2) |
| C(13)-N(3)-V(1) | 124.8(2) |
| C(14)-N(3)-V(1) | 116.50(19) |
| O(5)-C(6)-N(2) | 129.1(3) |
| O(5)-C(6)-C(5) | 119.4(3) |
| N(2)-C(6)-C(5) | 111.5(3) |
| N(3)-C(13)-C(12) | 122.7(3) |
| C(11)-C(12)-C(13) | 119.3(3) |
| C(12)-C(11)-C(15) | 119.5(3) |
| C(8)-C(7)-N(2) | 127.8(3) |
| C(8)-C(7)-C(14) | 118.8(3) |
| N(2)-C(7)-C(14) | 113.4(2) |
| C(3)-C(4)-C(5) | 118.4(3) |
| C(7)-C(8)-C(9) | 119.5(3) |
| N(1)-C(5)-C(4) | 122.3(3) |
| N(1)-C(5)-C(6) | 116.5(3) |
| C(4)-C(5)-C(6) | 121.2(3) |
| C(11)-C(15)-C(10) | 123.1(3) |
| C(11)-C(15)-C(14) | 117.9(3) |
| C(10)-C(15)-C(14) | 118.9(3) |
| C(9)-C(10)-C(15) | 119.3(3) |
| N(1)-C(1)-C(2) | 122.3(3) |
| C(10)-C(9)-C(8) | 122.2(3) |
| N(3)-C(14)-C(7) | 116.8(2) |
| N(3)-C(14)-C(15) | 121.9(3) |
| C(7)-C(14)-C(15) | 121.3(3) |
| C(1)-C(2)-C(3) | 118.6(3) |
| C(4)-C(3)-C(2) | 119.4(3) |

Symmetry transformations used to generate equivalent atoms:

**Table S5**. Bond lengths [Å] and angles [°] for **1’**.

| | |
|---|---|
| V(1)-O(1) | 1.599(2) |
| V(1)-O(2) | 1.875(2) |
| V(1)-O(3) | 1.887(2) |
| V(1)-N(2) | 2.093(2) |
| V(1)-N(3) | 2.125(2) |
| V(1)-N(1) | 2.138(2) |
| V(1)-O(4) | 2.275(2) |
| O(3)-O(2) | 1.422(3) |
| O(5)-C(6) | 1.231(3) |
| O(4)-C(1M) | 1.431(4) |
| N(2)-C(6) | 1.348(3) |
| N(2)-C(7) | 1.400(3) |
| N(1)-C(1) | 1.336(4) |
| N(1)-C(5) | 1.347(4) |
| N(3)-C(13) | 1.328(4) |
| N(3)-C(14) | 1.368(4) |
| C(5)-C(4) | 1.384(4) |
| C(5)-C(6) | 1.499(4) |
| C(7)-C(8) | 1.384(4) |
| C(7)-C(14) | 1.427(4) |
| C(14)-C(15) | 1.415(4) |
| C(10)-C(9) | 1.370(4) |
| C(10)-C(15) | 1.415(4) |
| C(12)-C(11) | 1.364(5) |
| C(12)-C(13) | 1.398(4) |

| | |
|---|---|
| C(3)-C(2) | 1.380(4) |
| C(3)-C(4) | 1.388(4) |
| C(8)-C(9) | 1.409(4) |
| C(2)-C(1) | 1.382(4) |
| C(15)-C(11) | 1.413(4) |
| | |
| O(1)-V(1)-O(2) | 103.06(10) |
| O(1)-V(1)-O(3) | 102.46(9) |
| O(2)-V(1)-O(3) | 44.41(9) |
| O(1)-V(1)-N(2) | 95.73(9) |
| O(2)-V(1)-N(2) | 150.06(9) |
| O(3)-V(1)-N(2) | 151.46(9) |
| O(1)-V(1)-N(3) | 93.41(9) |
| O(2)-V(1)-N(3) | 125.35(9) |
| O(3)-V(1)-N(3) | 81.37(9) |
| N(2)-V(1)-N(3) | 75.71(8) |
| O(1)-V(1)-N(1) | 95.67(9) |
| O(2)-V(1)-N(1) | 80.58(9) |
| O(3)-V(1)-N(1) | 124.49(9) |
| N(2)-V(1)-N(1) | 74.48(9) |
| N(3)-V(1)-N(1) | 149.53(9) |
| O(1)-V(1)-O(4) | 172.12(9) |
| O(2)-V(1)-O(4) | 84.79(9) |
| O(3)-V(1)-O(4) | 82.61(8) |
| N(2)-V(1)-O(4) | 77.36(8) |
| N(3)-V(1)-O(4) | 81.30(8) |
| N(1)-V(1)-O(4) | 86.18(8) |
| O(2)-O(3)-V(1) | 67.34(11) |
| O(3)-O(2)-V(1) | 68.25(12) |
| C(1M)-O(4)-V(1) | 127.12(19) |
| C(6)-N(2)-C(7) | 120.8(2) |
| C(6)-N(2)-V(1) | 121.35(18) |
| C(7)-N(2)-V(1) | 117.83(16) |
| C(1)-N(1)-C(5) | 118.4(2) |
| C(1)-N(1)-V(1) | 124.02(18) |
| C(5)-N(1)-V(1) | 117.57(18) |
| C(13)-N(3)-C(14) | 118.3(2) |
| C(13)-N(3)-V(1) | 125.5(2) |
| C(14)-N(3)-V(1) | 116.03(17) |
| N(1)-C(5)-C(4) | 122.4(3) |
| N(1)-C(5)-C(6) | 115.1(2) |
| C(4)-C(5)-C(6) | 122.4(2) |
| C(8)-C(7)-N(2) | 128.7(2) |
| C(8)-C(7)-C(14) | 118.5(2) |
| N(2)-C(7)-C(14) | 112.8(2) |
| N(3)-C(14)-C(15) | 122.1(2) |
| N(3)-C(14)-C(7) | 116.8(2) |
| C(15)-C(14)-C(7) | 121.1(2) |
| C(9)-C(10)-C(15) | 119.4(3) |
| C(11)-C(12)-C(13) | 118.8(3) |
| C(2)-C(3)-C(4) | 118.9(2) |
| O(5)-C(6)-N(2) | 127.9(2) |
| O(5)-C(6)-C(5) | 120.7(2) |
| N(2)-C(6)-C(5) | 111.3(2) |
| C(7)-C(8)-C(9) | 119.9(3) |
| C(10)-C(9)-C(8) | 122.4(3) |
| C(3)-C(2)-C(1) | 119.2(3) |
| C(11)-C(15)-C(10) | 124.2(3) |
| C(11)-C(15)-C(14) | 117.0(3) |
| C(10)-C(15)-C(14) | 118.7(3) |

| | |
|---|---|
| N(1)-C(1)-C(2) | 122.4(3) |
| C(5)-C(4)-C(3) | 118.6(2) |
| N(3)-C(13)-C(12) | 123.4(3) |
| C(12)-C(11)-C(15) | 120.3(3) |

Symmetry transformations used to generate equivalent atoms:

**Table S6.** Bond lengths [Å] and angles [°] for **2**

| | |
|---|---|
| V(1)-O(1) | 1.614(2) |
| V(1)-O(2) | 1.619(2) |
| V(1)-N(2) | 2.068(2) |
| V(1)-N(3) | 2.085(2) |
| V(1)-N(1) | 2.100(2) |
| O(5)-C(6) | 1.229(3) |
| N(2)-C(6) | 1.356(3) |
| N(2)-C(7) | 1.404(3) |
| N(1)-C(1) | 1.342(3) |
| N(1)-C(5) | 1.347(3) |
| N(3)-C(15) | 1.329(3) |
| N(3)-C(12) | 1.362(3) |
| C(7)-C(8) | 1.380(4) |
| C(7)-C(12) | 1.419(3) |
| C(12)-C(11) | 1.416(3) |
| C(11)-C(13) | 1.414(4) |
| C(11)-C(10) | 1.417(4) |
| C(5)-C(4) | 1.385(4) |
| C(5)-C(6) | 1.499(3) |
| C(9)-C(10) | 1.366(4) |
| C(9)-C(8) | 1.414(4) |
| C(9)-H(9) | 0.9500 |
| C(8)-H(8) | 0.9500 |
| C(3)-C(2) | 1.384(4) |
| C(3)-C(4) | 1.386(4) |
| C(3)-H(3) | 0.9500 |
| C(13)-C(14) | 1.369(4) |
| C(13)-H(13) | 0.9500 |
| C(14)-C(15) | 1.401(4) |
| C(14)-H(14) | 0.9500 |
| C(4)-H(4) | 0.9500 |
| C(15)-H(15) | 0.9500 |
| C(10)-H(10) | 0.9500 |
| C(1)-C(2) | 1.386(4) |
| C(1)-H(1) | 0.9500 |
| C(2)-H(2) | 0.9500 |
| | |
| O(1)-V(1)-O(2) | 111.11(11) |
| O(1)-V(1)-N(2) | 118.78(10) |
| O(2)-V(1)-N(2) | 130.06(10) |
| O(1)-V(1)-N(3) | 99.44(10) |
| O(2)-V(1)-N(3) | 98.17(10) |
| N(2)-V(1)-N(3) | 76.53(8) |
| O(1)-V(1)-N(1) | 98.14(10) |
| O(2)-V(1)-N(1) | 95.36(10) |
| N(2)-V(1)-N(1) | 76.17(8) |
| N(3)-V(1)-N(1) | 152.21(9) |
| C(6)-N(2)-C(7) | 122.5(2) |
| C(6)-N(2)-V(1) | 119.60(17) |
| C(7)-N(2)-V(1) | 117.36(16) |

| | |
|---|---|
| C(1)-N(1)-C(5) | 119.8(2) |
| C(1)-N(1)-V(1) | 123.30(18) |
| C(5)-N(1)-V(1) | 116.84(16) |
| C(15)-N(3)-C(12) | 119.7(2) |
| C(15)-N(3)-V(1) | 123.85(18) |
| C(12)-N(3)-V(1) | 116.40(17) |
| C(8)-C(7)-N(2) | 129.1(2) |
| C(8)-C(7)-C(12) | 118.2(2) |
| N(2)-C(7)-C(12) | 112.7(2) |
| N(3)-C(12)-C(11) | 121.8(2) |
| N(3)-C(12)-C(7) | 116.6(2) |
| C(11)-C(12)-C(7) | 121.6(2) |
| C(13)-C(11)-C(12) | 117.1(2) |
| C(13)-C(11)-C(10) | 124.5(2) |
| C(12)-C(11)-C(10) | 118.4(2) |
| N(1)-C(5)-C(4) | 121.8(2) |
| N(1)-C(5)-C(6) | 115.3(2) |
| C(4)-C(5)-C(6) | 122.8(2) |
| O(5)-C(6)-N(2) | 128.9(2) |
| O(5)-C(6)-C(5) | 119.7(2) |
| N(2)-C(6)-C(5) | 111.3(2) |
| C(10)-C(9)-C(8) | 122.1(2) |
| C(10)-C(9)-H(9) | 119.0 |
| C(8)-C(9)-H(9) | 119.0 |
| C(7)-C(8)-C(9) | 120.3(2) |
| C(7)-C(8)-H(8) | 119.9 |
| C(9)-C(8)-H(8) | 119.9 |
| C(2)-C(3)-C(4) | 119.2(2) |
| C(2)-C(3)-H(3) | 120.4 |
| C(4)-C(3)-H(3) | 120.4 |
| C(14)-C(13)-C(11) | 120.0(2) |
| C(14)-C(13)-H(13) | 120.0 |
| C(11)-C(13)-H(13) | 120.0 |
| C(13)-C(14)-C(15) | 119.6(2) |
| C(13)-C(14)-H(14) | 120.2 |
| C(15)-C(14)-H(14) | 120.2 |
| C(5)-C(4)-C(3) | 118.6(2) |
| C(5)-C(4)-H(4) | 120.7 |
| C(3)-C(4)-H(4) | 120.7 |
| N(3)-C(15)-C(14) | 121.8(3) |
| N(3)-C(15)-H(15) | 119.1 |
| C(14)-C(15)-H(15) | 119.1 |
| C(9)-C(10)-C(11) | 119.4(2) |
| C(9)-C(10)-H(10) | 120.3 |
| C(11)-C(10)-H(10) | 120.3 |
| N(1)-C(1)-C(2) | 120.9(2) |
| N(1)-C(1)-H(1) | 119.5 |
| C(2)-C(1)-H(1) | 119.5 |
| C(3)-C(2)-C(1) | 119.6(2) |
| C(3)-C(2)-H(2) | 120.2 |
| C(1)-C(2)-H(2) | 120.2 |

Symmetry transformations used to generate equivalent atoms:

**Table S7.** Bond lengths [Å] and angles [°] for **4**

| | |
|---|---|
| V(1)-O(2) | 1.618(5) |
| V(1)-O(1) | 1.619(4) |
| V(1)-N(2) | 2.024(4) |

| | |
|---|---|
| V(1)-N(1) | 2.099(5) |
| V(1)-N(3) | 2.110(5) |
| O(17)-C(14) | 1.230(7) |
| N(2)-C(14) | 1.331(7) |
| N(2)-C(16) | 1.455(7) |
| N(3)-C(22) | 1.349(7) |
| N(3)-C(18) | 1.355(6) |
| N(1)-C(4) | 1.324(7) |
| N(1)-C(12) | 1.369(7) |
| C(19)-C(20) | 1.382(8) |
| C(19)-C(18) | 1.389(7) |
| C(19)-H(19) | 0.9300 |
| C(4)-C(5) | 1.418(7) |
| C(4)-C(14) | 1.522(7) |
| C(18)-C(16) | 1.490(7) |
| C(10)-C(9) | 1.406(8) |
| C(10)-C(11) | 1.417(8) |
| C(10)-C(5) | 1.429(7) |
| C(5)-C(6) | 1.418(7) |
| C(20)-C(21) | 1.377(8) |
| C(6)-C(7) | 1.369(8) |
| C(22)-C(21) | 1.379(9) |
| C(8)-C(9) | 1.382(9) |
| C(8)-C(7) | 1.408(9) |
| C(12)-C(11) | 1.357(8) |

| | |
|---|---|
| O(2)-V(1)-O(1) | 111.3(2) |
| O(2)-V(1)-N(2) | 122.5(2) |
| O(1)-V(1)-N(2) | 126.2(2) |
| O(2)-V(1)-N(1) | 98.7(2) |
| O(1)-V(1)-N(1) | 96.8(2) |
| N(2)-V(1)-N(1) | 74.99(18) |
| O(2)-V(1)-N(3) | 98.2(2) |
| O(1)-V(1)-N(3) | 98.7(2) |
| N(2)-V(1)-N(3) | 76.11(17) |
| N(1)-V(1)-N(3) | 151.05(18) |
| C(14)-N(2)-C(16) | 116.7(4) |
| C(14)-N(2)-V(1) | 122.3(4) |
| C(16)-N(2)-V(1) | 121.0(3) |
| C(22)-N(3)-C(18) | 119.1(5) |
| C(22)-N(3)-V(1) | 122.5(4) |
| C(18)-N(3)-V(1) | 118.4(4) |
| C(4)-N(1)-C(12) | 120.2(5) |
| C(4)-N(1)-V(1) | 118.9(4) |
| C(12)-N(1)-V(1) | 120.9(4) |
| C(20)-C(19)-C(18) | 119.4(5) |
| N(1)-C(4)-C(5) | 122.6(5) |
| N(1)-C(4)-C(14) | 112.6(5) |
| C(5)-C(4)-C(14) | 124.7(4) |
| O(17)-C(14)-N(2) | 125.8(5) |
| O(17)-C(14)-C(4) | 123.1(5) |
| N(2)-C(14)-C(4) | 111.1(4) |
| N(3)-C(18)-C(19) | 121.3(5) |
| N(3)-C(18)-C(16) | 115.3(5) |
| C(19)-C(18)-C(16) | 123.4(5) |
| C(9)-C(10)-C(11) | 122.0(5) |
| C(9)-C(10)-C(5) | 119.9(5) |
| C(11)-C(10)-C(5) | 118.1(5) |
| C(6)-C(5)-C(4) | 124.6(5) |
| C(6)-C(5)-C(10) | 118.3(5) |

| | |
|---|---|
| C(4)-C(5)-C(10) | 117.2(5) |
| C(21)-C(20)-C(19) | 118.8(5) |
| C(7)-C(6)-C(5) | 120.2(5) |
| N(3)-C(22)-C(21) | 121.4(5) |
| C(9)-C(8)-C(7) | 118.9(6) |
| C(20)-C(21)-C(22) | 120.1(6) |
| C(6)-C(7)-C(8) | 122.0(5) |
| C(11)-C(12)-N(1) | 121.5(5) |
| C(8)-C(9)-C(10) | 120.9(5) |
| N(2)-C(16)-C(18) | 109.0(4) |
| C(12)-C(11)-C(10) | 120.4(5) |

Symmetry transformations used to generate equivalent atoms:

**Table S8.** Bond lengths [Å] and angles [°] for **5.**

| | |
|---|---|
| V(1)-O(1) | 1.6211(15) |
| V(1)-O(2) | 1.6277(16) |
| V(1)-N(2) | 2.021(2) |
| V(1)-N(3) | 2.1004(18) |
| V(1)-N(1) | 2.1217(18) |
| O(5)-C(7) | 1.230(3) |
| N(3)-C(5) | 1.341(3) |
| N(3)-C(1) | 1.342(3) |
| N(2)-C(7) | 1.340(3) |
| N(2)-C(6) | 1.454(3) |
| N(1)-C(8) | 1.328(3) |
| N(1)-C(12) | 1.377(3) |
| C(2)-C(1) | 1.379(3) |
| C(2)-C(3) | 1.388(3) |
| C(2)-H(2) | 0.92(3) |
| C(9)-C(10) | 1.362(3) |
| C(9)-C(8) | 1.398(3) |
| C(9)-H(7) | 0.94(3) |
| C(12)-C(13) | 1.409(3) |
| C(12)-C(11) | 1.428(3) |
| C(11)-C(10) | 1.412(3) |
| C(11)-C(016) | 1.415(3) |
| C(5)-C(4) | 1.389(3) |
| C(5)-C(6) | 1.497(3) |
| C(13)-C(14) | 1.365(3) |
| C(13)-H(12) | 0.91(3) |
| C(10)-H(8) | 0.94(3) |
| C(3)-C(4) | 1.385(3) |
| C(3)-H(3) | 0.93(3) |
| C(016)-C(15) | 1.357(3) |
| C(016)-H(9) | 0.93(3) |
| C(6)-H(6) | 0.99(2) |
| C(6)-H(5) | 0.97(3) |
| C(4)-H(4) | 0.93(3) |
| C(14)-C(15) | 1.406(3) |
| C(14)-H(11) | 0.96(3) |
| C(15)-H(10) | 0.95(3) |
| C(8)-C(7) | 1.500(3) |
| C(1)-H(1) | 0.95(3) |
| | |
| O(1)-V(1)-O(2) | 110.67(9) |
| O(1)-V(1)-N(2) | 121.73(8) |
| O(2)-V(1)-N(2) | 127.60(8) |

| | |
|---|---|
| O(1)-V(1)-N(3) | 97.55(7) |
| O(2)-V(1)-N(3) | 98.50(8) |
| N(2)-V(1)-N(3) | 75.55(7) |
| O(1)-V(1)-N(1) | 99.12(7) |
| O(2)-V(1)-N(1) | 96.49(7) |
| N(2)-V(1)-N(1) | 76.52(7) |
| N(3)-V(1)-N(1) | 151.96(8) |
| C(5)-N(3)-C(1) | 119.95(19) |
| C(5)-N(3)-V(1) | 118.67(14) |
| C(1)-N(3)-V(1) | 121.19(15) |
| C(7)-N(2)-C(6) | 117.15(18) |
| C(7)-N(2)-V(1) | 120.67(14) |
| C(6)-N(2)-V(1) | 122.00(14) |
| C(8)-N(1)-C(12) | 118.98(19) |
| C(8)-N(1)-V(1) | 115.25(14) |
| C(12)-N(1)-V(1) | 125.76(15) |
| C(1)-C(2)-C(3) | 119.0(2) |
| C(1)-C(2)-H(2) | 117.5(16) |
| C(3)-C(2)-H(2) | 123.5(16) |
| C(10)-C(9)-C(8) | 119.3(2) |
| C(10)-C(9)-H(7) | 123.4(15) |
| C(8)-C(9)-H(7) | 117.3(15) |
| N(1)-C(12)-C(13) | 120.5(2) |
| N(1)-C(12)-C(11) | 120.18(19) |
| C(13)-C(12)-C(11) | 119.3(2) |
| C(10)-C(11)-C(016) | 122.5(2) |
| C(10)-C(11)-C(12) | 118.7(2) |
| C(016)-C(11)-C(12) | 118.8(2) |
| N(3)-C(5)-C(4) | 121.1(2) |
| N(3)-C(5)-C(6) | 115.93(19) |
| C(4)-C(5)-C(6) | 123.0(2) |
| C(14)-C(13)-C(12) | 120.0(2) |
| C(14)-C(13)-H(12) | 121.3(17) |
| C(12)-C(13)-H(12) | 118.6(17) |
| C(9)-C(10)-C(11) | 119.4(2) |
| C(9)-C(10)-H(8) | 121.2(17) |
| C(11)-C(10)-H(8) | 119.4(17) |
| C(4)-C(3)-C(2) | 119.0(2) |
| C(4)-C(3)-H(3) | 119.2(17) |
| C(2)-C(3)-H(3) | 121.7(17) |
| C(15)-C(016)-C(11) | 120.3(2) |
| C(15)-C(016)-H(9) | 121.9(18) |
| C(11)-C(016)-H(9) | 117.8(18) |
| N(2)-C(6)-C(5) | 107.35(18) |
| N(2)-C(6)-H(6) | 111.8(14) |
| C(5)-C(6)-H(6) | 110.4(14) |
| N(2)-C(6)-H(5) | 112.1(15) |
| C(5)-C(6)-H(5) | 113.0(15) |
| H(6)-C(6)-H(5) | 102(2) |
| C(3)-C(4)-C(5) | 119.2(2) |
| C(3)-C(4)-H(4) | 123.1(16) |
| C(5)-C(4)-H(4) | 117.6(16) |
| C(13)-C(14)-C(15) | 120.9(2) |
| C(13)-C(14)-H(11) | 120.6(16) |
| C(15)-C(14)-H(11) | 118.5(16) |
| C(016)-C(15)-C(14) | 120.7(2) |
| C(016)-C(15)-H(10) | 122.0(16) |
| C(14)-C(15)-H(10) | 117.1(16) |
| N(1)-C(8)-C(9) | 123.5(2) |
| N(1)-C(8)-C(7) | 115.73(19) |

| | |
|---|---|
| C(9)-C(8)-C(7) | 120.78(19) |
| O(5)-C(7)-N(2) | 127.8(2) |
| O(5)-C(7)-C(8) | 120.86(19) |
| N(2)-C(7)-C(8) | 111.36(19) |
| N(3)-C(1)-C(2) | 121.7(2) |
| N(3)-C(1)-H(1) | 115.3(15) |
| C(2)-C(1)-H(1) | 122.9(15) |

Symmetry transformations used to generate equivalent atoms:

**Table S9.** Bond lengths [Å] and angles [°] for **6**

| | |
|---|---|
| V(1')-O(2') | 1.619(2) |
| V(1')-O(1') | 1.621(2) |
| V(1')-N(2') | 2.064(2) |
| V(1')-N(3') | 2.098(2) |
| V(1')-N(1') | 2.099(2) |
| V(1)-O(1) | 1.620(2) |
| V(1)-O(2) | 1.621(2) |
| V(1)-N(2) | 2.085(2) |
| V(1)-N(1) | 2.094(3) |
| V(1)-N(3) | 2.097(3) |
| N(2')-C(13) | 1.297(4) |
| N(2')-C(18) | 1.432(4) |
| O(5)-C(37) | 1.230(3) |
| O(5')-C(13) | 1.234(4) |
| N(2)-C(37) | 1.286(4) |
| N(2)-C(28) | 1.414(4) |
| N(3')-C(25) | 1.322(4) |
| N(3')-C(17) | 1.366(4) |
| N(1)-C(40) | 1.321(4) |
| N(1)-C(48) | 1.379(4) |
| N(1')-C(4) | 1.319(3) |
| N(1')-C(12) | 1.374(4) |
| N(3)-C(35) | 1.323(4) |
| N(3)-C(27) | 1.348(4) |
| C(41)-C(42) | 1.373(4) |
| C(41)-C(40) | 1.403(4) |
| C(37)-C(40) | 1.530(4) |
| C(22)-C(17) | 1.405(4) |
| C(22)-C(23) | 1.412(4) |
| C(22)-C(21) | 1.415(4) |
| C(10)-C(11) | 1.369(4) |
| C(10)-C(9) | 1.414(5) |
| C(13)-C(4) | 1.510(4) |
| C(4)-C(5) | 1.407(4) |
| C(25)-C(24) | 1.401(4) |
| C(17)-C(18) | 1.418(4) |
| C(19)-C(18) | 1.377(4) |
| C(19)-C(20) | 1.412(4) |
| C(5)-C(6) | 1.354(4) |
| C(43)-C(44) | 1.406(4) |
| C(43)-C(48) | 1.414(4) |
| C(43)-C(42) | 1.417(4) |
| C(12)-C(7) | 1.417(4) |
| C(12)-C(11) | 1.428(4) |
| C(29)-C(28) | 1.361(4) |
| C(29)-C(30) | 1.419(4) |
| C(7)-C(8) | 1.402(4) |

| | |
|---|---|
| C(7)-C(6) | 1.421(4) |
| C(47)-C(46) | 1.358(4) |
| C(47)-C(48) | 1.423(4) |
| C(28)-C(27) | 1.431(4) |
| C(27)-C(32) | 1.409(4) |
| C(32)-C(31) | 1.408(4) |
| C(32)-C(33) | 1.423(5) |
| C(34)-C(33) | 1.373(5) |
| C(34)-C(35) | 1.394(4) |
| C(24)-C(23) | 1.368(5) |
| C(20)-C(21) | 1.371(5) |
| C(30)-C(31) | 1.362(5) |
| C(8)-C(9) | 1.356(5) |
| C(46)-C(45) | 1.407(5) |
| C(44)-C(45) | 1.350(5) |

| | |
|---|---|
| O(2')-V(1')-O(1') | 111.20(12) |
| O(2')-V(1')-N(2') | 124.05(11) |
| O(1')-V(1')-N(2') | 124.74(11) |
| O(2')-V(1')-N(3') | 97.24(10) |
| O(1')-V(1')-N(3') | 97.63(10) |
| N(2')-V(1')-N(3') | 76.83(10) |
| O(2')-V(1')-N(1') | 98.11(10) |
| O(1')-V(1')-N(1') | 98.07(10) |
| N(2')-V(1')-N(1') | 75.51(10) |
| N(3')-V(1')-N(1') | 152.34(9) |
| O(1)-V(1)-O(2) | 111.93(12) |
| O(1)-V(1)-N(2) | 121.48(10) |
| O(2)-V(1)-N(2) | 126.59(10) |
| O(1)-V(1)-N(1) | 97.80(10) |
| O(2)-V(1)-N(1) | 98.66(10) |
| N(2)-V(1)-N(1) | 75.86(10) |
| O(1)-V(1)-N(3) | 97.43(10) |
| O(2)-V(1)-N(3) | 96.33(10) |
| N(2)-V(1)-N(3) | 77.01(10) |
| N(1)-V(1)-N(3) | 152.83(10) |
| C(13)-N(2')-C(18) | 122.0(2) |
| C(13)-N(2')-V(1') | 120.7(2) |
| C(18)-N(2')-V(1') | 116.95(19) |
| C(37)-N(2)-C(28) | 123.9(2) |
| C(37)-N(2)-V(1) | 120.11(19) |
| C(28)-N(2)-V(1) | 115.88(19) |
| C(25)-N(3')-C(17) | 119.1(3) |
| C(25)-N(3')-V(1') | 124.0(2) |
| C(17)-N(3')-V(1') | 116.86(18) |
| C(40)-N(1)-C(48) | 118.2(3) |
| C(40)-N(1)-V(1) | 116.9(2) |
| C(48)-N(1)-V(1) | 124.8(2) |
| C(4)-N(1')-C(12) | 118.2(2) |
| C(4)-N(1')-V(1') | 116.51(19) |
| C(12)-N(1')-V(1') | 125.29(19) |
| C(35)-N(3)-C(27) | 118.5(3) |
| C(35)-N(3)-V(1) | 124.6(2) |
| C(27)-N(3)-V(1) | 116.8(2) |
| C(42)-C(41)-C(40) | 117.3(3) |
| O(5)-C(37)-N(2) | 129.4(3) |
| O(5)-C(37)-C(40) | 118.4(3) |
| N(2)-C(37)-C(40) | 112.2(2) |
| N(1)-C(40)-C(41) | 125.2(3) |
| N(1)-C(40)-C(37) | 114.7(3) |

| | |
|---|---|
| C(41)-C(40)-C(37) | 120.1(3) |
| C(17)-C(22)-C(23) | 117.2(3) |
| C(17)-C(22)-C(21) | 118.7(3) |
| C(23)-C(22)-C(21) | 124.1(3) |
| C(11)-C(10)-C(9) | 120.9(3) |
| O(5')-C(13)-N(2') | 129.7(3) |
| O(5')-C(13)-C(4) | 119.0(3) |
| N(2')-C(13)-C(4) | 111.3(3) |
| N(1')-C(4)-C(5) | 124.6(3) |
| N(1')-C(4)-C(13) | 115.1(3) |
| C(5)-C(4)-C(13) | 120.3(3) |
| N(3')-C(25)-C(24) | 122.3(3) |
| N(3')-C(17)-C(22) | 122.1(3) |
| N(3')-C(17)-C(18) | 116.1(3) |
| C(22)-C(17)-C(18) | 121.8(3) |
| C(18)-C(19)-C(20) | 120.7(3) |
| C(6)-C(5)-C(4) | 118.2(3) |
| C(44)-C(43)-C(48) | 119.3(3) |
| C(44)-C(43)-C(42) | 122.0(3) |
| C(48)-C(43)-C(42) | 118.8(3) |
| N(1')-C(12)-C(7) | 120.9(3) |
| N(1')-C(12)-C(11) | 120.2(3) |
| C(7)-C(12)-C(11) | 118.9(3) |
| C(28)-C(29)-C(30) | 120.4(3) |
| C(8)-C(7)-C(12) | 119.8(3) |
| C(8)-C(7)-C(6) | 121.9(3) |
| C(12)-C(7)-C(6) | 118.3(3) |
| C(46)-C(47)-C(48) | 119.0(3) |
| C(29)-C(28)-N(2) | 127.8(3) |
| C(29)-C(28)-C(27) | 118.2(3) |
| N(2)-C(28)-C(27) | 114.0(3) |
| N(3)-C(27)-C(32) | 122.7(3) |
| N(3)-C(27)-C(28) | 116.3(3) |
| C(32)-C(27)-C(28) | 121.0(3) |
| C(5)-C(6)-C(7) | 119.8(3) |
| C(31)-C(32)-C(27) | 119.1(3) |
| C(31)-C(32)-C(33) | 123.9(3) |
| C(27)-C(32)-C(33) | 117.0(3) |
| C(33)-C(34)-C(35) | 118.4(3) |
| N(1)-C(48)-C(43) | 120.6(3) |
| N(1)-C(48)-C(47) | 120.3(3) |
| C(43)-C(48)-C(47) | 119.1(3) |
| C(23)-C(24)-C(25) | 119.3(3) |
| C(10)-C(11)-C(12) | 119.4(3) |
| C(19)-C(18)-C(17) | 118.0(3) |
| C(19)-C(18)-N(2') | 128.8(3) |
| C(17)-C(18)-N(2') | 113.2(2) |
| C(41)-C(42)-C(43) | 119.9(3) |
| N(3)-C(35)-C(34) | 123.7(3) |
| C(24)-C(23)-C(22) | 119.9(3) |
| C(21)-C(20)-C(19) | 121.5(3) |
| C(31)-C(30)-C(29) | 122.1(3) |
| C(9)-C(8)-C(7) | 120.5(3) |
| C(47)-C(46)-C(45) | 122.0(3) |
| C(8)-C(9)-C(10) | 120.5(3) |
| C(20)-C(21)-C(22) | 119.4(3) |
| C(45)-C(44)-C(43) | 121.0(3) |
| C(34)-C(33)-C(32) | 119.6(3) |
| C(30)-C(31)-C(32) | 119.0(3) |
| C(44)-C(45)-C(46) | 119.6(3) |

Symmetry transformations used to generate equivalent atoms:

**Table S10.** Bond lengths [Å] and angles [°] for **9**

| | |
|---|---|
| V(1)-O(1) | 1.621(2) |
| V(1)-O(2) | 1.638(2) |
| V(1)-N(1) | 2.120(2) |
| V(1)-N(3) | 2.124(2) |
| V(1)-N(2) | 2.144(2) |
| V(1)-O(3) | 2.305(2) |
| O(3)-C(4) | 1.429(4) |
| O(3)-H(3) | 0.863(9) |
| N(1)-C(23) | 1.345(3) |
| N(1)-C(19) | 1.360(3) |
| N(3)-C(16) | 1.345(4) |
| N(3)-C(12) | 1.367(4) |
| N(2)-C(11) | 1.336(3) |
| N(2)-C(7) | 1.346(3) |
| C(7)-C(8) | 1.390(4) |
| C(7)-C(12) | 1.470(4) |
| C(12)-C(13) | 1.391(4) |
| C(11)-C(10) | 1.390(4) |
| C(11)-C(19) | 1.487(4) |
| C(19)-C(20) | 1.385(4) |
| C(10)-C(9) | 1.386(4) |
| C(8)-C(9) | 1.386(4) |
| C(16)-C(15) | 1.385(4) |
| C(15)-C(14) | 1.383(4) |
| C(20)-C(21) | 1.395(4) |
| C(23)-C(22) | 1.382(4) |
| C(13)-C(14) | 1.383(4) |
| C(21)-C(22) | 1.380(4) |
| | |
| O(1)-V(1)-O(2) | 106.19(11) |
| O(1)-V(1)-N(1) | 94.62(9) |
| O(2)-V(1)-N(1) | 104.80(11) |
| O(1)-V(1)-N(3) | 94.77(10) |
| O(2)-V(1)-N(3) | 101.90(11) |
| N(1)-V(1)-N(3) | 147.89(9) |
| O(1)-V(1)-N(2) | 95.76(9) |
| O(2)-V(1)-N(2) | 157.99(10) |
| N(1)-V(1)-N(2) | 74.40(9) |
| N(3)-V(1)-N(2) | 74.15(9) |
| O(1)-V(1)-O(3) | 169.49(9) |
| O(2)-V(1)-O(3) | 84.30(9) |
| N(1)-V(1)-O(3) | 82.97(8) |
| N(3)-V(1)-O(3) | 82.26(8) |
| N(2)-V(1)-O(3) | 73.74(8) |
| C(4)-O(3)-V(1) | 122.47(18) |
| C(4)-O(3)-H(3) | 105.2(14) |
| V(1)-O(3)-H(3) | 124.3(14) |
| C(23)-N(1)-C(19) | 118.9(2) |
| C(23)-N(1)-V(1) | 122.44(19) |
| C(19)-N(1)-V(1) | 118.28(17) |
| C(16)-N(3)-C(12) | 118.8(2) |
| C(16)-N(3)-V(1) | 122.84(19) |
| C(12)-N(3)-V(1) | 118.33(19) |
| C(11)-N(2)-C(7) | 121.5(2) |

| | |
|---|---|
| C(11)-N(2)-V(1) | 119.06(18) |
| C(7)-N(2)-V(1) | 119.43(18) |
| N(2)-C(7)-C(8) | 120.6(3) |
| N(2)-C(7)-C(12) | 113.1(2) |
| C(8)-C(7)-C(12) | 126.3(2) |
| N(3)-C(12)-C(13) | 121.2(3) |
| N(3)-C(12)-C(7) | 114.9(2) |
| C(13)-C(12)-C(7) | 123.9(3) |
| N(2)-C(11)-C(10) | 121.0(2) |
| N(2)-C(11)-C(19) | 113.4(2) |
| C(10)-C(11)-C(19) | 125.6(3) |
| N(1)-C(19)-C(20) | 122.1(2) |
| N(1)-C(19)-C(11) | 114.3(2) |
| C(20)-C(19)-C(11) | 123.6(2) |
| C(9)-C(10)-C(11) | 117.8(3) |
| C(9)-C(8)-C(7) | 117.9(3) |
| N(3)-C(16)-C(15) | 122.5(3) |
| C(14)-C(15)-C(16) | 118.5(3) |
| C(19)-C(20)-C(21) | 118.4(3) |
| N(1)-C(23)-C(22) | 121.7(3) |
| C(14)-C(13)-C(12) | 118.9(3) |
| C(22)-C(21)-C(20) | 119.2(3) |
| C(10)-C(9)-C(8) | 121.2(3) |
| C(15)-C(14)-C(13) | 120.0(3) |
| C(21)-C(22)-C(23) | 119.7(3) |

Symmetry transformations used to generate equivalent atoms:

**Table S11.** Resonance Raman peaks (excitation at 368.91 nm) experimental, calculated by theory (values in parenthesis) and assignments.

| **Hpbq**($cm^{-1}$) | $[V^VO(\eta^2-O_2)(pbq)(H_2O)]^{\bullet+}$($cm^{-1}$) | **1** ($cm^{-1}$) | **Assignment†** |
|---|---|---|---|
| 998 (1024) | N/A | N/A | Ringbreathingpyridine |
| N/A | 1031 (1062) | N/A | $\nu_{O-O}$ |
| 1129 (1182) | N/A | N/A | $\delta_{C-H}$ pyridine/quinoline |
| 1143 (1216) | 1159 (1229) | 1155 (1192) | $\delta_{C-H}$ quinoline |
| 1201 (1258) | 1200 (1268) | * | $\Delta$ + $\delta_{C-H}$ quinoline (+ $\delta_{N-H}$ amide in ligand) |
| 1260 (1301) | | | $\delta_{N-H}$ amide III + $\delta_{C-H}$ quinoline, $\nu_{C-C(O)}$ |
| 1281 (1329) | 1274 (1337) | 1272 (1321) | $\delta_{C-H}$ pyridine |
| 1297 (1366) | | | $\nu_{C=C}$ + $\delta_{C-H}$ pyridine + $\delta_{N-H}$ |
| 1326 (1393) | 1315 (1386) | 1312 (1387) | $\Delta$ + $\delta_{C-H}$ quinoline (+ $\delta_{N-H}$amide in ligand) |
| 1387 (1449) | 1392 (1461) | 1392 (1446) | $\nu_{C=C}$ + $\delta_{C-H}$ quinoline |
| 1426 (1476) | 1425 (1489) | N/A | $\Delta$ + $\delta_{C-H}$ quinoline |
| 1434 (1483) | | | $\Delta$ + $\delta_{C-H}$ pyridine |
| 1461 (1515) | 1450 (1521) | 1448 (1496) | $\delta_{N-H}$ (in ligand) +$\delta_{C-H}$ pyridine/quinolone (+$\nu_{C-N}$ in complex) |

| | | | |
|---|---|---|---|
| 1488 (1557) | 1475 (1558) | 1480 (1536) | $\Delta$+ $\delta_{C\text{-}H}$ quinolone (+ $\delta_{N\text{-}H}$amide) |
| | 1505 (1588) | 1505 (1575) | $\nu_{C=C}$ + $\delta_{C\text{-}H}$ quinoline |
| 1531 (1604) | N/A | N/A | $\nu_{C=N}$ +$\delta_{N\text{-}H}$ amideII |
| 1580 (1649) | 1581 (1661) | 1581 (1647) | $\nu_{C=C}$ + $\delta_{C\text{-}H}$ quinoline |
| 1591 (1665/1668) | | | $\nu_{C=C}$ + $\delta_{C\text{-}H}$ pyridine/quinoline |
| | 1602 (1679) | 1608 (1684) | $\nu_{C=C}$ + $\delta_{C\text{-}H}$ pyridine |

*not a clear region

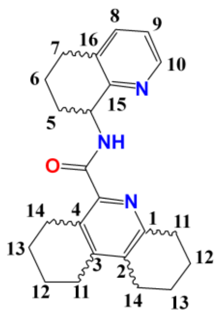


**Table S12.** $^{1}$H NMR chemical shifts and assigmants of ligands and complexes **1-6**.

| **Solvent** | | | | | | | | | | | | | |
|---|---|---|---|---|---|---|---|---|---|---|---|---|---|
| | | **H(10)** | **H(1)** | **H(8)** | **H(4)** | **H(5)** | **H(3)** | **H(6)** | **H(2)** | **H(9)** | **H(7)** | | |
| CD3OD | **L1** | 8.980 | 8.810 | 8.810 | 8.404 | 8.278 | 8.106 | 7.749 | 7.701 | 7.766 | 7.766 | | |
| CD3OD | **1** | 9.988 | 9.982 | 9.254 | 8.796 | 8.385 | 8.486 | 8.069 | 8.011 | 7.856 | 7.820 | | |
| CD3OD | **2** | 9.033 | 8.823 | 8.716 | 8.615 | 8.294 | 8.133 | 7.901 | 7.861 | 7.755 | 7.728 | | |
| | | **H(14)** | **H(1)** | **H(10)** | **H(2)** | **H(13)** | **H(12)** | **H(8)** | **H(11)** | **H(16)** | **H(9)** | **H(15)** | |
| CD3OD | **L4** | 9.076 | 8.576 | 8.553 | 8.019 | 7.988 | 7.877 | 7.824 | 7.736 | 7.583 | 7.357 | 4.822 | |
| CD3OD | **3** | 9.579 | 9.534 | 10.07 | 8.382 | 8.241 | 8.026 | 7.931 | 8.613 | 8.198 | 7.791 | 4.882 | |
| CD3OD | **4** | 8.712 | 9.548 | 9.589 | 8.297 | 8.088 | 7.953 | 7.617 | 8.216 | 8.065 | 7.550 | 4.680 | |
| | | **H(3)** | **H(10)** | **H(14)** | **H(11)** | **H(16)** | **H(12)** | **H(8)** | **H(13)** | **H(4)** | **H(9)** | **H(15)** | |
| CD3OD | **L5** | 8.549 | 8.509 | 8.235 | 8.217 | 8.030 | 7.860 | 7.844 | 7.718 | 7.502 | 7.357 | 4.827 | |
| CD3OD | **5** | 8.946 | 9.410 | 8.293 | 9.326 | 8.273 | 8.123 | 7.931 | 7.905 | 7.857 | 7.834 | 4.683 | |
| | | **H(3)** | **H(10)** | **H(8)** | **H(14)** | **H(11)** | **H(5)** | **H(7)** | **H(12)** | **H(13)** | **H(9)** | **H(6)** | **H(4)** |
| CD3OD | **L6** | 9.058 | 8.871 | 8.573 | 8.477 | 8.393 | 8.369 | 8.341 | 8.062 | 7.923 | 7.753 | 7.656 | 7.641 |
| CD3Cl | **6** | 8.995 | 9.864 | 9.059 | 8.837 | 9.443 | 8.454 | 8.152 | 8.262 | 8.077 | 8.102 | 7.877 | 7.955 |
| | | **H(1)** | **H(10)** | **H(16)** | **H(8)** | **H(9)** | **H(3)** | **H(4)** | **H(2)** | **H(15)** | | | |
| CD3OD | **L2** | 8.671 | 8.534 | 8.134 | 7.984 | 7.865 | 7.560 | 7.476 | 7.360 | 4.770 | | | |
| | | **H(14)** | **H(10)** | **H(1)** | **H(8)** | **H(2)** | **H(5)** | **H(11)** | **H(13)** | **H(12)** | **H(9)** | **H(7)** | **H(6)** |
| CD3OD | **L3** | 9.695 | 9.092 | 9.003 | 8.748 | 8.274 | 7.926 | 7.924 | 7.794 | 7.774 | 7.689 | 7.648 | 7.547 |

**Table S13**. [51]V NMR chemical shifts (ppm) of the 25:75 $D_2O$:$CD_3OD$ solution of peroxido and dioxide vanadium complexes.

| | Peroxide Vanadate (**2**) | Dioxido Vanadate (**3**) |
|---|---|---|
| **L1** | -645 | -507 |
| **L2** | -617 | -497 |
| **L3** | -634 | -503 |
| **L4** | -606 | -501 |
| **L5** | | -489 |
| **L6** | | -496 |
| **terpy** | -619 | -505 |

**Table S14.** Redox potentials (mV) and assignments of the waves from the CVs of the $CH_3CN$ solutions of pdb⁻ and the complexes $[V^{IV}O(pbq)(H_2O)]^+$, $[V^VO_2(pbq)]$, $[V^VO(\eta^2\text{-}O_2)(pbq)(H_2O)]$ and $[V^VO(\eta^2\text{-}O_2)(pbq)(H_2O)]$.

| **reaction** | **pbq⁻** | $[V^{IV}O(pbq)(H_2O)]^+$ | $[V^VO_2(pbq)]$ | $[V^VO(\eta^2\text{-}O_2)(pbq)(H_2O)]$ | $[V^VO(\eta^2\text{-}O_2)(pbq)(H_2O)]^{\bullet+}$ |
|---|---|---|---|---|---|
| $pbq^- + e^- \rightarrow pbq^{\bullet 2-}$ | -891 | | -924 | -837 | |
| $pbq^- \rightarrow pbq^\bullet + e^-$ | 1775 | 1758 | 1780 | 1740 | 1800 |
| $[V^VO(\eta^2\text{-}O_2)(pbq)(H_2O)] \rightarrow [V^VO(\eta^2\text{-}O_2)(pbq)(H_2O)]^{2+} + 2e^-$ | | | | 1633 | |
| $[V^VO(\eta^2\text{-}O_2)(pbq)(H_2O)]^{\bullet+} \rightarrow [V^VO(\eta^2\text{-}O_2)(pbq)(H_2O)]^{2+} + e^-$ | | | | | 1202 |
| $[V^VO(\eta^2\text{-}O_2)(pbq)(H_2O)]^{2+} + e^- \rightarrow [V^VO(\eta^2\text{-}O_2)(pbq)(H_2O)]^{\bullet+}$ | | | | 1035 | ~1040 |
| $[V^VO(\eta^2\text{-}O_2)(pbq)(H_2O)]^{\bullet+} + e^- \rightarrow [V^VO(\eta^2\text{-}O_2)(pbq)(H_2O)]$ | | | | | -35 |
| $[V^{III}O(pbq)(H_2O)] \rightarrow [V^{IV}O(pbq)(H_2O)]^+ + e^-$ | | -28 | | | |
| $[V^{IV}O(pbq)(H_2O)]^+ + e^- \rightarrow [V^{III}O(pbq)(H_2O)]$ | | -314 | | | |

**References**

1. *CrysAlis CCD*, 1.171.29.9; Oxford Diffraction Ltd: 2006.
2. *CrysAlis RED*, 1.171.29.9; Oxford Diffraction Ltd: 2006.
3. Sheldrick, G. M., *SHELXL-97: Program for the Refinement of Crystal Structure*. University of Göttingen: Göttingen, Germany, **1997**.
4. Sheldrick, G. M., *SHELXS-97: Program for the Solution of Crystal Structure*. University of Göttingen: Göttingen, Germany, **1997**.
5. Farrugia, L. J. WinGX suite for small-molecule single-crystal crystallography. *Journal of Applied Crystallography* **1999,** 32 (4), 837-838.
6. Stoll, S.; Schweiger, A. EasySpin, a comprehensive software package for spectral simulation and analysis in EPR. *J. Magn. Reson.* **2006,** 178, 42-55.
7. Pieridou, G. K.; Hayes, S. C. UV resonance Raman spectroscopy of TTR(105-115): Determination of the pKa of tyrosine. *Physical Chemistry Chemical Physics* **2009,** 11 (26), 5302-5309.
